# Apoptotic Cancer Cell-Primed Cancer-Associated Fibroblasts Suppress Immunosuppressive Macrophages via WISP-1-Integrin α5β3-STAT1 Signaling in Lung Cancer

**DOI:** 10.7150/ijbs.124282

**Published:** 2026-01-01

**Authors:** Kyungwon Yang, Kiyoon Kim, Hee Ja Kim, Jeesoo Chae, Ye-Ji Lee, Shinyoung Kim, Young-Ho Ahn, Jihee Lee Kang

**Affiliations:** 1 Department of Physiology, College of Medicine, Ewha Womans University, Seoul 07804, Korea.; 2 Inflammation-Cancer Microenvironment Research Center, College of Medicine, Ewha Womans University, Seoul 07804, Korea.; 3 Department of Biochemistry, College of Medicine, Ewha Womans University, Seoul 07985, Korea.; 4 Department of Molecular Medicine, College of Medicine, Ewha Womans University, Seoul 07985, Korea.

**Keywords:** TAMs, CAFs, apoptotic cancer cells, WISP-1, Integrin α5β3, STAT1

## Abstract

Cell death within the tumor microenvironment (TME) plays a pivotal role in shaping tumor-specific immunity. The dynamic interplay between cancer-associated fibroblasts (CAFs) and tumor-associated macrophages (TAMs) is central to tumor progression and immune regulation. Here, we show that conditioned medium (CM) from lung CAFs exposed to apoptotic cancer cells selectively impairs the survival of M2-like macrophages, induces apoptosis, and promotes their reprogramming toward an M1-like phenotype. These effects were abrogated by knockdown of Wnt-induced signaling protein 1 (WISP-1) in CAFs, identifying WISP-1 as a key paracrine effector. Mechanistically, WISP-1 signals through the integrin α5β3-STAT1 axis to mediate M2 TAM apoptosis and M1-like reprogramming. *In vivo*, intratumoral injection of CM derived from CAF exposed to apoptotic 344SQ cells reduced overall TAM density, decreased the proportion of M2-like TAMs, and promoted their reprogramming toward an M1-like phenotype, accompanied by STAT1 activation in M2 TAMs. This phenotypic shift was associated with increased infiltration of cytotoxic CD8^+^ T cells and reduced accumulation of regulatory T cells within the tumor. Notably, these effects were abolished by either depletion of WISP-1 from the CM or pharmacological inhibition of STAT1 following recombinant WISP-1 administration. Collectively, our findings identify the WISP-1-integrin α5β3-STAT1 axis as a novel therapeutic target for TAM reprogramming and tumor suppression in lung cancer.

## Introduction

Lung cancer remains the leading cause of cancer-related mortality worldwide 1. Two-thirds of patients are diagnosed at an advanced stage, with metastatic disease, leading to a low 5-year survival rate 2. To improve patient survival rates, new approaches such as targeted therapies and immune checkpoint inhibitors have been developed in recent decades 3. However, these strategies have not yet achieved the desired success. Successful treatment of lung cancer necessitates innovative therapeutic strategies and a more comprehensive understanding of cancer progression and metastasis.

The tumor microenvironment (TME) consists of various non-cancerous components, including immune cells, fibroblasts, capillaries, the basement membrane, and extracellular matrix (ECM), all of which collectively support tumor survival, growth, and invasion 4-6. Among these, cancer-associated fibroblasts (CAFs) are the most abundant stromal cells and exhibit migratory and contractile features reminiscent of myofibroblasts. CAFs are a heterogeneous population arising from diverse cellular origins and secrete a wide range of factors—such as cytokines, chemokines, growth factors, miRNAs, exosomes, and metabolites—that influence cancer cell behavior and the surrounding stroma 7, 8. Through these paracrine signals, CAFs promote tumor progression by enhancing angiogenesis, proliferation, survival, and metastasis 8.

Tumor-associated macrophages (TAMs), a major leukocyte population in lung cancer, are integral to the cancer immune microenvironment, exerting diverse effects on lung tumor growth, progression, and metastasis 9. TAMs undergo dynamic phenotypic changes driven by the TME. Early in tumor development, M1-like TAMs are activated and secrete chemokines and cytokines that recruit cytotoxic CD8^+^ T cells and NK cells, which produce IFN-γ and other factors to eliminate tumor cells 10, 11. As the tumor progresses, M2-like TAMs facilitate cancer growth and spread by suppressing anti-tumor immunity. They secrete factors like TGF-β and upregulate PD-L1 to inhibit T cell activity 12-15. Recent studies underscore the significance of immune cell-stromal cell communication, notably with CAFs, in tumorigenesis. CAFs recruit macrophages to the TME via paracrine signaling in various murine models, including breast, prostate, and squamous cell carcinomas 16-18. Furthermore, CAFs induce a phenotypic shift in M1 macrophages toward an M2-like phenotype within the TME 19.

Apoptotic cell clearance by tissue macrophages and nonprofessional phagocytes is crucial for maintaining tissue homeostasis, immune regulation, and resolution of inflammation. In the TME, where cell death is frequently elevated, the mechanisms governing the removal of dying tumor cells critically influence tumor-specific immunity 20, 21. Efferocytosis, coupled with the release of wound-healing and immunosuppressive cytokines, can promote tumor progression by enabling immune evasion. In contrast, our previous study showed that CAFs reprogrammed by apoptotic cancer cells suppress tumor cell migration and invasion through the secretion of Wnt-induced signaling protein (WISP-1) 22. Injection of conditioned medium (CM) from apoptotic cancer cell-exposed CAFs reduced primary tumor growth and lung metastasis in a WISP-1-dependent manner 22, 23. However, the specific role of reprogrammed CAFs in modulating TAMs within the TME remains poorly defined. In particular, the mechanistic link between CAF-derived WISP-1 and TAM phenotype or survival is not well understood. In this study, we demonstrate that CAFs reprogrammed by apoptotic lung cancer cells suppress tumor-supportive TAMs (M2) by reducing their viability, inducing apoptosis, and promoting reprogramming toward an immune-stimulatory M1-like phenotype. Mechanistically, WISP-1 secreted by reprogrammed CAFs engages integrin α5β3 on M2 macrophages, activating signal transducer and activator of transcription 1 (STAT1) signaling to mediate these effects. Pharmacological inhibition of STAT1 with fludarabine abrogates WISP-1-induced M2 TAM apoptosis and reprogramming, thereby enhancing anti-tumor immunity. These findings identify the WISP-1-integrin α5β3-STAT1 axis as a key regulator of TAM survival and plasticity, highlighting its potential as a therapeutic target in lung cancer.

## Materials and Methods

### Reagents

Fludarabine was obtained from Tocris Bioscience (Bristol, UK). Mouse recombinant WISP-1 (rWISP-1; #1680-WS), human recombinant WISP-1 (*h*rWISP-1; #1627-WS), mouse WISP-1- neutralizing antibodies (MAB1680), and IgG (MAB0061) were purchased from R&D Systems (Minneapolis, MN, USA).

### CAF isolation and cell culture

CAFs were isolated from lung tumors of Kras-mutant (*Kras*LA1) mice using magnetic-activated cell sorting (MACS) with the fibroblast-specific surface marker Thy1, as previously reported 22. In our previous study, we showed that Thy1^+^ CAFs display reduced cell surface areas and an elongated spindle-like morphology—hallmarks of activated fibroblasts—when compared with normal lung fibroblasts shapes, which are regarded as a typical characteristic of activated fibroblasts, compared with normal lung fibroblasts 24, 25. Human Thy1^+^ CAFs (*h*CAFs) were isolated from previously untreated, nonmetastatic primary lung tumors 25. CAFs were maintained in alpha-minimum essential medium (alpha-MEM) supplemented with 10% fetal bovine serum (FBS), penicillin/streptomycin (100 U/100 μg), 2 mM L-glutamine, and 1 mM sodium pyruvate (Welgene, Gyeongsan, Korea). The human lung cancer cell line A549 was obtained from the American Type Culture Collection (ATCC, Manassas, VA). The murine lung cancer cell line 344SQ (a generous gift from Dr. Jonathan M. Kurie, MD Anderson Cancer Center, Houston, TX, USA) and A549 were maintained in RPMI 1640 (HyClone^TM^, GE Healthcare, Boston, MA, USA) supplemented with 10% FBS and penicillin/streptomycin (100 U/100 μg).

### Induction of cell death

Lung cancer cell lines were subjected to ultraviolet (UV) irradiation at a wavelength of 254 nm for 15 min, then incubated in RPMI-1640 medium containing 10% FBS for 2 h at 37 °C and 5% CO_2_. Apoptotic characteristics were identified by examining nuclear morphology using light microscopy on Wright-Giemsa-stained cells, as previously described 26. Necrotic (lysed) cancer cells were generated by subjecting the cells to repeated freeze-thaw cycles. The induction of apoptosis and necrosis was validated using Annexin V-FITC and propidium iodide (PI) staining (BD Biosciences, San Jose, CA, USA), followed by flow cytometric analysis performed on a FACSCalibur flow cytometer (BD Biosciences) 22, 23, 26.

### Preparation of CAF CM

CAFs were seeded at 3 × 10^5^ cells/ml and maintained at 37 °C in a humidified incubator with 5% CO_2_. After overnight culture, cells were serum-deprived by replacing the medium with X-VIVO 10 (04-380Q, Lonza, Basel, Switzerland) for 24 h prior to stimulation. For stimulation, the medium was substituted with X-VIVO 10 supplemented with either apoptotic or necrotic cancer cells at a concentration of 9 × 10⁵ cells/ml. After 20 h co-culture, the medium was harvested and centrifuged at 2,000 × g for 20 minutes to eliminate residual cell debris and apoptotic bodies. The supernatant then passed through a 220 nm pore-size filter and used as the CM to stimulate target epithelial cancer cells (5 × 10^3^ cells/ml). For *in vivo* experiments, CM was stored at -80 °C until required until use.

### Polarization of THP-1-derived macrophages and BMDMs

THP-1 cells were cultured in RPMI 1640 medium supplemented with 10% FBS at 37°C in a humidified atmosphere with 5% CO_2_. THP-1-derived M1 or M2 macrophages were generated as previously described 27. Briefly, THP-1 cells were primed with 150 ng/ml phorbol 12-myristate 13-acetate (Sigma-Aldrich) for 6 h to induce unpolarized macrophages (M0). To establish M1 macrophages, the unpolarized macrophages were stimulated with 20 ng/ml of IFN-γ (R&D Systems) and 100 ng/ml of lipopolysaccharide (Sigma-Aldrich) for an additional 48 h. To establish M2 macrophages, the unpolarized macrophages were stimulated with 20 ng/ml IL-4 and 20 ng/ml IL-13 (R&D Systems) for an additional 48 h. Following polarization, cells were harvested for immunoblot analysis or fixed for immunofluorescent staining.

BMDMs were isolated from the tibias and femurs of C57BL/6 mice and cultured with L929 complement DMEM for 7 days. Subsequently, BMDMs were polarized into M1- and M2-type macrophages according to established protocols 28, 29.

### Cell viability assay

Macrophages (3.5 × 10^4^) were plated into 96-well plates (SPL, Pocheon, Korea) with RPMI-1640 or X-VIVO 10 medium (Lonza, Basel, Switzerland) for 6 h. CM or rWISP-1 was added to each group. Plates were incubated at 37°C with 5% CO_2_ for 1-5 days. Subsequently, Cell Counting Kit-8 (CCK-8) solution (Dojindo Molecular Technologies, Rockville, MD, USA) was added to the wells, and the plates were further incubated at 37°C with 5% CO_2_ for 30 min. Absorbance was measured at 450 nm using a microplate reader.

### Apoptosis assay

For the apoptosis assay, an Annexin V-FITC/PI staining kit (BD Biosciences) was utilized according to the manufacturer's instructions. Macrophages positive for Annexin V-FITC were detected using flow cytometry (ACEA NovoCyte, San Diego, CA, USA). Data analysis was conducted using NovoExpress software 1.5.

Additionally, primary tumor tissues were stained using a TUNEL kit (Roche, Basel, Switzerland) following the manufacturer's instructions. Apoptotic cells were visualized using a confocal microscope (LSM5 PASCAL) equipped with a filter set with excitation wavelengths of 488 and 543 nm. Quantitative analysis of TUNEL-positive M1 or M2 TAMs (TUNEL^+^/ CD206^+^ or TUNEL^+^/CD16/CD32^+^) was performed by manually counting the number of TUNEL-positive cells per field in five randomly selected high-power fields per section in a blinded manner; values were averaged for each mouse.

### Immunoblotting analysis

Whole-cell lysates were prepared from CD11b^+^ TAMs, and from M1- and M2-polarized macrophages derived from THP-1 or BMDMs. Cells were collected, washed with ice-cold PBS, and lysed on ice for 30 minutes in radioimmunoprecipitation assay (RIPA) buffer [10 mM Tris-HCl (pH 7.2), 150 mM NaCl, 1% Nonidet P-40, 0.5% sodium deoxycholate, 0.1% SDS, 1.0% Triton X-100, and 5 mM EDTA] supplemented with a protease inhibitor cocktail. Equal amounts of protein were resolved by SDS-polyacrylamide gel electrophoresis (SDS-PAGE; #161-0158, Bio-Rad Laboratories, Hercules, CA, USA) and transferred to nitrocellulose membranes (10600001, GE Healthcare Life Science, Piscataway, NJ, USA) using a wet transfer system (Bio-Rad Laboratories). Membranes were blocked in 5% bovine serum albumin (BSA) or 5% non-fat dry milk in TBST for 1 h and then incubated overnight at 4°C with the appropriate primary antibodies. After washing, membranes were incubated with HRP-conjugated secondary antibodies for 1 hour at 37°C. Detection was performed using an enhanced chemiluminescence (ECL) kit (Thermo Fisher Scientific, Waltham, MA, USA). Protein bands were visualized using either an ImageQuant LAS 4000 mini (GE Healthcare, Chicago, IL, USA), Amersham ImageQuant 800 (Cytiva, Marlborough, MA, USA), or Agfa X-ray films (PDC Healthcare, Valencia, CA, USA). Band intensities were quantified using ImageJ software (version 1.37; NIH, Bethesda, MD, USA), and normalized to β-actin as a loading control. Antibody information is provided in Table S1.

### Co-immunoprecipitation (CoIP)

THP-1-derived M2 macrophages were lysed in a buffer containing 50 mM Tris-HCl (pH 7.8), 137 mM NaCl, 1 mM EDTA, 1% Triton X-100, 10% glycerol, and a protease inhibitor cocktail. The lysates were clarified by centrifugation at 14,000 rpm for 15 min. The supernatants were then incubated overnight at 4°C with either anti-WISP-1 antibody (Abcam, ab260036; 2 μg/ml) or control IgG (Invitrogen, 02-6102; 2 μg/ml). Following antibody binding, Protein A/G agarose beads (Santa Cruz, sc-2003) were added and allowed to bind for 4 h at 4 °C. The resulting immune complexes were collected and washed three times with lysis buffer. Bound proteins were eluted by boiling the bead pellets in SDS-PAGE sample buffer (50 mM Tris-HCl, pH 6.8; 2% SDS; 10% glycerol; 1% β-mercaptoethanol; 0.1% bromophenol blue) at 95 °C for 10 min, and subsequently analyzed by immunoblotting.

### qRT-PCR arrays

To profile the expression of genes associated with M1 and M2 phenotypes in isolated CD11^+^ TAMs and THP-1-derived M2 macrophages, we used the GeneQuery™Mouse and Human Macrophage Polarization Markers qPCR Array kits (ScienCell, Carlsbad, CA, USA). RNA isolation, DNase treatment, and RNA cleanup were performed according to the manufacturer's instructions (Invitrogen). Isolated RNA was reverse transcribed into cDNA using an RT^2^ First Strand Kit (Qiagen). PCR was conducted using RT^2^ SYBR Green qPCR Master Mix (Qiagen) on a QuantStudio™3 Real-Time PCR System and ABI PRISM 7900 instrument (Applied Biosystems). Expression data were normalized to the average Ct values of glyceraldehyde 3-phosphate dehydrogenase (*Gapdh*), as the housekeeping gene in the array. Each assay was performed in triplicate.

### Quantitative reverse transcription-PCR (qRT-PCR)

Total RNA was extracted from CD11^+^ TAMs and from 1- and M2-polarized macrophages derived from THP-1 or BMDMs utilizing TRIzol reagent (RNAiso plus, Takara Bio Inc., Kusatsu, Japan), and cDNA synthesis was conducted using AccuPower RT PreMix (Bioneer, Daejeon, Korea) according to the manufacturer's protocol. Quantitative real-time PCR was performed with SYBR Green dye on a QuantStudio™ 3 Real-Time PCR System (Applied Biosystems, Foster City, CA, USA). Gene expression levels were normalized to hypoxanthine-guanine phosphoribosyltransferase (*Hprt*) mRNA and expressed as fold changes relative to the control group. Primer sequences are listed in Table S2.

### Transient transfection

CAFs and macrophages were transiently transfected with specific siRNAs targeting WISP-1 (Bioneer), STAT1 (Bioneer), integrin αν (Dharmacon, Horizon Discovery, CO, USA), α5 (Dharmacon), β3 (Dharmacon), β5 (Dharmacon), or control siRNA (SN-1003 AccuTarget^TM^ Negative Control; Bioneer) at a final concentration of 50 nM using Lipofectamine RNAi MAX (Invitrogen, Carlsbad, CA) as per the manufacturer's instructions, respectively. The siRNA sequences are listed in Table S3.

### ELISA

Levels of TNF-α, IL-1β, IL-4, and IL-13 in culture medium from macrophages were measured using ELISA kits (R&D Systems) following the manufacturer's instructions.

### Neutralization of WISP-1 in CM

The CM derived from CAFs was incubated for 2 h with either 10 μg/ml of mouse anti-mouse WISP-1 neutralizing antibody (R&D Systems) or an equivalent concentration of IgG isotype control (R&D Systems). The efficiency of WISP-1 verified using a WISP-1 ELISA before utilization.

### Mouse experiments

All animal procedures were reviewed and approved by the Animal Care Committee of the Ewha Medical Research Institute (Protocol No. EWHA MEDIACUC 22-015-1/2) and conducted in accordance with the guidelines provided by the National Institutes of Health Guide for the Care and Use of Laboratory Animals.

To establish subcutaneous syngeneic tumor models, 8-week-old male 129/Sv mice were injected subcutaneously in the right posterior flank with 1 × 10⁶ 344SQ cells suspended in 100 μl of phosphate-buffered saline (PBS) (n = 6-9 per group) 22, 23. Beginning two days post-inoculation, CM derived from CAFs was administered directly into the tumor site via intratumoral injection three times per week. In separate groups, CM was pretreated with 10 μg/ml of either mouse anti-WISP-1 neutralizing antibody or an isotype IgG control, and administered following the same schedule (n = 6 per group). rWISP-1 was also administered via intratumorally at doses of 12.5 or 25 μg/kg, three times weekly starting 2 days after tumor cell implantation (n = 6 mice per group) 23. To pharmacologically inhibit STAT1 signaling, fludarabine (10 mg/kg in DMSO, 100 μl) was administered intraperitoneally in conjunction with rWISP-1 injection (25 μg/kg) 23. Tumor progression was monitored daily, and all mice were euthanized 6 weeks after tumor implantation. Tumors were excised, measured, and processed for histological analysis, including formalin fixation, paraffin embedding, and immunofluorescence staining. All experiments were conducted using age-matched male mice.

### Isolation of CD11b^+^ TAMs from primary tumors

Single-cell suspensions were prepared from mouse tumors based on a previously established protocol with minor modifications 30. Freshly excised tumors were enzymatically dissociated in RPMI-1640 medium containing 1× collagenase /hyaluronidase and supplemented with 4 U/ml DNase I. The resulting cell mixtures were passed sequentially through 70-μm and 40-μm sterile nylon mesh filters to remove debris. Red blood cells were lysed using a commercial lysis buffer. Following brief pulse centrifugation, the turbid supernatant containing tumor-infiltrating leukocytes was collected. For macrophage enrichment, TAMs expressing CD11b were isolated using CD11b MicroBeads (Miltenyi Biotec, Auburn, CA, USA) according to the manufacturer's instructions. The purified CD11b^+^ cells were cultured in complete DMEM (Corning, Glendale, AZ, USA) supplemented with 20% FBS, 2 mM L-glutamine, 2 mM sodium pyruvate, 55 μM 2-mercaptoethanol, and 1% penicillin-streptomycin. qRT-PCR analysis of freshly isolated, MACS-purified CD11b^+^ macrophages demonstrated a purity consistently greater than 90%. Cells were obtained from two or three randomly selected primary tumors in experimental group.

### Immunofluorescent staining

Immunofluorescent staining was performed in cultured macrophages and primary tumor tissue. Macrophages (10^6^ cells/well) cultured on glass coverslips until confluent were fixed in 4% paraformaldehyde for 8 min at room temperature.

Paraffin-embedded tumor tissues were first fixed in formalin at room temperature for 30 minutes. Following fixation, samples were washed with an immunofluorescent wash buffer consisting of 0.05% sodium azide, 0.1% BSA, 0.2% Triton X-100, and 0.05% Tween-20 in PBS. Three consecutive washes were performed using this buffer for 5 minutes each. Permeabilization was then carried out using 0.5% Triton X-100 (Sigma-Aldrich) in PBS for 5 minutes at room temperature. For immunohistochemical staining, sections were blocked with 5% BSA in PBS. For immunocytochemical applications, 5% BSA in PBS with or without a mouse IgG blocking reagent was used. After a 1-hour blocking step at room temperature, samples were incubated overnight at 4°C with primary antibodies specific to the target proteins. Fluorescent labeling was achieved by incubating the samples with appropriate fluorophore-conjugated secondary antibodies in the dark for 1 hour. Nuclei were counterstained using VECTASHIELD mounting medium containing DAPI (Vector Laboratories, Burlingame, CA, USA), and imaging was performed using a confocal microscope (LSM5 PASCAL, Carl Zeiss, Jena, Germany). Detailed information on the antibodies, including sources and working dilutions, is provided in Table S1.

### Flow cytometry analysis of the immune cell population

CD11b^+^ cells isolated from primary tumors were fixed with 4% paraformaldehyde and incubated in the dark at 4 °C for 30 minutes with either fluorophore-conjugated antibodies or unconjugated primary antibodies. For staining with unconjugated antibodies (anti-CD163, anti-CD206, anti-MHCII, and anti-CD80), cells were washed and subsequently incubated with appropriate fluorophore-conjugated secondary antibodies. Flow cytometric analysis was performed on at least 10,000 events per sample using a flow cytometer (ACEA NovoCyte, San Diego, CA, USA). Data were analyzed using NovoExpress software 1.5. Tumor-infiltrating leukocytes obtained after pulse centrifugation were resuspended in PBS containing FBS and processed similarly for flow cytometry. The gating strategy for immune cell populations is detailed in Supplementary Fig. S17, and antibody details are listed in Table S4.

### Statistics

Pairwise comparisons were conducted using two-tailed Student's *t*-tests, and multiple comparisons were analyzed by one-way ANOVA followed by Tukey's post hoc test. *P* values < 0.05 were considered statistically significant. All statistical analyses were performed using Prism 5 software (GraphPad Software Inc., San Diego, CA, USA). Pearson correlation analysis was employed for simple linear correlation analyses.

## Results

### CM from CAFs exposed to apoptotic cancer cells decreases M2 macrophage survival and drives M1-like reprogramming

We previously observed that the *in vivo* anti-tumor effect of CM from CAFs exposed to apoptotic 344SQ cells (ApoSQ-CAF CM) markedly exceeded its direct antiproliferative activity on lung cancer cells *in vitro* 24. This finding suggested that ApoSQ-CAF CM may exert additional indirect effects within the TME, potentially through modulation of TAMs—a key immunosuppressive and pro-tumorigenic component of the TME. To explore this possibility, we directly treated M1 and M2 macrophages with ApoSQ-CAF CM *in vitro*. THP-1 cells and primary mouse bone marrow-derived macrophages (BMDMs) were polarized into M1 or M2 phenotypes to model TAM populations (Supplementary Fig. S1a, d). Immunoblot analysis confirmed successful polarization, with M2 markers (CD163, CD206, and Arginase 1) and M1 markers (MHCII, iNOS, and IL12p40) expressed as expected (Supplementary Fig. S1b, e). Confocal microscopy further validated the phenotypes, showing CD86^+^ cells as M1 and CD163^+^ cells as M2 macrophages (Supplementary Fig. S1c, f).

**Figure 1:**
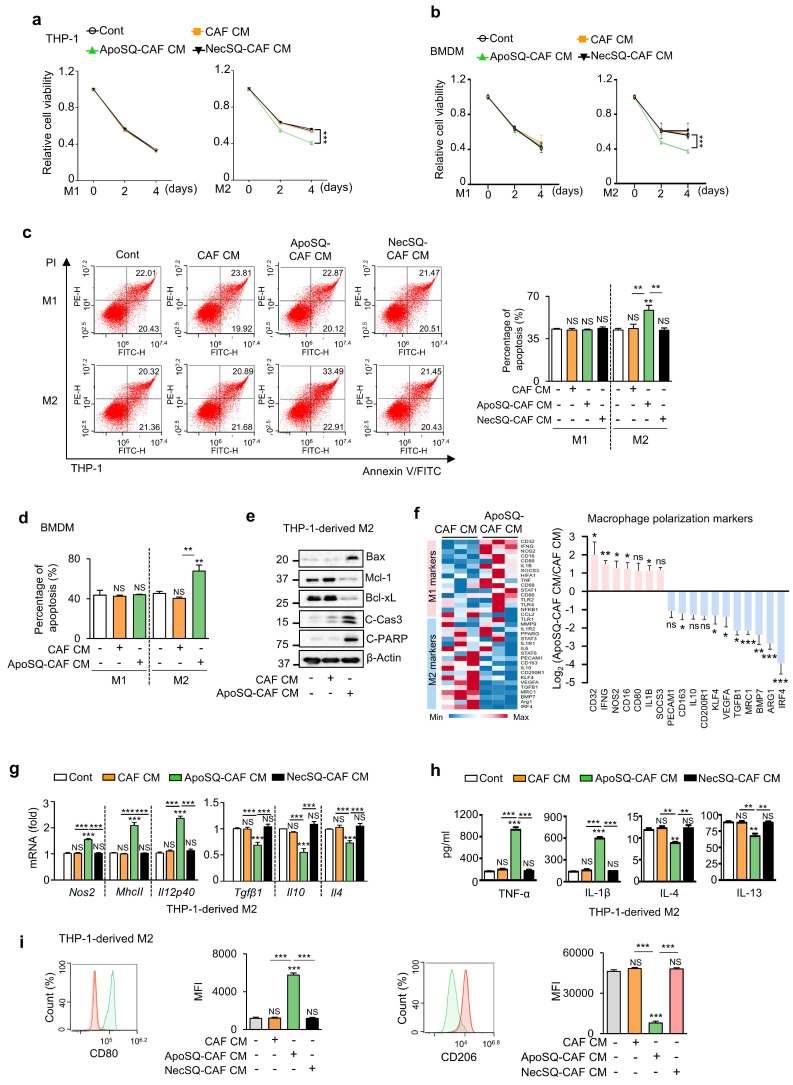
** CM from CAFs exposed to apoptotic cancer cells reduces M2 macrophage survival, induces apoptosis, and promotes reprogramming toward an M1 phenotype *in vitro*.** (**a, b**) Cell viability assay of M1 (M1) or M2 macrophages (M2) derived from THP-1 cells and BMDMs. (**c, d**) Apoptotic M1 or M2 macrophages were quantified as the sum of the percentages of early and late stages of apoptosis. Flow cytometry analysis after Annexin V-FICT/PI dual staining was employed to evaluate apoptosis. (**e**) Immunoblot analysis of Bax, Mcl-1, Bcl-xL, cleaved caspase-3, cleaved PARP, and β-actin in THP-1-derived M2 macrophages. (**a-e**) CAFs were exposed to apoptotic 344SQ cells (ApoSQ) or necrotic cancer cells (NecSQ) for 20 h. Conditioned medium from CAFs only (CAF CM), exposed to ApoSQ (ApoSQ-CAF CM) or NecSQ (NecSQ-CAF CM) was treated to THP-1- or BMDM-derived M1 and M2 macrophages for the indicated days (**a**,** b**), or 3 days (**c-e**). (**f**) Heatmap showing differentially expressed macrophage polarization-related genes in THP-1-derived M2 macrophages treated with CM for 3 days (*left*). Red: high expression; blue: low expression. Relative expression of selected genes from PCR array profiling of macrophage polarization markers (*right*). Log2 fold-change values (ApoSQ-CAF CM vs. CAF CM). (**g**) qRT-PCR analysis of relative mRNA levels of M1 (*Nos2*, *MhcII*, and *Il12p40*) and M2 (*Tgfβ1*, *Il10*, and *Il4*) markers in THP-1-derived M2 macrophages treated with CM for 3 days. (**h**) ELISA of TNF-α, IL-1β, IL-4, and IL-13 in the culture supernatant of M2 macrophages treated with CM for 3 days. (**i**) Flow cytometry analysis of the population of CD80^+^ and CD206^+^ cells among M2 macrophages derived from THP-1 cells for 2 or 3 days. Mean fluorescence intensity (MFI) values (*right*). NS, not significant; *P < 0.05, **P < 0.01, ***P < 0.001, two-tailed Student's *t*-test. Data are from one experiment representative of three independent experiments with similar results (**c**,** f** and** i *left***; **e**) or from three independent experiments (mean ± standard error: **a**, **b**, **d**, **g**,** h**; **c**,** f** and** i *right***).

For viability assessment, M1- and M2-polarized THP-1 cells and BMDMs were treated with CM for 4 days under serum-free conditions, followed by CCK-8 assays. CM from CAFs, with or without exposure to ApoSQ or necrotic 344SQ cells (NecSQ), had no effect on the viability of M1 macrophages on days 2 and 4 (Fig. 1a, b). However, treatment of M2 macrophages with ApoSQ-CAF CM reduced cell viability, whereas CAF CM and NecSQ-CAF CM had no effect. Flow cytometric analysis after Annexin V-FITC- PI staining revealed that CM from CAFs, regardless of ApoSQ or NecSQ exposure, did not affect apoptosis in M1-polarized THP-1 cells and BMDMs on day 4 (Fig. 1c, d and Supplementary Fig. S2a). However, ApoSQ-CAF CM enhanced the apoptosis in M2 macrophages, whereas CAF CM and NecSQ-CAF CM had no effect. In addition, ApoSQ-CAF CM treatment of THP-1-derived M2 macrophages increased expression of pro-apoptotic biomarkers, including Bax, cleaved caspase-3, and cleaved PARP, and decreased expression of the anti-apoptotic proteins Mcl-1 and Bcl-xL compared with CAF CM (Fig. 1e). Notably, the viability and apoptosis of unpolarized macrophages (M0) derived from THP-1 cells were unaffected by any of the CM types (Supplementary Fig. S2b, c).

Next, we investigated the ability of ApoSQ-CAF CM to induce M2-to-M1 macrophage reprogramming *in vitro*. A targeted RT-qPCR array revealed that eleven M2-related genes, including *Irf4*, *Arg1, Bmp7, Mrc1, Tgfb1, Vegfa, Klf4, Cd200r1, Il10, Cd163,* and* Pecam1,* were downregulated (>2-fold) in the ApoSQ-CAF CM group compared to the CAF CM group (Fig. 1f). In contrast, seven M1-related genes, including *Cd32*, *Ifng*, *Nos2, Cd16*, *Cd80, IL1b*, and *Socs3*, were upregulated (>2 fold). qRT-PCR analysis further confirmed that ApoSQ-CAF CM downregulated M2 markers (*Tgfβ1*,* Il10*, *Il4*) and upregulated M1 markers (*Nos2*, *MhcII*, and *Il12p40)* in M2-polarized THP-1 cells and BMDMs, while CAF CM or NecSQ-CAF CM had no effect (Fig. 1g and Supplementary Fig. S2d). Consistently, ApoSQ-CAF CM increased M1 cytokine levels (TNFα, IL-1β) and reduced M2 cytokines (IL-4, IL-13) in the culture supernatant of M2-polarized THP-1 cells (Fig. 1h). Flow cytometry further revealed a reduction in the M2 surface marker CD206 and an increase in the M1 marker CD80 in M2-polarized THP-1 cells and BMDMs treated with ApoSQ-CAF CM compared to those treated with CAF CM (Fig. 1i and Supplementary Fig. S1e). In addition, qRT-PCR, ELISA, and flow cytometric analyses revealed no changes in the expression of M1 or M2 markers in unpolarized (M0) THP-1 cells following treatment with any of the CM types (Supplementary Fig. S2f-h).

Similarly, ApoA-CAF CM (CM from CAFs exposed to apoptotic A549 cells) and ApoA-*h*CAF CM (CM from *h*CAFs exposed to ApoA) reduced cell viability and induced apoptosis in THP-1-derived M2 macrophages, while having no effect on M1 macrophages (Supplementary Fig. S3a-d). Both CM types also promoted reprogramming of M2 macrophages toward an M1-like phenotype (Supplementary Fig. S4a-f). Collectively, these findings demonstrate that CM from apoptotic cancer cell-primed CAFs does not affect M0 or M1 macrophages, but selectively impairs the survival of M2 macrophages, induces apoptosis, and promotes reprogramming toward an M1-like phenotype.

### ApoSQ-CAF CM activates STAT1 signaling, inhibiting M2 macrophage survival and inducing M2 macrophage reprogramming

**Figure 2:**
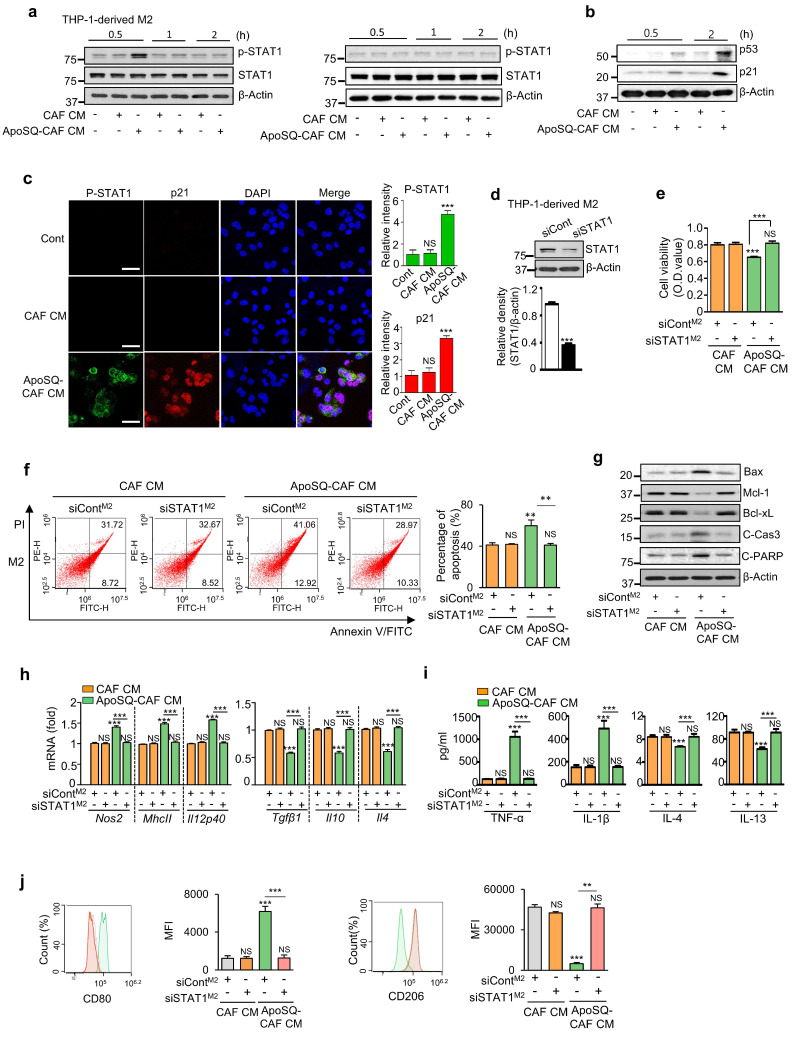
** ApoSQ-CAF CM activates STAT1 in M2 macrophages.** (**a, b**) Immunoblot analysis of the indicated proteins in THP-1-derived M1 (M1) and M2 macrophages (M2) treated with CAF CM or ApoSQ-CAF CM for the indicated time. (**c**) Immunofluorescence staining for phosphorylated STAT1 and p21 (*Left*) and quantitation (*Right*) in M2 macrophages for 1 h after treatment with CAF CM or ApoSQ-CAF CM. The imaging medium was VECTASHIELD fluorescence mounting medium containing DAPI. Original magnification: ×400. Scale bars = 20 μm. (**d**) Immunoblot analysis of STAT1 in M2 macrophages transfected with control or STAT1 siRNA (*upper*). Densitometric analysis of the relative STAT1 abundance (*lower*). (**e**) Cell viability assay of M2 macrophages. (**f**) *Left:* Flow cytometry analysis after Annexin V-FICT/PI dual staining was employed to evaluate the cell apoptosis of M2 macrophages. *Right*: Apoptotic cells were quantified as the sum of the percentages of early and late stages of apoptosis. (**g**) Immunoblot analysis of the indicated proteins in M2 macrophage lysates. (**h**) qRT-PCR analysis of relative mRNA levels of M1 (*Nos2*, *MhcII*, and *Il12p40*) and M2 (*Tgfβ1*, *Il10*, and *Il4*) markers in M2 macrophages (M2). (**i**) ELISA of TNF-α, IL-1β, IL-4, and IL-13 in the culture supernatant of M2 macrophages. (**j**) Flow cytometry analysis of the population of CD80^+^ and CD206^+^ cells among M2 macrophages. Mean fluorescence intensity (MFI) values (*right*). (**e**-**j**) THP-1-derived M2 macrophages were transfected with control or STAT1 siRNA for 24 h before treatment with CM for 2 or 3 days. NS: not significant; **P < 0.01, ***P < 0.001, two-tailed Student's *t*-test. The data are from one experiment representative of three independent experiments with similar results (**a**, **b, g**; **c**, **f**, and **j *left***;** d *upper***) or from three independent experiments (mean ± standard error in **c**, **f**, and **j *right***; **d *lower***; **e**, **h**, **i**).

Given the pivotal role of STAT1 in regulating the phenotype, survival, and function of TAMs within the TME 31, 32, we assessed STAT1 activation in M1 and M2 macrophages following CM treatment. ApoSQ-CAF CM and ApoA-*h*CAF selectively enhanced STAT1 phosphorylation (tyrosine 701) in THP-1-derived M2 macrophages within 30 min, but not in M1 macrophages (Fig. 2a and Supplementary Fig. S4g). This was accompanied by increased levels of p53 and p21 proteins in M2 macrophages compared to CAF CM treatment (Fig. 2b). Immunofluorescence analysis further confirmed increased STAT1 phosphorylation and p21 expression in M2 macrophages treated with ApoSQ-CAF CM, showing nuclear colocalization of p21 with phosphorylated STAT1 (Fig. 2c).

To validate the role of STAT1 *in vitro*, STAT1 signaling was inhibited using STAT1 siRNA or the selective inhibitor fludarabine (1 μM) in THP-1-derived M2 macrophages. STAT1 Knockdown or fludadarabine reversed the anti-survival and pro-apoptotic effects of ApoSQ-CAF CM (Fig. 2d-f and Supplementary Fig. S5a, b), along with changes in apoptosis-related proteins, including Bax, Mcl-1, Bcl-xL, cleaved caspase-3, and cleaved PARP (Fig. 2g and Supplementary Fig. S5c). STAT1 silencing or fludarabine pretreatment abolished the M2-to-M1 reprogramming induced by ApoSQ-CAF CM (Fig. 2h-j and Supplementary Fig. S5d-f). These findings indicate that ApoSQ-CAF CM selectively targets M2 macrophages, reducing survival, promoting apoptosis, and reprogramming through STAT1 signaling.

### WISP-1 is a key mediator of anti-survival, and reprogramming effects

**Figure 3:**
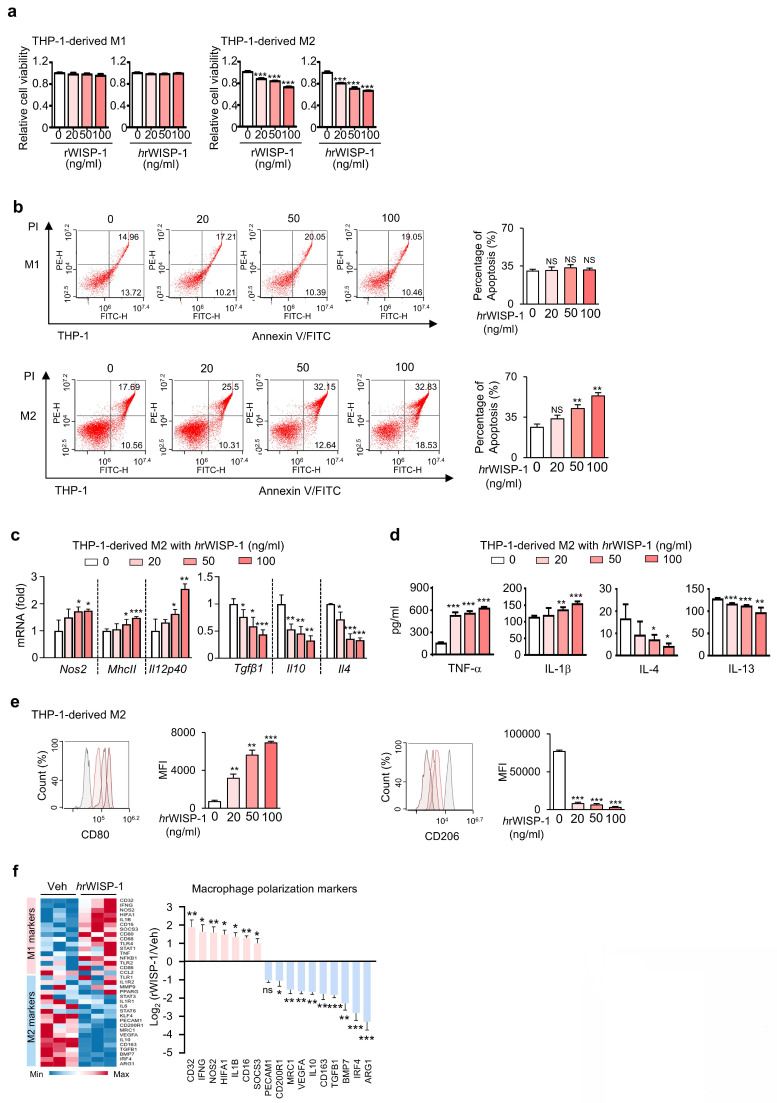
** Recombinant WISP-1 reduces M2 macrophage survival, induces apoptosis, and promotes reprogramming toward an M1-like phenotype.** (**a**) Cell viability assay of THP-1-derived M1 (M1) and M2 macrophages (M2) treated with 20-100 ng/ml mouse (rWISP-1) or human WISP-1 (*h*rWISP-1) for 3 days. (**b**) *Left:* Flow cytometry analysis after Annexin V-FICT/PI dual staining was employed to evaluate the apoptosis of THP-1- derived M1 and M2 macrophages after treatment with *h*rWISP- (20-100 ng/ml) for 3 days. *Right*: Apoptotic cells were quantified as the sum of the percentages of early and late stages of apoptosis. (**c**) qRT-PCR analysis of relative mRNA levels of M1 (*Nos2*, *MhcII*, and *Il12p40*) and M2 (*Tgfβ1*, *Il10*, and *Il4*) markers in M2 macrophages treated with 20-100 ng/ml *h*rWISP-1 for 3 days. (**d**) ELISA of TNF-α, IL-1β, IL-4, and IL-13 in the culture supernatant of M2 macrophages treated with 20-100 ng/ml *h*rWISP-1 for 3 days. (**e**) Flow cytometry analysis of the population of CD80^+^ and CD206^+^ cells among M2 macrophages after treatment with *h*rWISP-1 (20-100 ng/ml) for 2 or 3 days. Mean fluorescence intensity (MFI) values (*right*). (**f**) Heatmap showing differentially expressed macrophage polarization-related genes in THP-1-derived M2 macrophages (*left*). Red: high expression; blue: low expression. Relative expression of selected genes from PCR array profiling of macrophage polarization markers (*right*). Log2 fold-change values (*h*rWISP-1 vs. Vehicle). THP-1-derived M2 macrophages were treated with *h*rWISP-1 (50 ng/ml) for 3 days. NS: not significant; *P < 0.05, **P < 0.01, ***P < 0.001, two-tailed Student's *t*-test. The data are from one experiment representative of three independent experiments with similar results (**b**,** e**, and** f *left***) or from three independent experiments (mean ± standard error: **a**,** c**,** d**; **b**,** e** and** f *right***).

In our prior study, we identified WISP-1 as a key mediator of the anti-tumor and anti-metastatic effects of ApoSQ-CAF CM 22, 23. Building on this, we further investigated the role of WISP-1 in regulating M2 macrophage survival, apoptosis, and reprogramming *in vitro*. Knockdown of WISP-1 in CAFs before treatment with ApoSQ abolished the anti-survival, pro-apoptotic, and reprogramming effects of ApoSQ-CAF CM in THP-1-derived M2 macrophages (Supplementary Fig. S6a-f). To validate the role of secreted WISP-1, THP-1-derived M1 and M2 macrophages were directly treated with recombinant human (*h*rWISP-1) or mouse WISP-1 (rWISP-1; 81% sequence identity). Both rWISP-1 variants (20-100 ng/ml) regardless of species origin, reduced cell viability in M2 macrophages in a dose dependent manner, without affecting M1 macrophages (Fig. 3a). *h*rWISP-1 induced apoptosis of M2 macrophages, with no effect on M1 cells, and promoted M2-to-M1 reprogramming in a dose-dependent manner (Fig. 3b-e), consistent with changes in M1 and M2 gene expression observed by RT-qPCR array (Fig. 3f). Similarly, rWISP-1 reduced cell viability, induced apoptosis in M2-polarized BMDMs without affecting M1 macrophages, while promoting their reprogramming toward an M1-like phenotype (Supplementary Fig. S7a-d).

### WISP-1 interacts with integrin α5β3 to reduce M2 macrophage survival and promote reprogramming toward an M1-like phenotype

**Figure 4:**
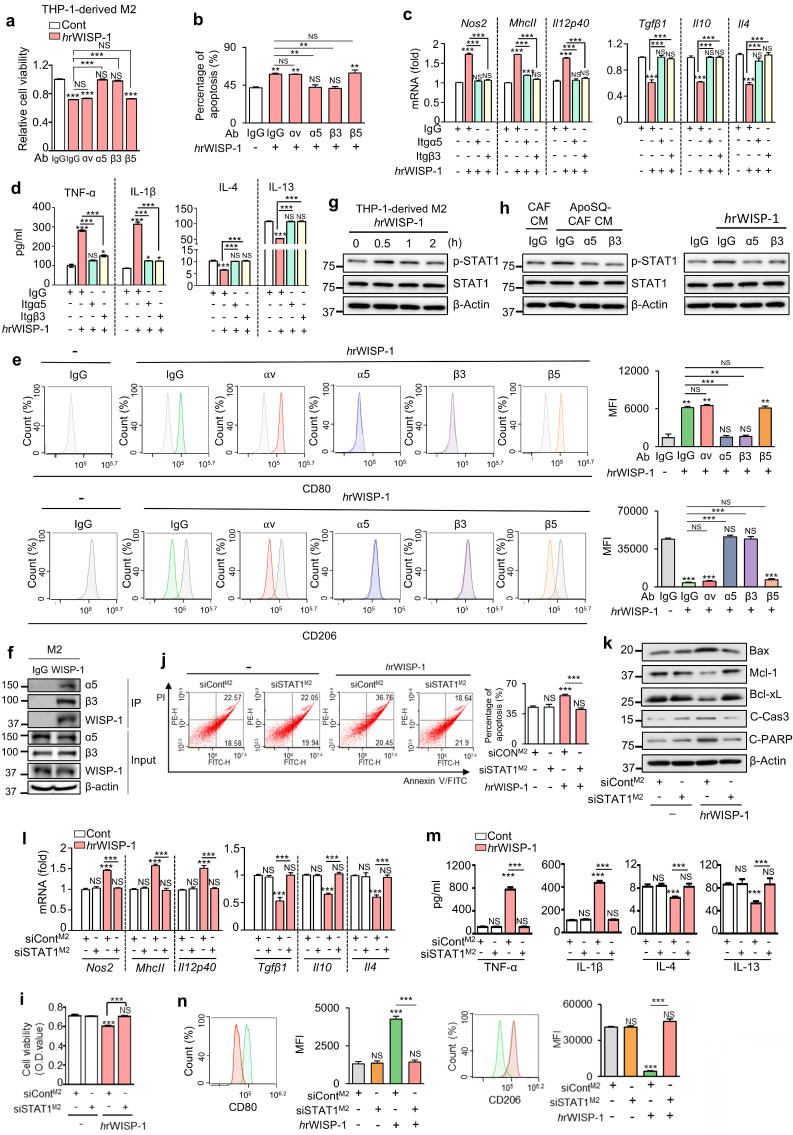
** rWISP-1 acts through integrin α5β3 to activate STAT1 in M2 macrophages.** (**a**, **i**) Cell viability assay of M2 macrophages treated with 50 ng/ml human rWISP-1 (*h*rWISP-1) for 3 days. (**b**,** j**) Apoptotic cells were quantified as the sum of the percentages of early and late stages of apoptosis. Flow cytometry analysis after Annexin V-FICT/PI dual staining was employed to evaluate the cell apoptosis of M2 macrophages treated with *h*rWISP-1 for 3 days. (**c**,** l**) qRT-PCR analysis of relative mRNA levels of M1 (*Nos2*, *MhcII*, and *Il12p40*) and M2 (*Tgfβ1*, *Il10*, and *Il4*) markers in M2 macrophages treated with *h*rWISP-1 for 3 days. (**d**,** m**) ELISA of the cytokines (TNFα, IL-1β, IL-4, and IL-13) in the culture supernatants of M2 macrophages treated with *h*rWISP-1 for 3 days. (**e**, **n**) Flow cytometry analysis of the population of CD80^+^ and CD206^+^ cells among M2 macrophages treated with *h*rWISP-1 for 2 or 3 days. Mean fluorescence intensity (MFI) values (*right*). (**f**) CoIP assays of protein interaction in M2 macrophages. Cell lysates were immunoprecipitated (IP) with anti-WISP-1 and then immunoblotted with anti-integrin α5 and anti-integrin β3 antibodies. (**g**,** h**) Immunoblot analysis of phosphorylated and total STAT1 in THP-1-derived M2 macrophages treated with ApoSQ-CAF CM or *h*rWISP-1 for the indicated time (**g**) or 30 min (**h**). (**k**) Immunoblot analysis of the indicated proteins in M2 macrophages treated with *h*rWISP-1 for 3 days. (**a-e**) THP-1-derived M2 macrophages were pretreated with an anti-integrin blocking antibody (3 μg/ml; anti-integrin αν, α5, β3 or β5) or corresponding IgG isotype control for 30 min before treatment with rWISP-1 (50 ng/ml). (**i-n**) THP-1-derived M2 macrophages were transfected with control or STAT1 siRNA before treatment with *h*rWISP-1 (50 ng/ml). NS: not significant; **P < 0.01, ***P < 0.001, two-tailed Student's *t*-test. The data are from one experiment representative of three independent experiments with similar results (**e**, **j**, and **n *left***;** f**, **g**, **h**,** k**) or from three independent experiments (mean ± standard error: **a-d**, **i**, **l**, **m**;** e**, **j**, and **n *right***).

WISP-1 exerts its function by binding to integrins, which are critical cell surface receptors 33, 34. In previous studies, we identified integrin αv and β3 as key receptors mediating the inhibitory effects of WISP-1 on lung cancer cell migration, invasion, and growth 22, 23. To determine which integrins mediate WISP-1 activity in M2 macrophages, we employed blocking antibodies against integrins αν, α5, β3, and β5 before stimulation with *h*rWISP-1 (50 ng/ml). Blocking α5 or β3 significantly reversed the effects of *h*rWISP-1 on cell viability, apoptosis, and expression of M1 (*Nos2*, *MhcII*, and *Il12p40*) and M2 markers (*Tgfβ1*,* Il10*, and* Il4*) in M2-polarized THP-1 cells, as well as secreted cytokines (TNF-α, IL-1β, IL-4 and IL-13) in culture supernatants compared to control IgG group (Fig. 4a-d and Supplementary Fig. S8a). Flow cytometric analysis further confirmed these reversing effects on CD206 and CD80 surface expression (Fig. 4e). However, blocking αν or β5 had no effect.

To further validate these findings, we silenced αν, α5, β3, or β5 in THP-1-derived M2 macrophages using specific siRNAs (Supplementary Fig. S8b). Knockdown of integrin α5 or β3 significantly attenuated the anti-survival, pro-apoptosis and reprogramming effects of *h*rWISP-1 (Supplementary Fig. S8c-g). In contrast, silencing integrin αν or β5 had no effect on cell viability, apoptosis, or the expression of M1 and M2 markers.

To confirm the role of WISP-1-integrin α5β3 signaling in M2 macrophages treated with ApoSQ-CAF CM, we also used neutralizing antibodies against integrins αv, α5, β3, and β5 in THP-1-derived M2 macrophages. Blocking α5 or β3 significantly attenuated the anti-survival, pro-apoptotic, and reprogramming effects of ApoSQ-CAF CM, while blocking αv or β5 had no effect (Supplementary Fig. 9a-e). These results were further validated by siRNA-mediated knockdown of αv, α5, β3, or β5, which confirmed that only α5 or β3 silencing abrogated the effects of ApoSQ-CAF CM (Supplementary Fig. 10a-e). Collectively, these findings identify integrin α5β3 as the key receptor mediating WISP-1-driven paracrine signaling in M2 macrophages.

Interestingly, integrins α5 and β3 were preferentially expressed in THP-1- or BMDM-derived M2 macrophages compared to M1 macrophages (Supplementary Fig. S11a, b). Immunofluorescent staining in primary tumor sections further demonstrated that integrins α5 and β3 were predominantly expressed in tumor-supportive M2 TAMs, with minimal colocalization with M1 TAM markers (Supplementary Fig. S11c-f). Given that WISP-1 signals through integrin α5β3, this preferential expression in M2 macrophages supports a selective paracrine interaction through which WISP-1 exerts its anti-survival, pro-apoptotic, and reprogramming effects on M2 TAMs. To further confirm that integrin α5β3 serves as a receptor for WISP-1 in M2 macrophages, we performed a CoIP assay to examine their physical interaction. Immunoprecipitation with an anti-WISP-1 antibody successfully pulled down WISP-1, which was co-precipitated with both integrin α5 and β3 in THP-1-derived M2 macrophages (Fig. 4f). Collectively, these data validate that integrin α5β3 acts as the receptor for WISP-1 in paracrine signaling within M2 macrophages.

### WISP-1 signals through the integrin α5β3-STAT1 to suppress immunosuppressive M2 macrophages

Next, we investigated the role of STAT1 activation as the downstream target of WISP-1-integrin α5β3 signaling in THP-1-derived M2 macrophages. Similar to ApoSQ-CAF CM (Fig. 2a) and ApoA-*h*CAF CM (Supplementary Fig. S4g), stimulation with *h*rWISP-1 led to an increase in phosphorylated STAT1 (tyrosine 701) in THP-1-derived M2 macrophages within 30 min (Fig. 4g). Immunoblot analysis further revealed that blocking integrin α5 or β3 — either through neutralizing antibodies or siRNA knockdown—reduced STAT1 activation induced by ApoSQ-CAF CM (Fig. 4h *left* and Supplementary Fig. S12a), ApoA-*h*CAF CM (Supplementary Fig. S12b, c), or *h*rWISP-1 (Fig. 4h* right* and Supplementary Fig. S12d) in THP-1-derived M2 macrophages. Moreover, the anti-survival and pro-apoptosis effects of *h*rWISP-1 in M2 macrophages were abolished flowing STAT1 knockdown (Fig. 4i-k) or pretreatment with fludarabine (1 μM) (Supplementary Fig. S13a-c). The ability of *h*rWISP-1 to reprogram M2 macrophages toward an M1-like phenotype was blocked by STAT1 knockdown (Fig. 4l-n) or fludarabine pretreatment (Supplementary Fig. S13d-f). Collectively, these results indicate that WISP-1 selectively targets M2 macrophages and mediates anti-survival, pro-apoptotic, and M1-like reprogramming effects through activation of the integrin α5β3-STAT1 signaling pathway.

Previously, we reported significant quantitative correlations between CCN4 (WISP-1) and STAT1 expression in both CPTAC- lung adenocarcinoma (LUAD) and TCGA-LUAD (n=510) 23. In particular, *CCN4* expression strongly associated with phospho-STAT1 (S727), suggesting that microenvironmental CCN4 may contribute to STAT1 activation in patient lung cancer. To localize the cellular sources of WISP-1 and STAT1 activity within the lung TME, we reanalyzed the single-cell transcriptomic dataset of Zuani et al. profiling NSCLC (Non-small-cell lung cancer): tissues from 25 patients, focusing on macrophages and fibroblasts (~81,000 cells; Table S5) 35. Canonical marker analysis confirmed that anti-inflammatory alveolar macrophages and STAB1^+^ anti-inflammatory macrophages predominantly expressed M2-associated markers (MRC1, CD163), indicating an M2-like baseline state (Supplementary Fig. S14a). Consistent with our experimental data, WISP-1 was highly enriched in activated adventitial fibroblasts, supporting a stromal origin of WISP-1 in human NSCLC (Supplementary Fig. S14b). In contrast, STAT1 expression was elevated in M2-like macrophage subsets, with the highest levels in STAB1^+^ anti-inflammatory macrophages. Together, these findings support a model in which stromal WISP-1 engages the STAT1 pathway to drive M1-like reprogramming of M2-like TAMs.

### CM from apoptotic lung cancer cell-exposed CAFs inhibits the survival of tumor-supportive TAMs via WISP-1 *in vivo*

**Figure 5:**
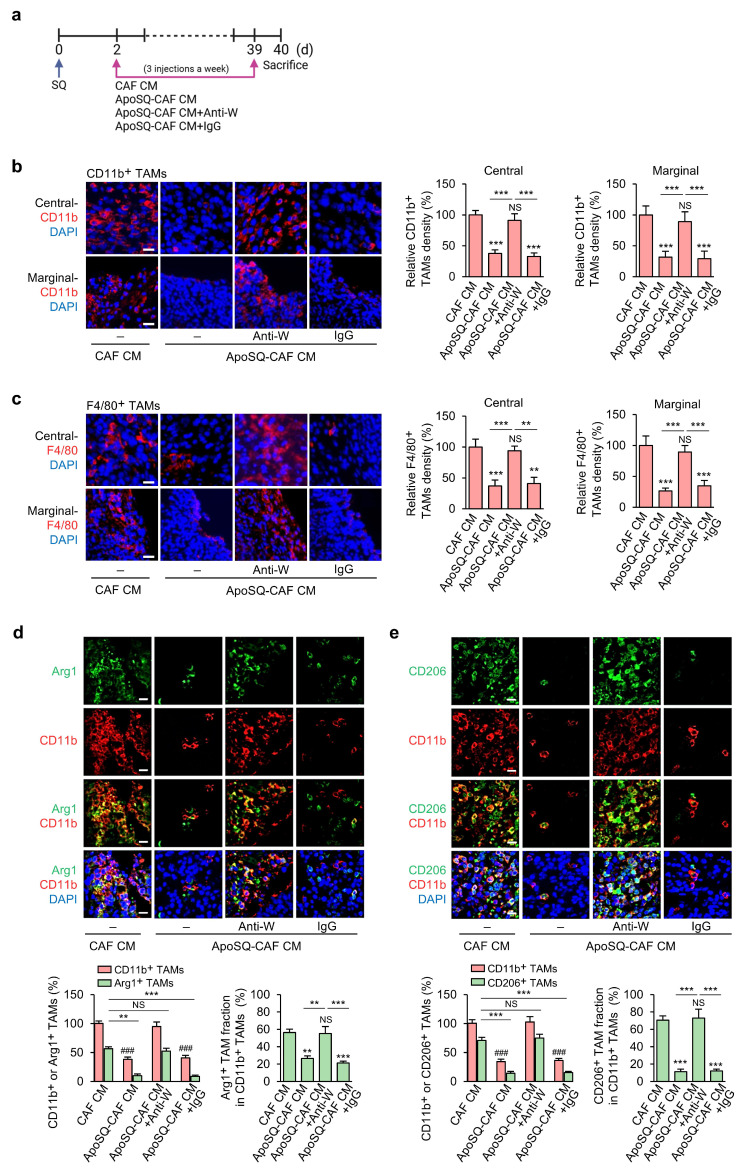
** Administration of ApoSQ-CAF CM reduces TAM density and M2 TAM fraction in primary tumors via WISP-1.** (**a**) Schematic of experimental design and treatment groups. Starting two days after subcutaneous implantation of 344SQ cells into syngeneic (129/Sν) mice, intratumoral injections of conditioned medium from CAFs only (CAF CM), exposed to ApoSQ (ApoSQ-CAF CM) CAF CM, ApoSQ-CAF CM combined with anti-WISP-1, or ApoSQ-CAF CM combined with control IgG were administered three times per week for six weeks (n = 6 mice per group). Mice were necropsied at the end of the 6-week treatment period. (**b**, **c**) *Left*: Immunofluorescent staining of the pan-macrophage marker CD11b (red) and F4/80 (red), along with DAPI (blue), in central and marginal regions of primary tumors. Images were acquired at ×40 magnification. Scale Bar = 100 μm. *Right*: Quantitation of CD11b^+^ and F4/80^+^ TAM density. (**d**, **e**) *Upper*: Immunofluorescent staining of primary tumor sections showing M2 TAM Markers Arg1 (green) and CD206 (green), along with the pan-macrophage marker CD11b (red). Original magnification: ×40. Scale bars = 100 μm. *Lower*: Quantitation of Arg1^+^ and CD206^+^ TAM (M2) density (*left*) and the fraction of M2 TAMs (*right*) in primary tumors. The M2 TAM fraction was determined by the percentage of M2 TAMs within CD11b^+^ TAMs. NS, not significant; **P < 0.01, ***P < 0.001 compared to CAF CM or as indicated; ^###^*P* < 0.001 compared to CAF CM, Analysis of variance with Tukey's post hoc test. The data are from one experiment representative of three independent experiments with similar results (**b** and **c *left***; **d** and **e *upper***). The data are represented as the means ± standard errors from three mice per group (**b** and** c *right***;** d** and** e *lower***).

To investigate the *in vivo* effect of ApoSQ-CAF CM targeting M2 macrophages, we assessed whether it modulates TAM density and subtype distribution to mediate its tumor-suppressive activity. Syngeneic (129/Sν) mice were subcutaneously injected with 344SQ cells, followed by intratumoral administration of either CAF CM or ApoSQ-CAF CM three times per week for six weeks, starting two days after tumor cell implantation (Fig. 5a). Immunofluorescent staining using TAM markers CD11b and F4/80 revealed that ApoSQ-CAF CM substantially reduced total TAM density in both the central and peripheral regions of the primary tumor compared to CAF CM (Fig. 5b, c). To further determine whether WISP-1 is responsible for TAM modulation following ApoSQ-CAF CM treatment *in vivo*, we pre-incubated the CM with either a neutralizing anti-WISP-1 antibody or an IgG isotype control for 2 h prior to intratumoral injection. The reduction in TAM density was abolished in tumors treated with WISP-1-depleted CM, whereas CM containing the isotype control retained the suppressive effect on TAM density. These results indicate that the TAM-reducing activity of ApoSQ-CAF CM is dependent on WISP-1.

TAMs are broadly categorized into two functionally distinct populations: tumor-supportive macrophages (M2 TAMs) and tumor-suppressive macrophages (M1 TAMs) 9, 36, 37. To assess whether ApoSQ-CAF CM alters the TAM subtype distribution within primary tumors, we performed immunofluorescence staining using established M2 markers (Arg1 and CD206) and M1 markers (iNOS, CD80, and CD16/CD32) 28, 31. ApoSQ-CAF CM treatment significantly decreased the proportion of M2 TAMs (Fig. 5d, e; Supplementary Fig. S15a) and concomitantly increased the fraction of M1 TAMs (Supplementary Fig. S15b-d). Notably, these changes were abrogated when ApoSQ-CAF CM was immunodepleted of WISP-1, whereas CM treated with an isotype control antibody retained its effects, indicating a WISP-1-dependent mechanism. Immunofluorescent analysis further revealed that ApoSQ-CAF CM selectively induced apoptosis in M2 TAMs, as evidenced by increased cleaved caspase-3^+^/CD206^+^ cells, while having minimal impact on M1 TAMs (cleaved caspase-3^+^/iNOS^+^) (Supplementary Fig. S16a, b). Consistently, the total number of apoptotic TAMs (cleaved caspase-3^+^/CD11b^+^) was markedly elevated following ApoSQ-CAF CM injection (Supplementary Fig. S16c). TUNEL assays combined with immunohistochemistry further confirmed a selective increase in DNA fragmentation in M2 TAMs (TUNEL^+^/CD206^+^) but not in M1 TAMs (TUNEL^+^/CD16/CD32^+^) (Supplementary Fig. S17a, b). These pro-apoptotic effects on M2 TAMs were abolished upon WISP-1 immunodepletion, reinforcing its essential role.

Correlation analyses demonstrated that TAM density (CD11b^+^) and the proportion of M2 TAMs (CD206^+^/CD11b^+^) positively correlated with both tumor volume and the number of proliferating tumor cells (Ki67^+^/CD326^+^) 24, while showing an inverse correlation with tumor cell apoptosis (cleaved caspase-3^+^/CD326^+^) (Supplementary Fig. S18a, b). Furthermore, Pearson's correlation analysis revealed that WISP-1 levels in the CM were inversely correlated with both TAM density and the proportion of M2 TAMs (CD206^+^/CD11b^+^), while positively correlated with the proportion of M1 TAMs (iNOS^+^/CD11b^+^) (Supplementary Fig. S18c). These findings suggest that higher WISP-1 levels are associated with a reduction in immunosuppressive TAM populations and enhanced polarization toward an anti-tumor M1-like phenotype.

Collectively, these data suggest that ApoSQ-CAF CM injection robustly reduced TAM density and attenuated the M2 TAM fraction. This reduction was associated with an upregulation of apoptosis in M2 TAMs, leading to the suppression of lung cancer cell growth in primary tumors through the mediation of WISP-1.

### ApoSQ-CAF CM induces *in vivo* reprogramming of TAMs from an M2 to an M1-like phenotype via WISP-1 signaling

**Figure 6:**
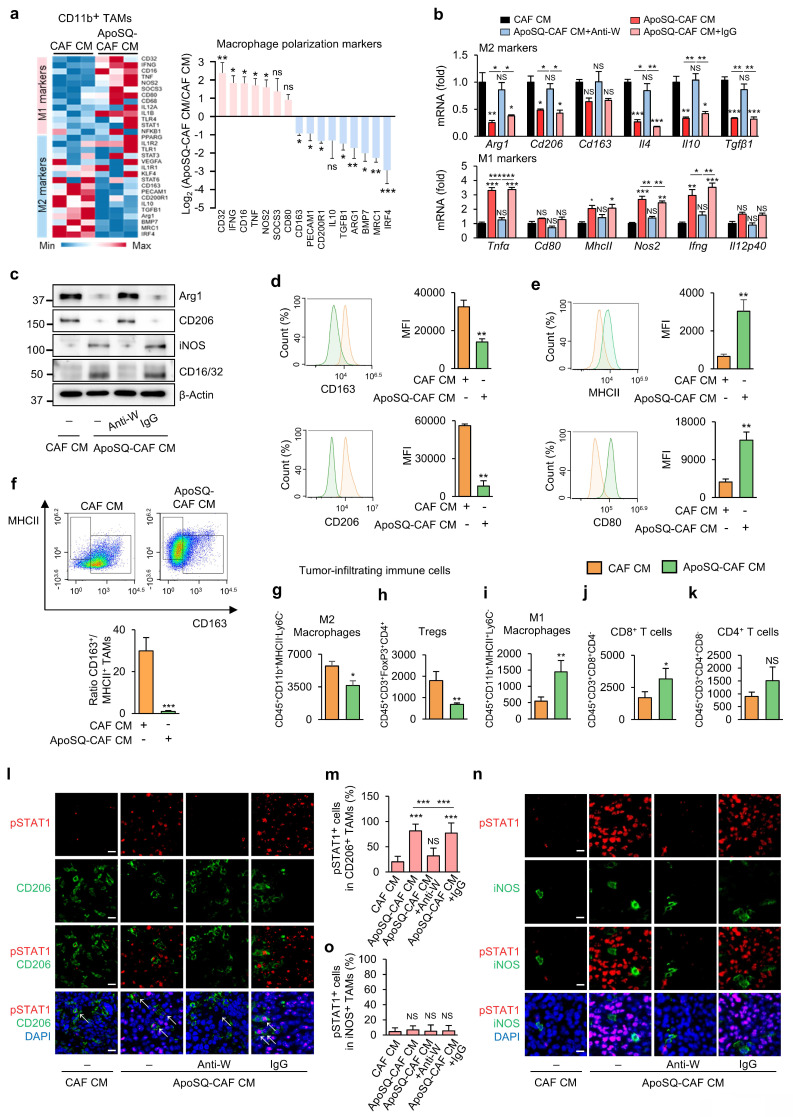
** ApoSQ-CAF CM promotes M2-to-M1 TAM reprogramming and activates STAT1 in M2 TAMs via WISP-1.** The experimental design was described in Fig. 5a. (**a**) Heatmap showing differentially expressed genes encoding M1 and M2 marker-related molecules in isolated CD11b^+^ TAMs from primary tumors (left). Red: high expression; blue: low expression. Relative expression of selected genes from PCR array profiling of macrophage polarization markers (right). Log2 fold-change values (ApoSQ-CAF CM vs. CAF CM). (**b**) qRT-PCR analysis of relative mRNA levels of M2 markers (*Arg1*, *Cd206*, *Cd163*, *Il4*, *Il10*, *Tgfβ1*), and M1 markers (*Tnfα*, *Cd80*, *MhcII*, *Nos2*, *Ifng*, and *Il12p40*) in isolated CD11b^+^ TAMs from primary tumors. NS: not significant; *P < 0.05, **P < 0.01, ***P < 0.001, Analysis of variance with Tukey's post hoc test. (**c**) Immunoblot analysis of Arg1, CD206, iNOS, and CD16/32 in isolated CD11b^+^ TAMs from primary tumors. (**d**,** e**) Flow cytometry analysis of the population of M1 TAMs (MHCII^+^ and CD80^+^) and M2 TAMs (CD163^+^ and CD206^+^) in CD11b^+^ TAMs from primary tumors. Mean fluorescence intensity (MFI) values (*right*). (**f**) *Upper:* Representative flow cytometry plots in CD11b^+^ TAMs. *Lower*: TAM ratio (CD163^+^/MHCII^+^ TAMs). (**g-k**) Flow cytometry analysis of the population of M2 macrophages (**g**), Tregs (**h**), M1 macrophages (**i**), CD8^+^ T cells (**j**), and CD4^+^ T cells (**k**). Tumor-infiltrating immune cells were stained with antibodies against CD45, CD11b, CD3, CD4, CD8, FoxP3, MHCII, and Ly6C. Absolute number of each cell type was counted using flow cytometry. (**a**, **d-k**) NS, not significant; *P < 0.05, **P < 0.01, ***P < 0.001, two-tailed Student's *t*-test. (**a**-**k**) The data are from three replicates per condition, with cells pooled from three mice per replicate. (**l, n**) Representative confocal images of primary tumor sections stained with an anti-phosphorylated STAT1 (red), anti-CD206 antibody (green), anti-iNOS antibody (green), and DAPI (blue). Original magnification: ×40. Scale bars = 100 μm. (**m**, **o**) Quantification of phosphorylated STAT1^+^ cells among CD206^+^ cells and iNOS^+^ cells. NS, not significant; ***P < 0.001, Analysis of variance with Tukey's post hoc test. The data are from one experiment representative of three independent experiments with similar results (**a**, **d** and** e *left***; **c**, **l**, **n**; **f *upper***) or from three independent experiments (mean ± standard error: **a**, **d** and** e *right***; **b, g-k**,** m**, **o**; **f *lower***).

Reprogramming TAMs toward an anti-tumor M1 phenotype offers a promising therapeutic strategy 10. To investigate whether ApoSQ-CAF CM injection induces TAM reprogramming *in vivo*, we analyzed the expression of M1- and M2-associated markers in CD11b^+^ TAMs isolated from primary tumors using a targeted RT-qPCR array. Among 30 marker genes, nine M2-associated genes— *Irf4*, *Mrc1, Bmp7, Arg1, Tgfb1, Il10, Cd200r1, Pecam1,* and* Cd163—* were downregulated by more than 2-fold in the ApoSQ-CAF CM group compared to the CAF CM group (Fig. 6a). Conversely, seven M1-associated genes—*Cd32*, *Ifng*, *Cd16*, *Tnf*, *Nos2*, *Socs3*, and *Cd80*—were upregulated by more than 2-fold in the ApoSQ-CAF CM group. Further qRT-PCR analysis confirmed that ApoSQ-CAF CM significantly reduced expression of M2-specific markers and cytokines (*Arg1, CD206, CD163, Il-4, Il-10, and Tgfβ1*), while increasing M1-associated markers and cytokines (*Tnfα, Cd80, MhcII, Nos2, Ifng, and Il12p40*) compared with control CAF CM (Fig. 6b). Consistent changes at the protein level were also observed: expression of M2 markers Arg1 and CD206 was reduced, while M1 markers iNOS and CD16/CD32 were increased following ApoSQ-CAF CM injection (Fig. 6c). Importantly, these effects were reversed by WISP-1 immunodepletion from ApoSQ-CAF CM, while CM containing IgG isotype control had no effect, highlighting WISP-1's essential role in mediating TAM reprogramming.

Flow cytometric analysis of isolated CD11b^+^ TAMs corroborated these findings, showing a reduction in M2 TAMs (CD163^+^/CD11b^+^ or CD206^+^/CD11b^+^) and an increase in M1-like TAMs (MHCII^+^/CD11b^+^ or CD80^+^/CD11b^+^) in the ApoSQ-CAF CM group compared with the CAF CM group (Fig. 6d, e). Accordingly, the M2/M1 ratio (CD163^+^/MHCII^+^) was markedly decreased (Fig. 6f).

In addition, analysis of total tumor-infiltrating immune cells, gated by CD45^+^ expression (Supplementary Fig. S19a, b), further revealed that ApoSQ-CAF CM treatment significantly decreased the population of immunosuppressive M2 TAMs and regulatory T cells (Tregs) (Fig. 6g, h), while increasing immune-stimulatory M1 TAMs and CD8^+^ T cells (Fig. 6i, j), compared to CAF CM. Although CD4^+^ T cells showed a trend toward increased infiltration, this change was not statistically significant (Fig. 6k). Pearson's correlation analyses further supported these observations: the proportion of M2 TAMs (CD206^+^/CD11b^+^) was negatively correlated with CD8^+^ T cell density and positively correlated with FoxP3^+^ Tregs (Supplementary Fig. S20a). In contrast, the proportion of M1 TAMs (CD86^+^/CD11b^+^) showed a positive correlation with CD8^+^ T cells and a negative correlation with FoxP3^+^ Tregs (Supplementary Fig. S20b). Additionally, WISP-1 levels in the CM were positively correlated with CD8^+^ T cell infiltration and negatively correlated with FoxP3^+^ Treg abundance (Supplementary Fig. S20c). No significant correlations were observed between CD4^+^ T cells and M1/M2 TAM proportions or WISP-1 levels. Collectively, these findings indicate that ApoSQ-CAF CM reprograms TAMs from an immunosuppressive M2 phenotype to an immune-stimulatory M1-like phenotype, thereby remodeling the tumor immune microenvironment and alleviating immunosuppression within the TME.

Our *in vitro* data indicated that ApoSQ-CAF CM suppresses M2 macrophages via WISP-1-dependant STAT1 signaling. To validate this mechanism *in vivo*, we examined whether ApoSQ-CAF CM enhances STAT1 phosphorylation preferentially in M2 TAMs. Immunofluorescent analysis of primary tumor tissues revealed a marked increase in phosphorylated STAT1 in M2 TAMs (pSTAT1^+^/CD206^+^) following injection of ApoSQ-CAF CM (Fig. 6l, m). This effect was abolished when WISP-1 was immunodepleted from the CM prior to injection. Notably, ApoSQ-CAF CM did not alter STAT1 phosphorylation in M1 TAMs (pSTAT1^+^/iNOS^+^) (Fig. 6n, o). Correlation analyses further demonstrated that phosphorylated STAT1^+^ M2 TAMs (pSTAT1^+^/CD206^+^) was positively correlated with both WISP-1 levels in the CM and apoptosis of M2 TAMs (cleaved caspase-3^+^/CD206^+^)*,* while negatively correlated with the proportion of CD206^+^ M2 TAMs (CD206^+^/CD11b^+^) (Supplementary Fig. S20d). These findings suggest that WISP-1-induced STAT1 activation is associated with enhanced apoptosis and reduced polarization of M2 TAMs. Collectively, these results support a model in which ApoSQ-CAF CM promotes TAM reprogramming from an immunosuppressive M2 phenotype to an immune-stimulatory M1-like state through WISP-1-mediated STAT1 signaling.

### rWISP-1 replicates the modulating effects of ApoSQ-CAF CM on the survival and reprogramming of TAMs *in vivo*

**Figure 7:**
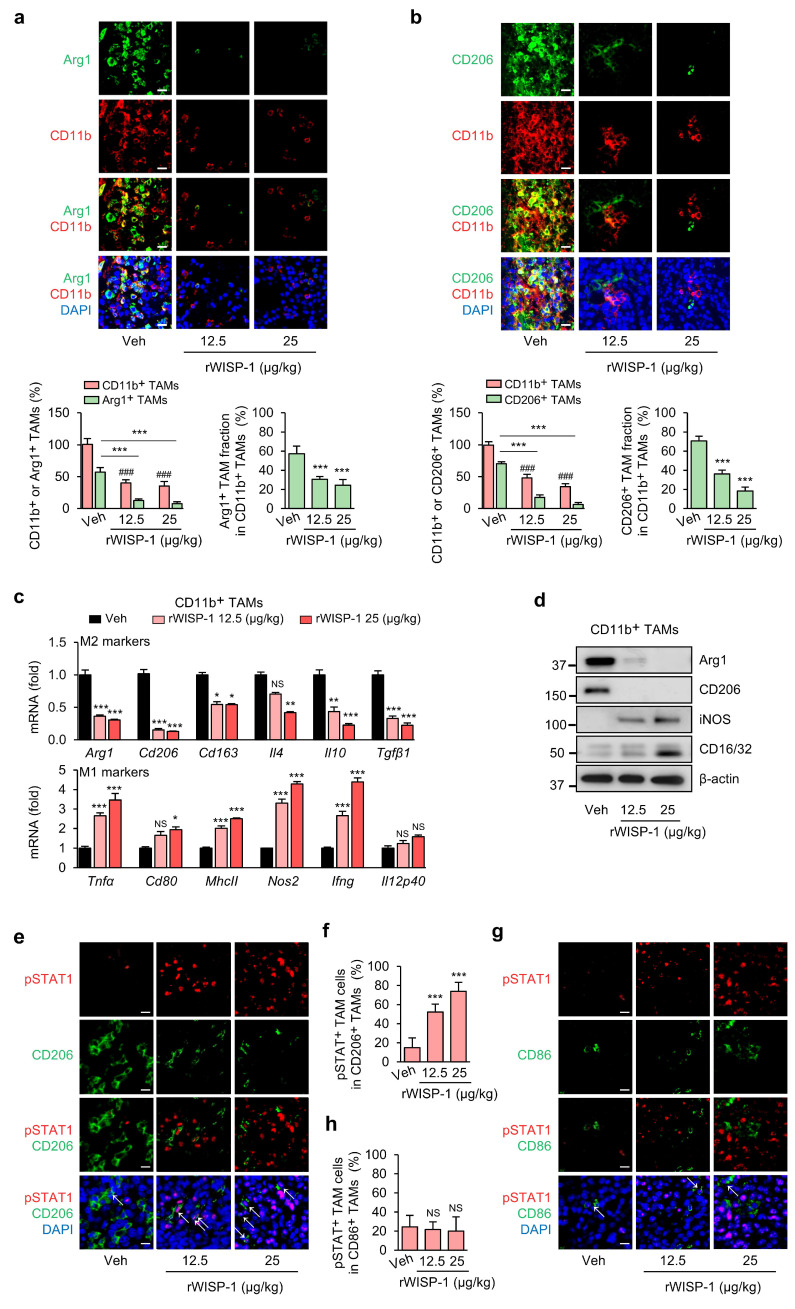
** Administration of rWISP-1 reduces TAM density, decrease the M2 fraction and marker expression, and activates STAT1 in M2 TAMs.** The experimental design was described in Supplementary Fig. S21a. Where indicated, rWISP-1 (12.5 and 25 μg/kg) was administered intratumorally three times a week for 6 weeks starting 2 days after subcutaneous implantation of 344SQ cells into syngeneic (129/Sν) mice (n = 6 mice per group). Mice were necropsied 6 weeks later. (**a**, **b**)* Upper*: Immunofluorescent staining of primary tumor sections showing M2 TAM Markers Arg1 (green) and CD206 (green), along with the pan-macrophage marker CD11b (red). Original magnification: ×40. Scale bars = 100 μm. *Lower*: Quantitation of Arg1^+^ and CD206^+^ TAM (M2) density (*left*) and the fraction of M2 TAMs (*right*) in primary tumors. The fraction of M2 TAMs were determined by the percentage of M2 TAMs within CD11b^+^ TAMs. (**c**) qRT-PCR analysis of relative mRNA levels of M2 markers (*Arg1*, *CD206*, *CD163*, *IL-4*, *IL-10*, *TGF-β1*), and M1 markers (*TNFα*, *CD80*, *MhcII*, *NOS2, Ifng,* and *IL-12 p40*) in isolated CD11b^+^ TAMs from primary tumors. (**d**) Immunoblot analysis of Arg1, CD206, iNOS, and CD16/32 in isolated CD11b^+^ TAMs from primary tumors. (**e, g**) Representative confocal images of primary tumor sections stained with an anti-phosphorylated STAT1 (red), anti-CD206 antibody (green), anti-CD86 antibody (green), and DAPI (blue). Original magnification: ×40. Scale bars = 100 μm. (**f**,** h**) Quantification of phosphorylated STAT1^+^ cells among CD206^+^ cells and CD86^+^ cells. NS, not significant; *P < 0.05, **P < 0.01, ***P < 0.001 compared to Vehicle or as indicated; ^###^*P* < 0.001 compared to Vehicle, Analysis of variance with Tukey's post hoc test. The data are from one experiment representative of three independent experiments with similar results (**a** and** b *upper***;** d**,** e**,** g**). The data are represented as the means ± standard errors from three mice per group (**a** and** b *lower***; **c**, **f**, **h**).

To further validate that the effects of ApoSQ-CAF CM on TAM survival and reprogramming are mediated by WISP-1, rWISP-1 (12.5 and 25 μg/kg) was intratumorally administered three times per week, starting two days after subcutaneous injection of 344SQ cells into syngeneic (129/Sv) mice (Supplementary Fig. S21a). Previously, we demonstrated that rWISP-1 can fully recapitulates the anti-tumor growth and antimetastatic effects of ApoSQ-CAF CM in mice models 22, 23. In the present study, consistent with the effects of ApoSQ-CAF CM, rWISP-1 effectively reduced total TAM density and the proportion of the M2 TAMs, while increasing the proportion of M1 TAMs (Fig. 7a, b and Supplementary Fig. S21b, c). Moreover, rWISP-1 induced apoptosis of M2 TAMs without effecting M1 TAMs (Supplementary Fig. S21d-g) and promoted their reprogramming toward an M1-like phenotype (Fig. 7c, d). Immunofluorescent analysis of primary tumor tissues further revealed a dose-dependent increase in phosphorylated STAT1 within M2 TAMs (pSTAT1^+^/CD206^+^) following rWISP-1 treatment in a dose-dependent manner, whereas no significant change was observed in M1 TAMs (pSTAT1^+^/CD86^+^) (Fig. 7e-h).

Our previous study demonstrated that WISP-1-STAT1 signaling contributes to the inhibitory effects of ApoSQ-CAF CM on tumor growth and lung metastasis, as shown using the STAT1 inhibitor fludarabine 23. In the present study, we further examined whether this signaling axis mediates the regulation of TAM fate—encompassing survival, apoptosis, and phenotypic transition—within the TME. Immunofluorescent analysis showed that treatment with fludarabine (10 mg/kg) attenuated rWISP-1-induced STAT1 phosphorylation in M2 TAMs (pSTAT1^+^/CD206^+^) (Supplementary Fig. S22a-e) and reversed the effects of rWISP-1 by restoring total TAM density and the proportion of the M2 TAMs (Supplementary Fig. S22f, g), while reducing the proportion of M1 TAMs (Supplementary Fig. S23a, b) and the level of M2 TAM apoptosis (Supplementary Fig. S23c-f). This reversal was accompanied by changes in immune cell composition within the primary tumor, including a decrease in CD8^+^ T cells and an increase in regulatory T cells (Supplementary Fig. S24a-c).

## Discussion

CAFs and TAMs are pivotal stromal and immune components within the TME, where they dynamically interact to promote tumor progression, immune suppression, and resistance to therapy. In our previous work, we demonstrated that CAFs reprogrammed by apoptotic cancer cells suppress tumor growth and metastasis 22, 23. However, the mechanistic basis through which these reprogrammed CAFs influence TAMs to reshape the immune landscape remained unclear. In this study, we reveal that CAFs exposed to apoptotic cancer cells (ApoSQ-CAFs) secrete factors that selectively impair M2-like TAMs by reducing their survival, inducing apoptosis, and promoting reprogramming toward an M1-like phenotype. These effects are mediated through a paracrine mechanism involving WISP-1, which activates the integrin α5β3-STAT1 signaling axis in M2 macrophages. These findings uncover a previously unappreciated immunoregulatory mechanism by which apoptotic cancer cell-CAF interactions can reshape the immune landscape of the TME to suppress tumor progression.

TAMs represent one of the most abundant immune cell populations in lung cancer, displaying functional plasticity between tumor-promoting M2-like and tumor-suppressing M1-like states 31. This plasticity is orchestrated by a complex network of cytokines, chemokines, and stromal cell-derived signals 9. Given their central role in modulating immune surveillance, angiogenesis, and therapeutic resistance, TAMs are emerging as important targets in non-small cell lung cancer (NSCLC) 10, 32. Our findings underscore the therapeutic potential of modulating TAM fate through CAF-derived WISP-1 as an immunological reprogramming strategy.

Using in vitro models, we demonstrated that CM from CAFs exposed to apoptotic 344SQ or A549 cells (ApoSQ-CAF CM, ApoA-CAF CM, and ApoA-*h*CAF CM) selectively reduced viability and induced apoptosis in M2-polarized THP-1 cells or BMDMs, without affecting M0 and M1 macrophages. This selective activity was absent in CM from untreated CAFs or from CAFs exposed to necrotic cells. The CM from CAFs exposed to apoptotic cancer cells also promoted a phenotypic shift of M2 macrophages toward M1-like phenotype, as evidenced by reduced expression of M2 markers and increased expression of M1 markers. These effects were consistent across human and murine systems, indicating translational relevance.

Our *in vivo* data support the *in vitro* findings. Intratumoral administration of ApoSQ-CAF CM markedly reduced overall TAM density in primary tumors, with a pronounced depletion of immunosuppressive M2 TAMs accompanied by increased apoptosis. This was paralleled by enhanced reprogramming of M2 TAMs into an M1-like phenotype, as confirmed by decreased M2/M1 ratios, downregulation of M2-associated genes, and upregulation of M1 markers in TAMs. Importantly, these immunological shifts were associated with reduced regulatory T cell infiltration and increased recruitment of CD8^+^ cytotoxic T cells, suggesting that ApoSQ-CAF CM reshapes the TME to favor anti-tumor immunity and attenuate lung cancer progression.

In our previous study, we demonstrated that UV-irradiated apoptotic lung cancer cells induce WISP-1 expression in CAFs through activation of the Notch1 signaling pathway. Specifically, apoptotic cancer cells display increased surface expression of the Notch ligand Delta-like 1 (DLL1), which interacts with Notch1 receptors on CAFs and subsequently drives transcriptional activation of *WISP-1* 22. This apoptotic cell-CAF communication axis promotes WISP-1 secretion, thereby suppressing lung cancer cell proliferation, migration, and invasion within the TME 22, 23. In the present study, we further identified WISP-1 as a key effector mediating the TAM-modulating activity of CM derived from apoptotic cancer cell-primed CAFs (ApoSQ-CAF CM and ApoA-CAF CM). Based on* in vitro* studies, both rWISP-1 and CM from CAFs or *h*CAFs exposed to apoptotic lung cancer cells induced STAT1 phosphorylation specifically in M2 macrophages, triggering apoptosis and phenotypic reprogramming. These effects were abolished by pharmacologic inhibition (fludarabine) or genetic knockdown of STAT1, confirming the necessity of STAT1 in this process. Similarly, neutralization of WISP-1 in CM abrogated its TAM-modulatory effects *in vivo*. Moreover, rWISP-1 injection recapitulated these changes in vivo, suppressing M2 TAMs, their reprogramming into M1-like macrophages, and the activation of anti-tumor immune responses 23. Conversely, *in vivo* fludarabine treatment reversed rWISP-1-induced CD8^+^ T cell infiltration and restored Treg accumulation, further confirming the pivotal role of the WISP-1-STAT1 axis in modulating the tumor immune landscape.

These results are consistent with previous reports that STAT1-deficient TAMs fail to elicit effective T-cell responses and lack critical effector functions, such as iNOS expression 38-40. Elevated STAT1 expression in macrophages correlates with improved patient survival and a more active tumor immune landscape, suggesting its potential as a predictive biomarker for immunotherapy responsiveness 40. Furthermore, manipulating macrophage polarization toward the M1 phenotype by enhancing STAT1 phosphorylation while suppressing STAT3 activity has also shown therapeutic promise in other malignancies, including glioblastoma 41.

Mechanistically, our data support that STAT1 activation is required for the pro-apoptotic effects elicited by CM from apoptotic cancer cell-primed CAFs and by rWISP-1 treatment. STAT1 knockdown or pharmacological inhibition (fludarabine) abolished CM/rWISP-1-induced upregulation of Bax, cleaved caspase-3, and cleaved PARP, while restoring anti-apoptotic proteins such as Bcl-xL and Mcl-1. These findings align with the established function of STAT1 in apoptosis control—transcriptionally upregulating pro-apoptotic genes (e.g., *BAX*,* FAS*,* TNFSF10*) while repressing anti-apoptotic genes (e.g., *BCL2*, *BCL2L1*)—thereby providing a direct gene-regulatory basis for the observed reduction in M2 macrophage survival 42-45.

Although we did not directly assess STAT1 transcriptional targets in this study, prior research has delineated a STAT1-linked transcriptional network that orchestrates macrophage polarization. Upon activation, STAT1 promotes M1-associated transcription factors, such as IRF1 and IRF5, while antagonizing M2-promoting factors, including IRF4, STAT6 46-51. Notably, STAT1 also engages in complex, context-dependent crosstalk with NF-κB signaling: STAT1 can cooperate with NF-κB to amplify inflammatory/M1 programs 52, whereas acetylated STAT1 can interact with p65 (RelA) subunit to dampen NF-κB-dependent transcription 53.

WISP-1 is known to signal through specific integrin receptors in a cell-type-dependent manner 33, 34, 54, 55. While our previous studies identified integrin αvβ3 in lung cancer cells and αvβ5 in CAFs as WISP-1 receptors 22, 23, the present study reveals integrin α5β3 as the receptor mediating WISP-1 signaling in M2 macrophages. Engagement of α5β3 was established functionally—blocking antibodies or siRNA against α5 or β3 disrupted WISP-1-dependent STAT1 activation—and biochemically—CoIP confirmed physical binding of WISP-1 to α5β3 in M2 macrophages. In contrast, inhibition of αv or β5 did not affect these responses, indicating a dominant, receptor-specific role for α5β3 in this context. Consistent with its functional importance, previous studies reported that deletion of integrin β3 in myeloid cells led to increased M2 TAM accumulation, reduced CD8^+^ T cell infiltration, and enhanced tumor growth in murine models 56. Furthermore, integrin β3 signaling has been linked to activation of STAT1 and suppression of STAT6, thereby promoting M1 polarization while inhibiting M2 differentiation 56, 57. Together, these findings underscore integrin α5β3 as a key receptor that transduces WISP-1 signaling to activate STAT1 and reprogram M2 TAMs. Nevertheless, we acknowledge that additional or compensatory receptor systems may contribute to WISP-1 signaling. CCN family proteins, including WISP-1, are multifunctional matricellular proteins known to interact with various integrins in a cell type-dependent manner, such as α5β1, αvβ3, αvβ5, and α6β1 22, 23, 58-63. Moreover, recent studies have shown that WISP-1 can associate with heparan sulfate proteoglycans (HSPGs) and low-density lipoprotein receptor-related proteins (LRPs), which may serve as non-integrin co-receptors that modulate downstream signaling 64, 65. While these alternative interactions were not evident under our experimental conditions, we cannot exclude the possibility that WISP-1 engages distinct receptor complexes depending on the cellular context or microenvironmental cues. Further investigation using receptor-blocking assays and ligand-receptor mapping will be necessary to fully delineate the receptor repertoire governing WISP-1 signaling in macrophages within the TME.

Our single-cell and cohort analyses collectively support a stromal-myeloid axis in which fibroblast-derived WISP-1 promotes STAT1 activation in M2-like TAMs within the NSCLC microenvironment. Strong correlations between CCN4 and STAT1/pSTAT1(S727) in CPTAC-LUAD and TCGA-LUAD suggest that microenvironmental WISP-1 contributes to STAT1 pathway engagement in patients 23. Reanalysis of the Zuani et al. dataset 35 confirmed that WISP-1 is selectively expressed by activated adventitial fibroblasts, whereas STAT1 is enriched in STAB1^+^ M2-like macrophages. These findings align with our experimental data showing that WISP-1 reprograms M2 macrophages toward an M1-like phenotype through STAT1 signaling. Supporting clinical relevance, two independent cohorts (n=307) demonstrated that low CD68^+^/pSTAT1^+^ TAMs and a reduced M1/M2 ratio predict poor prognosis 66. In addition, STAT1 expression in macrophages identified patients with improved survival and an intact tumor immune system, who may benefit from immunotherapy 40. Together, these results propose that stromal WISP-1 acts as a paracrine inducer of STAT1 activity in TAMs, thereby reshaping macrophage polarization toward an anti-tumor state. This WISP-1-STAT1 axis may serve as both a mechanistic biomarker and a potential therapeutic target for improving immune responsiveness in NSCLC.

In summary, this study uncovers a previously unrecognized mechanism by which apoptotic cancer cell-educated CAFs actively modulate the tumor immune microenvironment. Specifically, we demonstrate that interactions between CAFs and apoptotic cancer cells elicit anti-survival and pro-apoptotic effects in M2 TAMs, driving their reprogramming toward an M1-like phenotype via the WISP-1-integrin α5β3-STAT1 signaling axis. This signaling cascade not only disrupts the immunosuppressive macrophage compartment but also promotes CD8^+^ T cell recruitment and suppresses regulatory T cell accumulation, thereby reshaping the TME into a more immunostimulatory and tumor-restrictive state. Therapeutically, targeting the WISP-1-integrin α5β3-STAT1 pathway offers a promising strategy to reprogram immunosuppressive TAMs, potentiate anti-tumor immunity, and improve the efficacy of immunotherapies in lung cancer and potentially other solid malignancies.

## Supplementary Material

Supplementary figures and tables.
