# Supplementary material for: Apoptotic Cancer Cell-Primed Cancer-Associated Fibroblasts Suppress Immunosuppressive Macrophages via WISP-1-Integrin α5β3-STAT1 Signaling in Lung Cancer
Source: Int J Biol Sci. 2026 Jan 1;22(2):995–1015. doi: 10.7150/ijbs.124282 (PMC12781172; doi:10.7150/ijbs.124282)
Supplement: Supplementary file 1 — Supplementary figures and tables. [file ijbsv22p0995s1.pdf]

Supplementary Information

Apoptotic Cancer Cell–Primed Cancer-Associated Fibroblasts Suppress Immunosuppressive Macrophages via WISP-1–Integrin α5β3–STAT1 Signaling in Lung Cancer

Kyungwon Yang, Kiyoon Kim, Hee Ja Kim, Jeesoo Chae, Ye-Ji Lee, Shinyoung Kim, Young-Ho Ahn, Jihee Lee Kang

Supplementary Figure 1

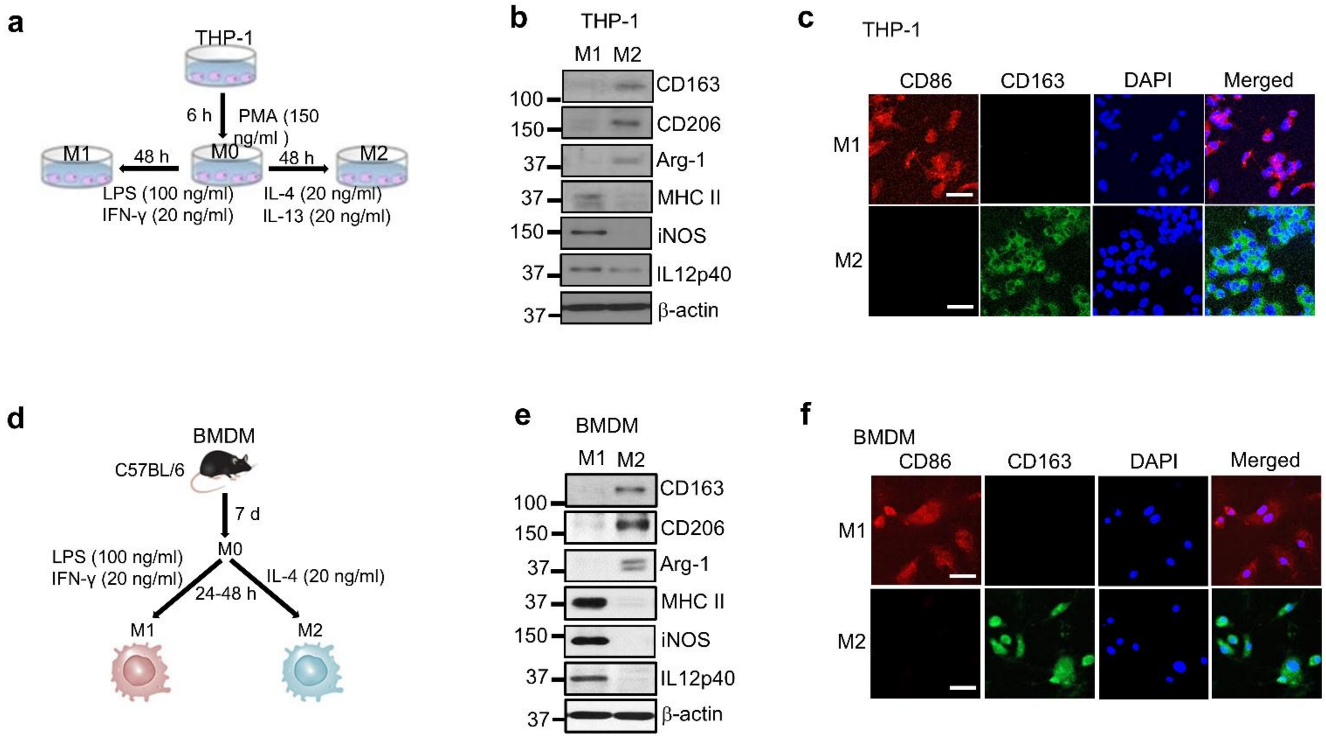

Supplementary Fig. S1. Assessment of macrophage polarization and effects of ApoSQ-CAF CM on apoptosis. (**a**, **d**) Schemes of the preparation and polarization of M1- and M2-type macrophages derived from THP-1 cells and mouse bone marrow-derived macrophages (BMDMs). (**b**, **e**) Immunoblot analysis of the M1 and M2 macrophage marker expression in M1 (M1) or M2 macrophages (M2) derived from THP-1 cells and BMDMs. (**c**, **f**) Immunofluorescence staining for the M1 marker CD86 and M2 marker CD163 in M1 and M2 macrophages polarized from THP-1 cells and BMDMs. The imaging medium was VECTASHIELD fluorescence mounting medium containing DAPI. Original magnification: ×400. Scale bars = 20 μm. Data are from one experiment representative of three independent experiments with similar results (**b**, **c**, **e**, **f**).

Supplementary Figure 2

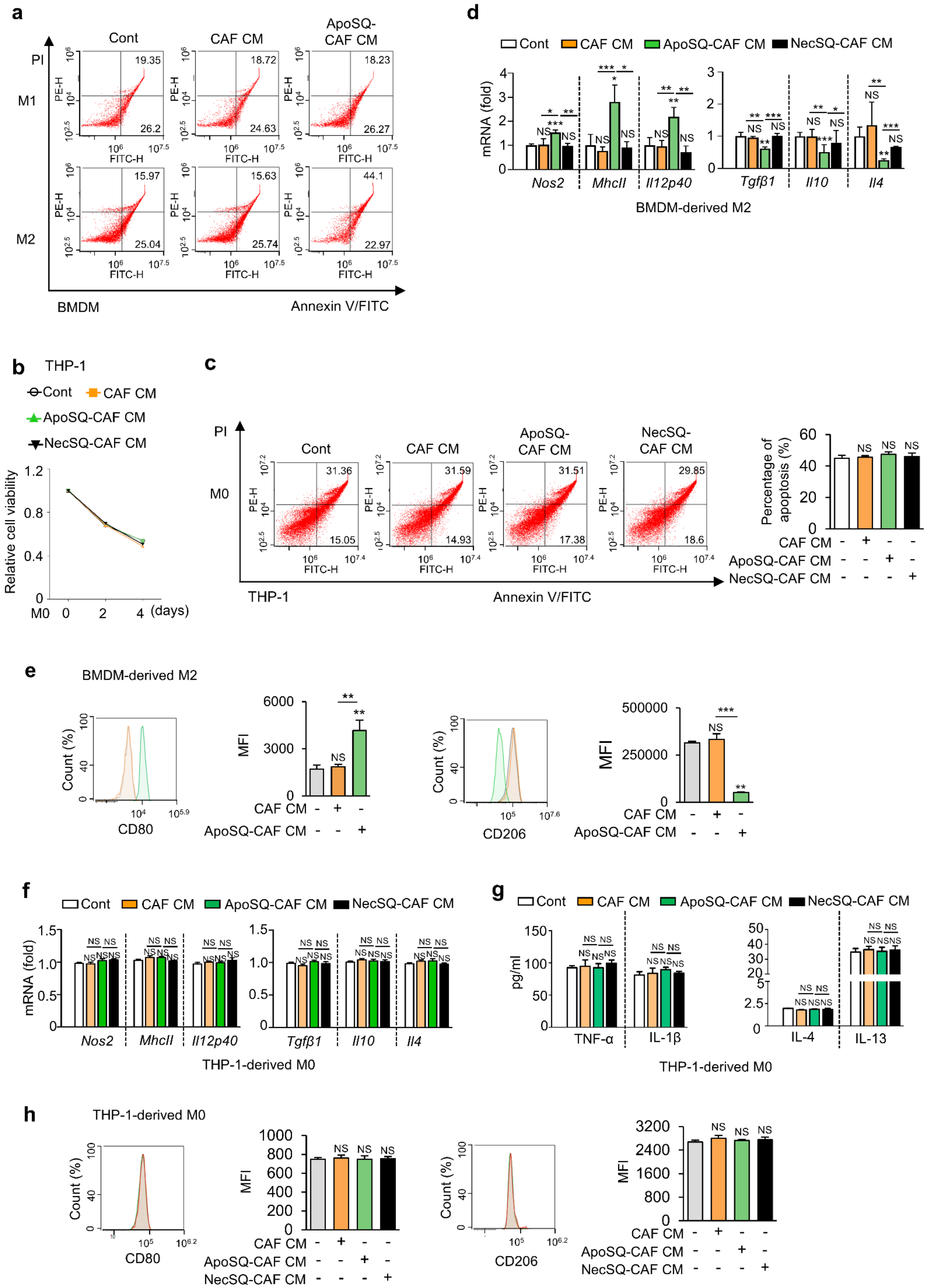

Supplementary Fig. S2. Effects of ApoSQ-CAF CM on apoptosis and reprogramming of macrophages. (**a**, **c**) Apoptosis of THP-1-derived M0 (**c**) and BMDM-derived M1 and M2 macrophages (**a**) was quantified as the sum of the percentages of early and late stages of apoptosis. Flow cytometry analysis after Annexin V−FICT/PI dual staining was employed to evaluate apoptosis. (**b**) Cell viability assay of M0 macrophages (M0) derived from THP-1 cells. (**d**, **f**) qRT-PCR analysis of relative mRNA levels of M1 (*Nos2*, *MhcII*, and *Il12p40*) and M2 (*Tgfβ1*, *Il10*, and *Il4*) markers in THP-1-derived M0 (**f**) and BMDM-derived M2 macrophages (**d**). (**e**, **h**) Flow cytometry analysis of the population of $CD80^+$ and $CD206^+$ macrophages among THP-1-derived M0 (**h**) and BMDM-derived M2 macrophages (**e**). Mean fluorescence intensity (MFI) values (*right*). (**g**) ELISA of TNF-α, IL-1β, IL-4, and IL-13 in culture media of M0 macrophages derived from THP-1 cells. (**a-h**) CAFs were exposed to apoptotic 344SQ cells (ApoSQ) or necrotic cancer cells (NecSQ) for 20 h. Conditioned medium from CAFs only (CAF CM), exposed to ApoSQ (ApoSQ-CAF CM) or NecSQ (NecSQ-CAF CM) was treated to M0, M1 or M2 macrophages for 2 or 3 days. NS, not significant; *$P < 0.05$, **$P < 0.01$, ***$P < 0.001$, two-tailed Student's *t*-test. Data are from one experiment representative of three independent experiments with similar results (**a**; **c, e**, and **h** ***left***) or from three independent experiments (mean ± standard error: **b**, **d**, **f**, **g**; **c, e**, and **h** ***right***).

Supplementary Figure 3

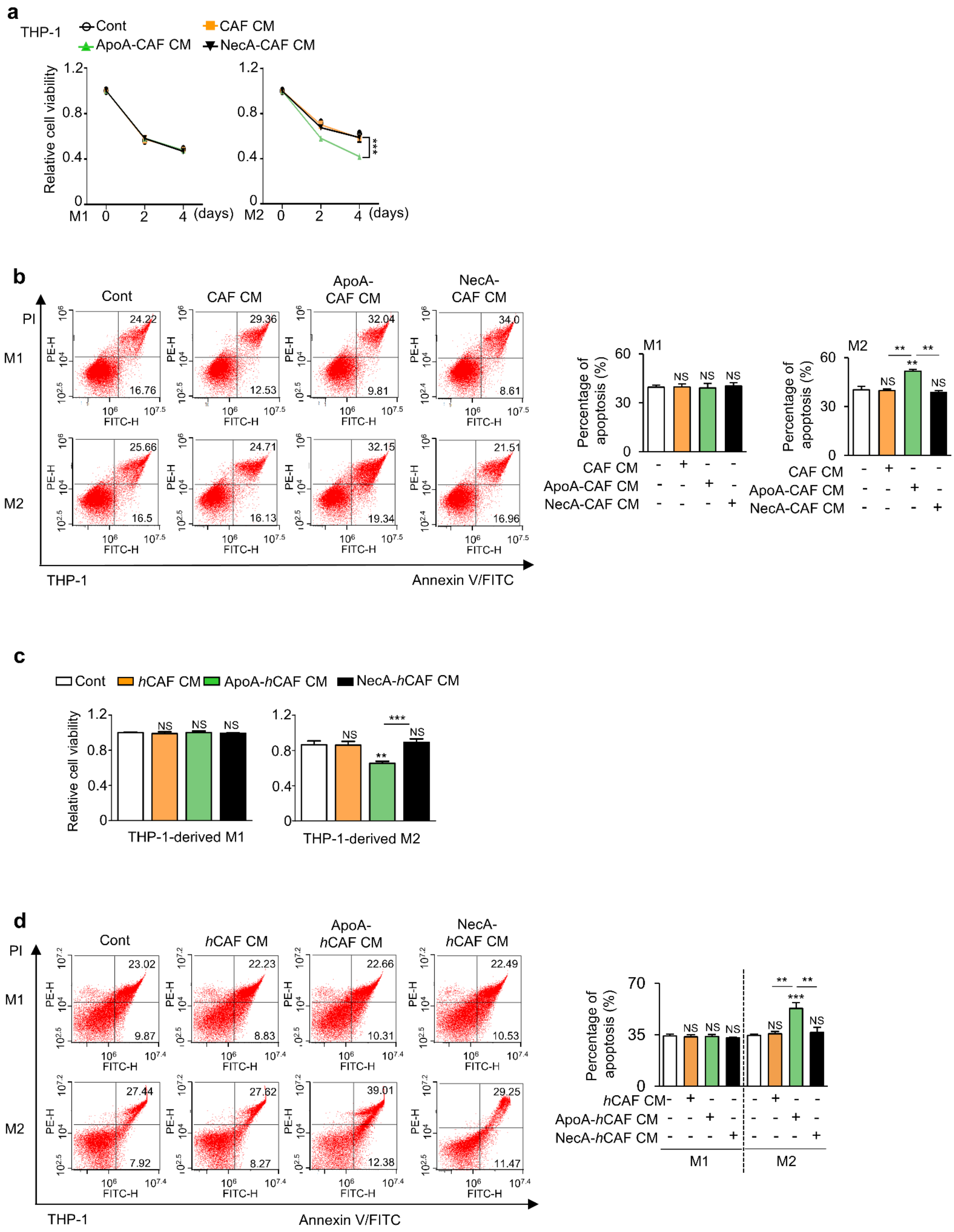

Supplementary Fig. S3. CM from CAFs exposed to apoptotic A549 cells reduces M2 macrophage survival and induces apoptosis. (**a**, **c**) Cell viability assay of M1 (M1) and M2 macrophages (M2) derived from THP-1 cells. (**b**, **d**) *Left:* Flow cytometry analysis after Annexin

V−FICT/PI dual staining was employed to evaluate the apoptosis of M1 and M2 macrophages polarized from THP-1 cells. *Right:* Apoptotic cells were quantified as the sum of the percentages of early and late stages of apoptosis. (**a**, **b**) CAFs were exposed to apoptotic A549 cells (ApoA) or necrotic A549 cells (NecA) for 20 h. Conditioned medium from CAFs only (CAF CM), exposed to ApoA (ApoA-CAF CM) or NecA (NecSQ-CAF CM) was treated to M1 or M2 macrophages for the indicated days or 3 days. (**c**, **d**) M1 and M2 macrophages were treated with CM from human CAFs only (*h*CAF CM), ApoA-*h*CAF CM, or NecA-*h*CAF CM for 3 days NS, not significant; $^{**}P < 0.01$, $^{***}P < 0.001$, two-tailed Student's *t*-test. Data are from one experiment representative of three independent experiments with similar results (**b** and **d** ***left***) or from three independent experiments (mean ± standard error: **a**, **c**; **b** and **d** ***right***).

Supplementary Figure 4

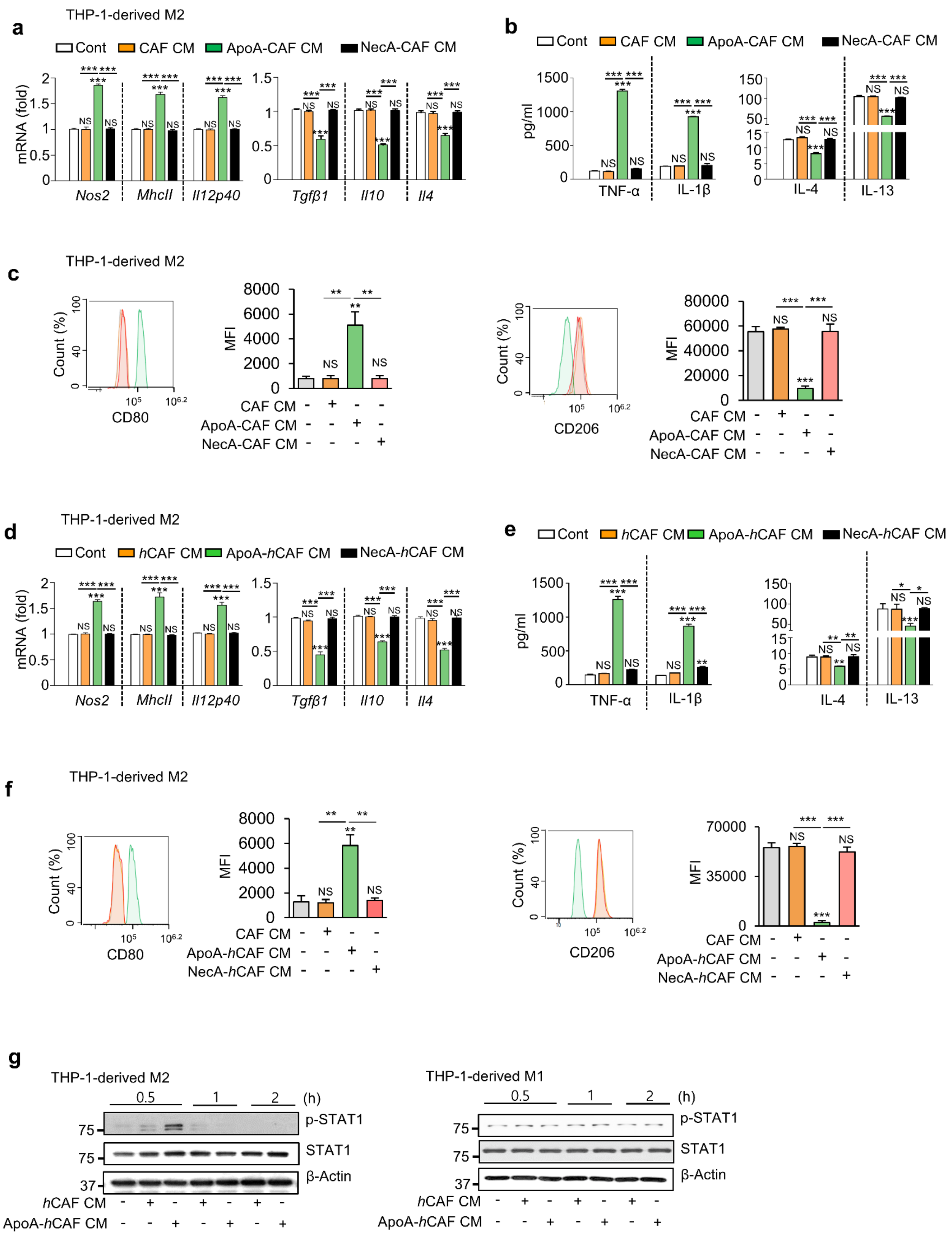

Supplementary Fig. S4. CM from CAFs exposed to apoptotic A549 cells promotes reprogramming toward an M1-like phenotype and activates STAT1 in M2 macrophages. (**a, d**) qRT-PCR analysis of relative mRNA levels of M1 (*Nos2*, *MhcII*, and *Il12p40*) and M2 (*Tgfβ1*,

Il10, and *Il4*) markers in M2 macrophages (M2) derived from THP-1 cells. (**b**, **e**) ELISA of TNF-α, IL-1β, IL-4, and IL-13 in culture media of M2 macrophages derived from THP-1 cells. (**c**, **f**) Flow cytometry analysis of the population of $CD80^+$ and $CD206^+$ macrophages among M2 macrophages derived from THP-1 cells. Mean fluorescence intensity (MFI) values (*right*). (**a-c**) CAF CM, ApoA-CAF CM, or NecA-CAF was treated to M2 macrophages for 2 or 3 days. (**d-f**) *h*CAF CM, ApoA-*h*CAF CM, or NecA-*h*CAF was treated to M2 macrophages for 2 or 3 days. (**g**) Immunoblot analysis of the indicated proteins in THP-1-derived M1 and M2 macrophages treated with *h*CAF CM or ApoSQ-*h*CAF CM for the indicated time. NS, not significant; **P* < 0.05, ***P* < 0.01, ****P* < 0.001, two-tailed Student's *t*-test. Data are from one experiment representative of three independent experiments with similar results (**c** and **f** ***left***; **g**) or from three independent experiments (mean ± standard error: **a**, **b**, **d**, **e**; **c** and **f** ***right***).

Supplementary Figure 5

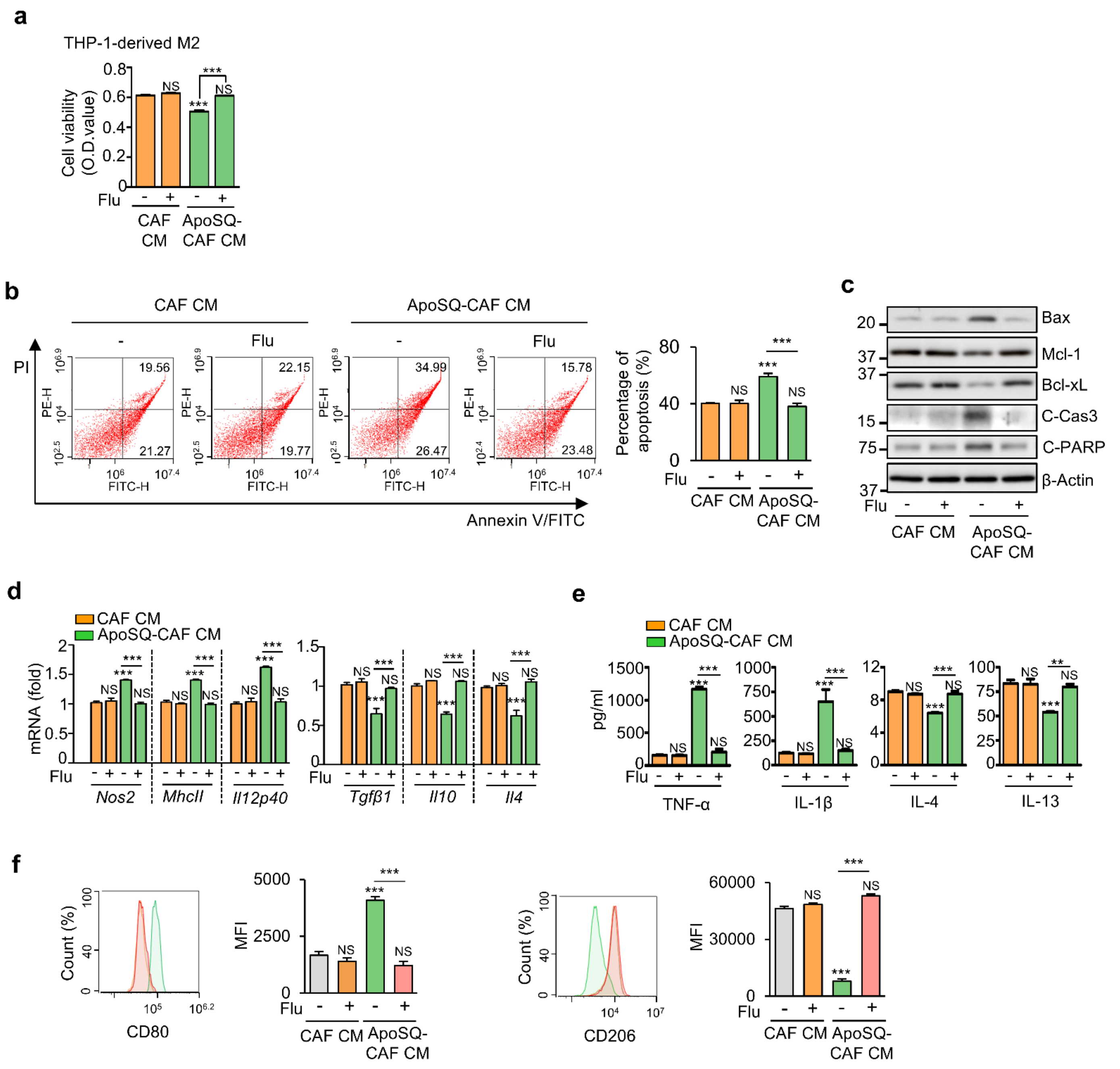

Supplementary Fig. S5. Fludarabine blocks STAT1-dependent reducing survival, promoting apoptosis and reprogramming of M2 Macrophages. (**a**) Cell viability assay of THP-1-derived M2 macrophages treated with CM for 3 days. (**b**) *Left:* Flow cytometry analysis after Annexin V−FICT/PI dual staining was employed to evaluate the apoptosis of M2 macrophages. *Right*: Apoptotic cells were quantified as the sum of the percentages of early and late stages of apoptosis 3 days after CM treatment. (**c**) Immunoblot analysis of the indicated proteins in M2 macrophage lysates 3 days after CM treatment. (**d**) qRT-PCR analysis of relative mRNA levels of M1 (*Nos2*, *MhcII*, and *Il12p40*) and M2 (*Tgfβ1*, *Il10*, and *Il4*) markers in M2 macrophages treated with CM for

3 days. (**e**) ELISA of TNF-α, IL-1β, IL-4, and IL-13 in the culture supernatants of THP-1-derived M2 macrophages treated with CM for 3 days. (**f**) Flow cytometry analysis of the population of $CD80^+$ and $CD206^+$ macrophages among M2 macrophages treated with CM for 2 or 3 days. Mean fluorescence intensity (MFI) values (*right*). (**a**-**f**) THP-1-derived M2 macrophages were pretreated with fludarabine (1 μM) 1 h before treatment with CAF CM or ApoSQ-CAF CM. NS: not significant; $^{**}P < 0.01$, $^{***}P < 0.001$, two-tailed Student's *t*-test. The data are from one experiment representative of three independent experiments with similar results (**b** and **f** **l*eft***; **c**) or from three independent experiments (mean ± standard error: **a**, **d**, **e**; **b** and **f** ***right***).

Supplementary Figure 6

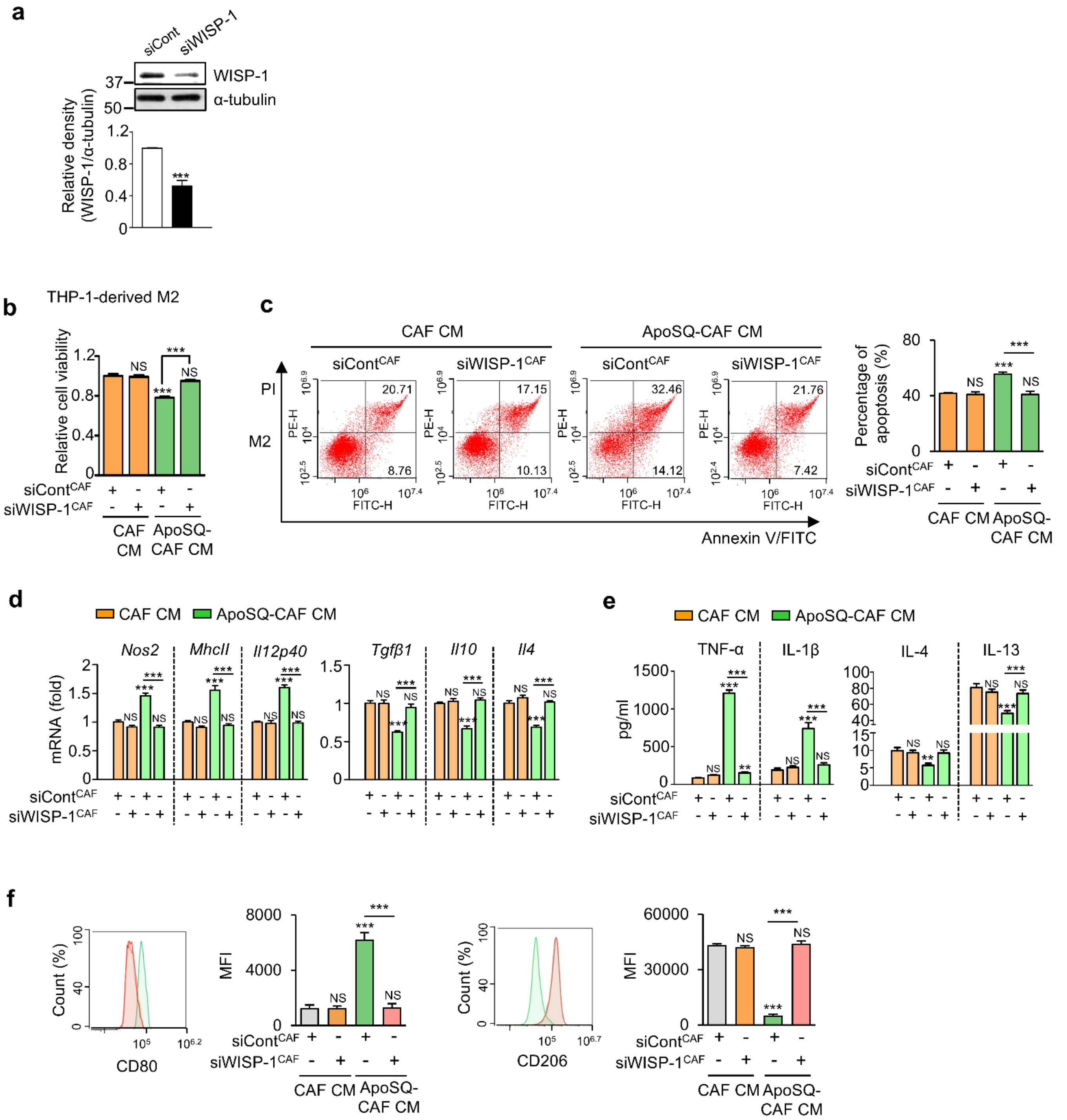

Supplementary Fig. S6. WISP-1 mediates the anti-survival, pro-apoptosis, and reprogramming effects of ApoSQ-CAF CM on M2 macrophages. (**a**) Immunoblot analysis of WISP-1 in CAFs transfected with control or WISP-1 siRNA (*upper*). Densitometric analysis of the relative WISP-1 abundance (*lower*). (**b**) Cell viability assay of THP-1-derived M2 macrophages (M2) treated with CM for 3 days. (**c**) *Left:* Flow cytometry analysis after Annexin V−FICT/PI dual staining was employed to evaluate the apoptosis of M2 macrophages treated with CM for 3 days. *Right*: Apoptotic cells were quantified as the sum of the percentages of early and late stages of

apoptosis. (**d**) qRT-PCR analysis of relative mRNA levels of M1 (*Nos2*, *MhcII*, and *Il12p40*) and M2 (*Tgfβ1*, *Il10*, and *Il4*) markers in THP-1-derived M2 macrophages treated with CM for 3 days. (**e**) ELISA of TNF-α, IL-1β, IL-4, and IL-13 in the culture supernatants of M2 macrophages treated with CM for 3 days. (**f**) Flow cytometry analysis of the population of $CD80^+$ and $CD206^+$ macrophages treated with CM for 2 or 3 days. Mean fluorescence intensity (MFI) values (*right*). (**b-f**) CAFs were transfected with control or WISP-1 siRNA before exposure to apoptotic 344SQ cells (ApoSQ) for 20 h. CAF CM or ApoSQ-CAF CM was treated to THP-1-derived M2 macrophages. NS: not significant; ***$P < 0.001$, two-tailed Student's *t*-test. The data are from one experiment representative of three independent experiments with similar results (**a** ***upper***; **c** and **f** ***left***) or from three independent experiments (mean ± standard error: **a** ***lower***; **b**, **d**, **e**; **c** and **f** ***right***).

Supplementary Figure 7

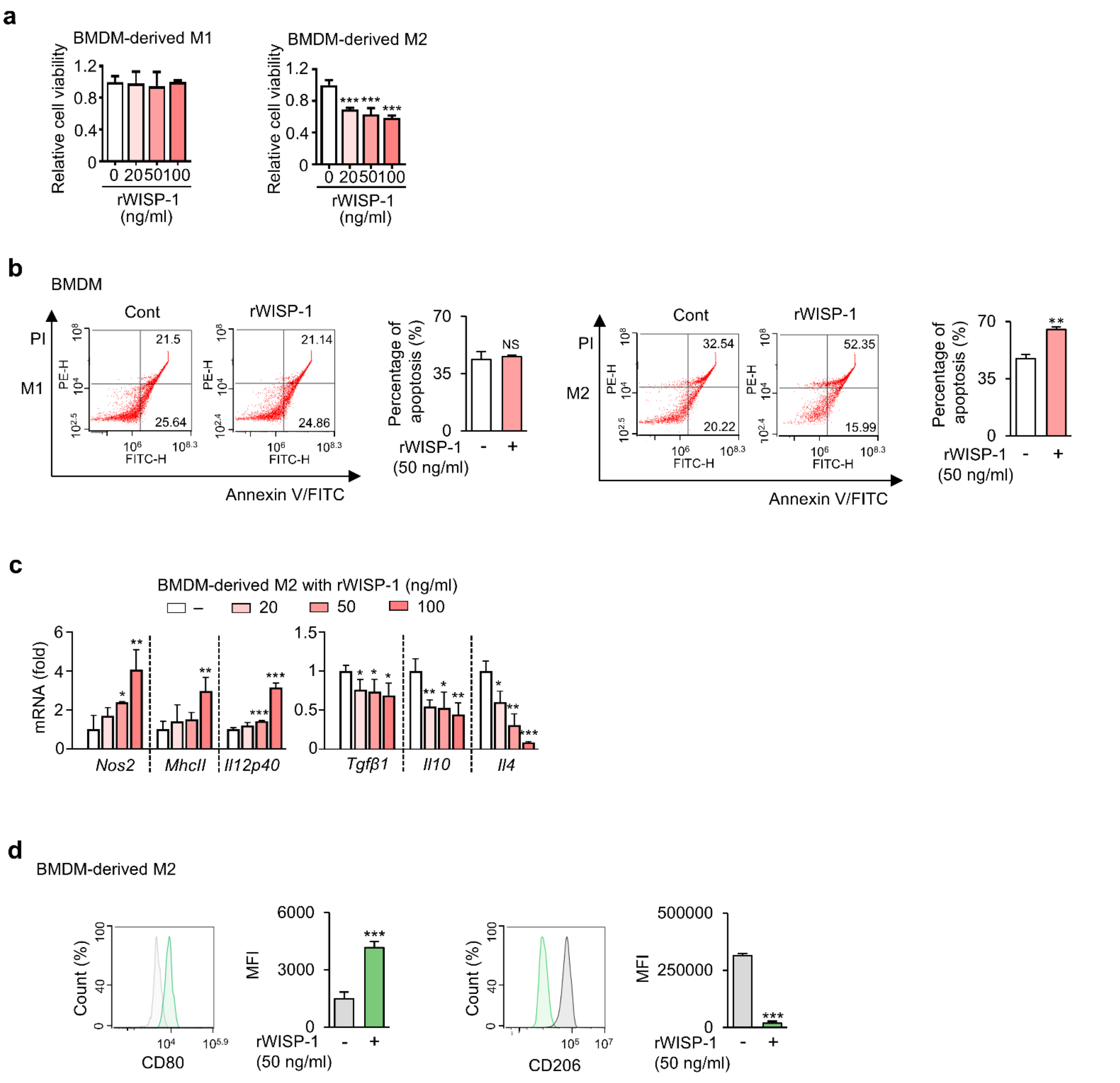

Supplementary Fig. S7. Recombinant WISP-1 reduces survival, induces apoptosis, and promotes reprogramming of BMDM-derived M2 macrophages toward an M1-like phenotype. (**a**) Cell viability assay of BMDM-derived M1 (M1) and M2 macrophages (M2) treated with 20-100 ng/ml mouse rWISP-1 (rWISP-1) for 3 days. (**b**) *Left:* Flow cytometry analysis after Annexin V−FICT/PI dual staining was employed to evaluate the apoptosis of BMDM-derived M1 and M2 macrophages treated with rWISP-1 (50 ng/ml) for 3 days. *Right*: Apoptotic cells were quantified as the sum of the percentages of early and late stages of apoptosis. (**c**) qRT-PCR analysis of relative mRNA levels of M1 (*Nos2*, *MhcII*, and *Il12p40*) and M2 (*Tgfβ1*, *Il10*, and *Il4*) markers in M2

macrophages treated with 20-100 ng/ml rWISP-1 for 3 days. (**d**) Flow cytometry analysis of the population of $CD80^+$ and $CD206^+$ macrophages among M2 macrophages (M2). Mean fluorescence intensity (MFI) values (*right*). BMDM-derived M2 macrophages were treated with 50 ng/ml rWISP-1 for 2 or 3 days. NS: not significant; *$P < 0.05$, **$P < 0.01$, ***$P < 0.001$, two-tailed Student's *t*-test. The data are from one experiment representative of three independent experiments with similar results (**b** and **d** ***left***) or from three independent experiments (mean ± standard error: **a**, **c**; **b** and **d** ***right***).

Supplementary Figure 8

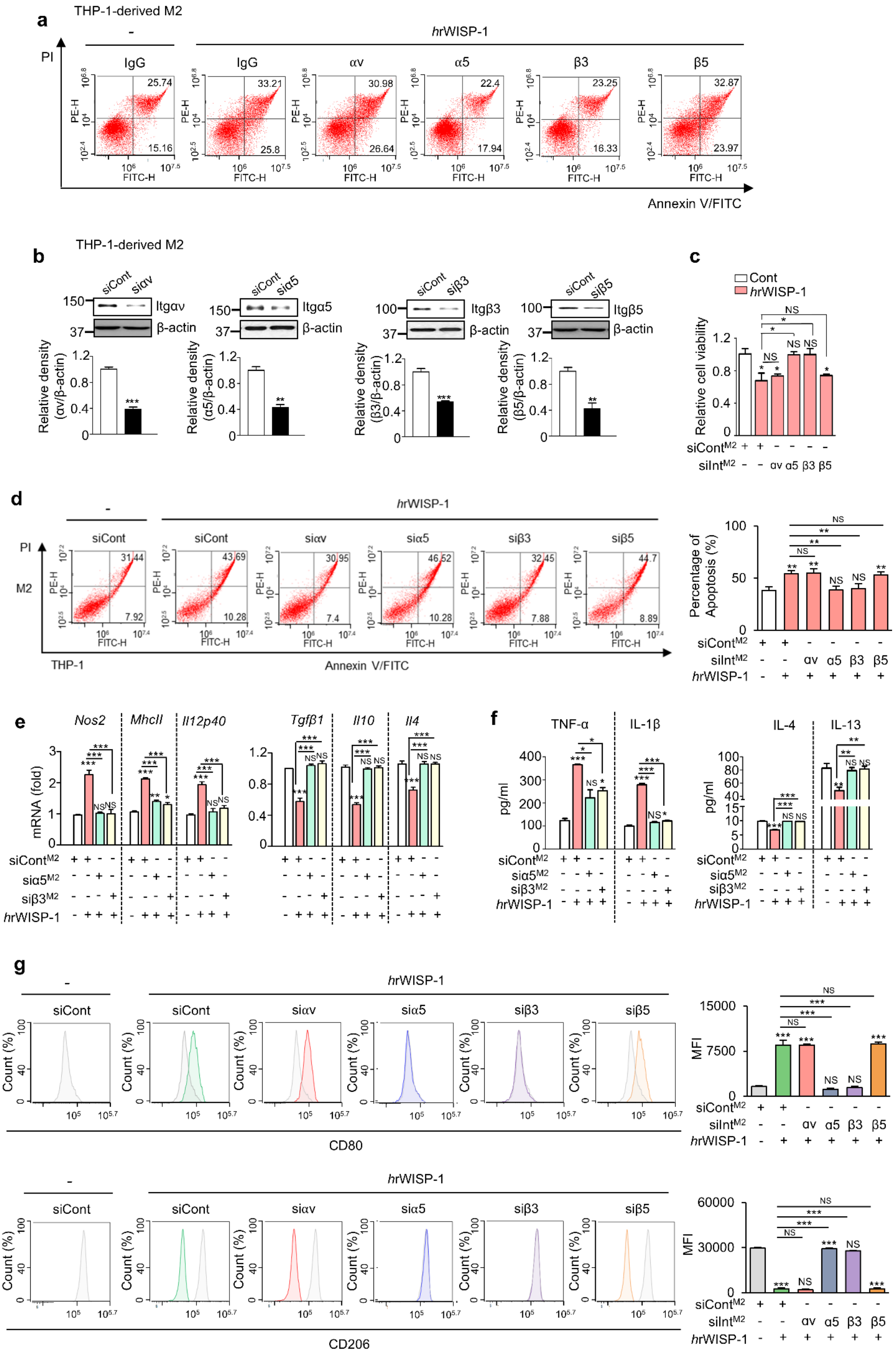

Supplementary Fig. S8. WISP-1-integrin α5β3 signaling mediates the anti-survival, pro-apoptotic, and reprogramming effects of the CM in M2 macrophages. (**a**) Flow cytometry analysis after Annexin V−FICT/PI dual staining was employed to evaluate the apoptosis of M2 macrophages treated with human rWISP-1 (*h*rWISP-1) for 3 days. THP-1-derived M2 macrophages were pretreated with an anti-integrin blocking antibody (3 μg/ml; anti-integrin αv, α5, β3 or β5) or corresponding IgG isotype control for 30 min prior to *h*rWISP-1 treatment (50 ng/ml) (**b**) Immunoblot analysis of the indicated proteins in THP-1-derived M2 macrophages transfected with control or siRNA of integrin αv, α5, β3, or β5 (*upper*). Densitometric analysis of the relative integrin αv, α5, β3, or β5 abundance (*lower*). (**c**) Cell viability assay of M2 macrophages (M2) treated with *h*rWISP-1 for 3 days. (**d**) *Left:* Flow cytometry analysis after Annexin V−FICT/PI dual staining was employed to evaluate the apoptosis of M2 macrophages treated with *h*rWISP-1 for 3 days. *Right*: Apoptotic cells were quantified as the sum of the percentages of early and late stages of apoptosis. (**e**) qRT-PCR analysis of relative mRNA levels of M1 (*Nos2*, *MhcII*, and *Il12p40*) and M2 (*Tgfβ1*, *Il10*, and *Il4*) markers in M2 macrophages treated with *h*rWISP-1 for 3 days. (**f**) ELISA of TNF-α, IL-1β, IL-4, and IL-13 in the culture supernatants of M2 macrophages treated with *h*rWISP-1 for 3 days. (**g**) Flow cytometry analysis of the population of $CD80^+$ and $CD206^+$ macrophages among M2 macrophages treated with *h*rWISP-1 for 2 or 3 days. Mean fluorescence intensity (MFI) values (*right*). (**c-g**) THP-1-derived M2 macrophages were transfected with control siRNA or siRNA targeting integrin αv, α5, β3, or β5 for 24 h prior to *h*rWISP-1 treatment (50 ng/ml). NS: not significant; **P* < 0.05, ***P* < 0.01, ****P* < 0.001, two-tailed Student's *t*-test. The data are from one experiment representative of three independent experiments with similar results (**a**, **b** ***upper***; **d** and **g** ***left***) or from three independent experiments (mean ± standard error: **b** ***lower***; **c, e**, **f**; **d** and **g** ***right***).

Supplementary Figure 9

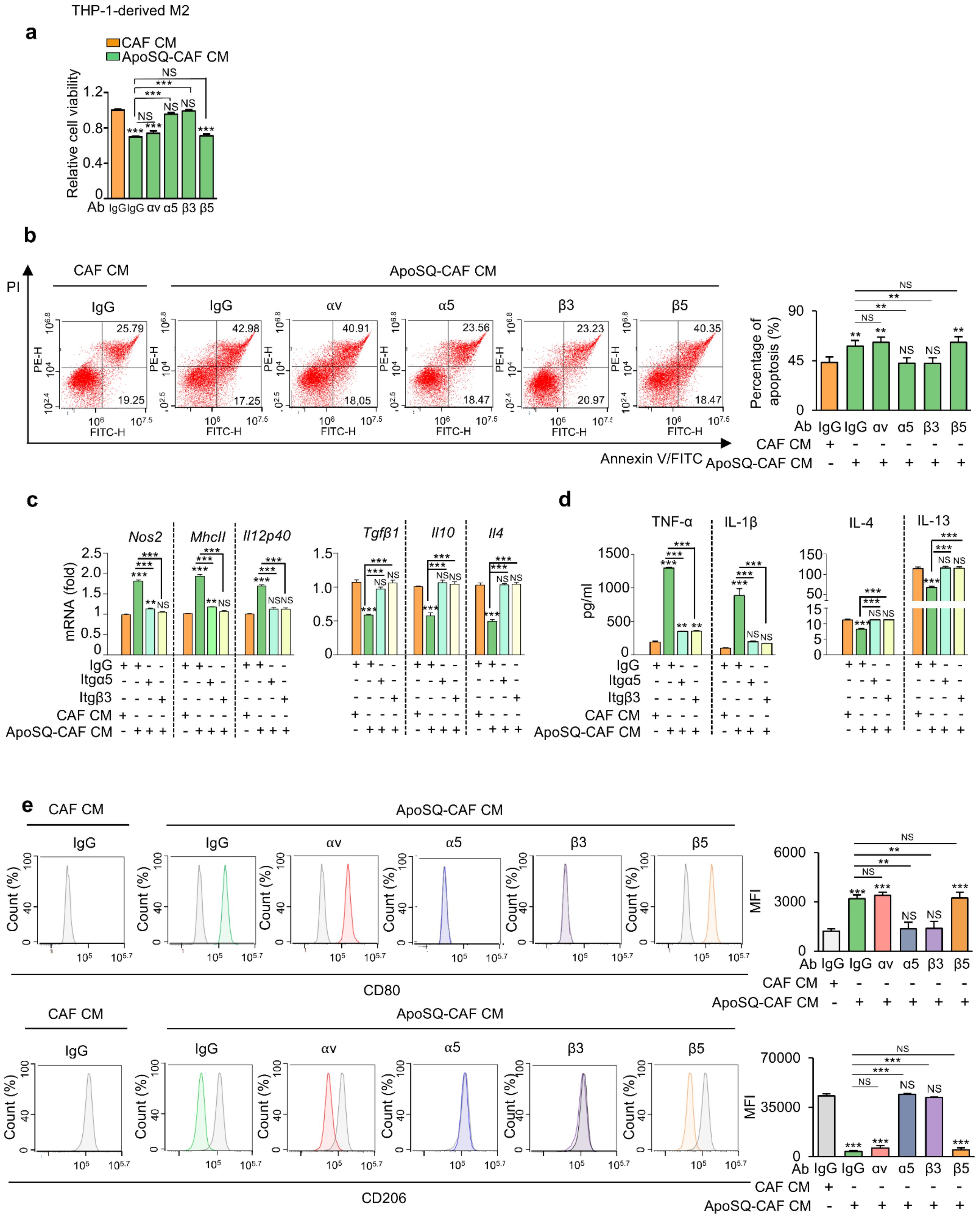

Supplementary Fig. S9. Neutralizing antibodies against integrin α5 and β3 abrogates the anti-survival, pro-apoptotic, and reprogramming effects of ApoSQ-CAF CM on M2 macrophages. (**a**) Cell viability assay of M2 macrophages (M2) treated with CM for 3 days. (**b**)

Left: Flow cytometry analysis after Annexin V−FICT/PI dual staining was employed to evaluate the apoptosis of M2 macrophages treated with CM for 3 days. *Right*: Apoptotic cells were quantified as the sum of the percentages of early and late stages of apoptosis. (**c**) qRT-PCR analysis of relative mRNA levels of M1 (*Nos2*, *MhcII*, and *Il12p40*) and M2 (*Tgfβ1*, *Il10*, and *Il4*) markers in M2 macrophages treated with CM for 3 days. (**d**) ELISA of TNF-α, IL-1β, IL-4, and IL-13 in the culture supernatants of M2 macrophages treated with CM for 3 days. (**e**) Flow cytometry analysis of the population of $CD80^+$ and $CD206^+$ macrophages among M2 macrophages treated with CM for 2 or 3 days. Mean fluorescence intensity (MFI) values (*right*). (**a-e**) THP-1-derived M2 macrophages were pretreated with an anti-integrin blocking antibody (3 μg/ml; anti-integrin αv, α5, β3, or β5) or corresponding IgG isotype control for 30 min prior to CM treatment. NS: not significant; $^{**}P < 0.01$, $^{***}P < 0.001$, two-tailed Student's *t*-test. The data are from one experiment representative of three independent experiments with similar results (**b** and **e** ***left***) or from three independent experiments (mean ± standard error: **a**, **c**, **d**; **b** and **e** ***right***).

Supplementary Figure 10

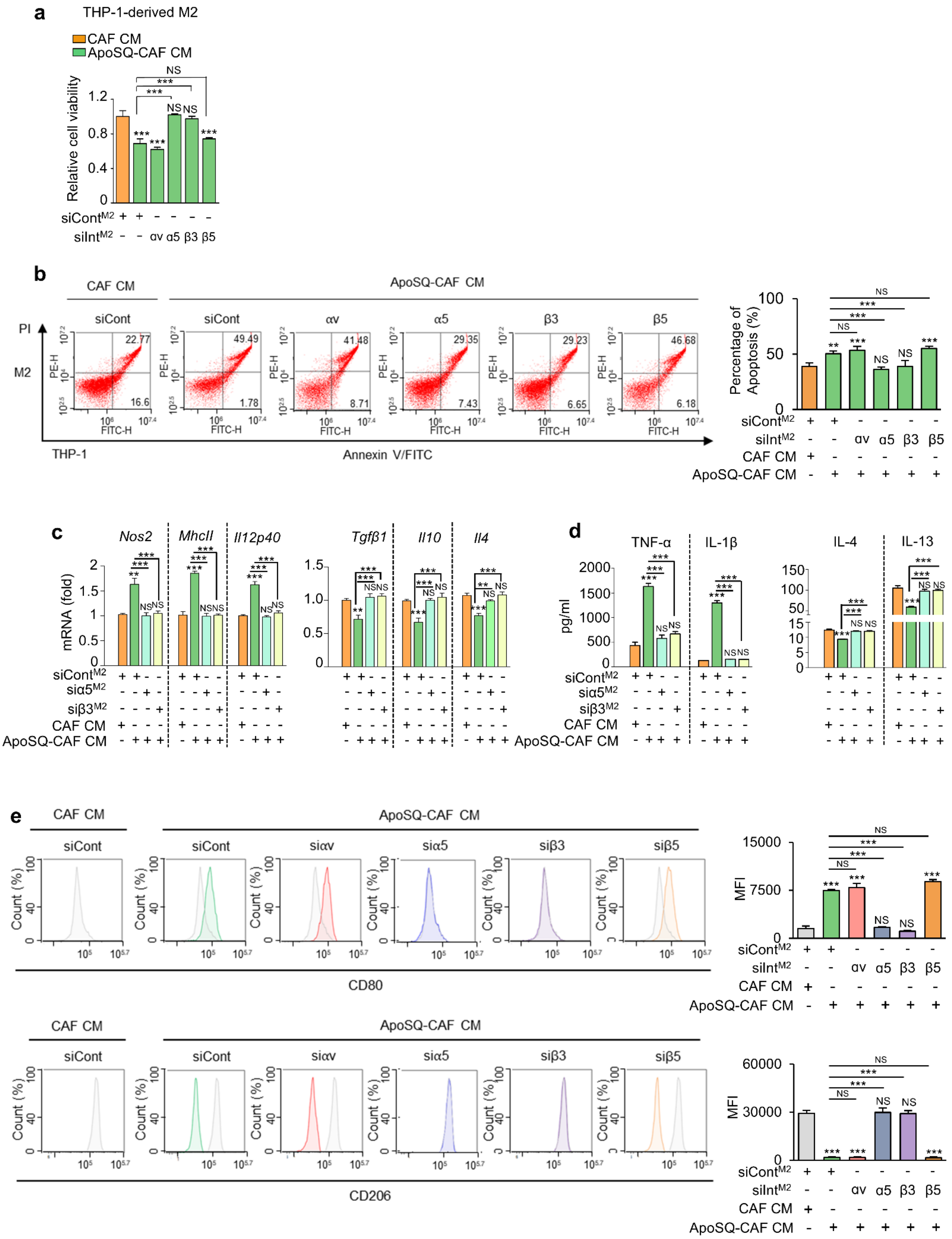

Supplementary Fig. S10. Knockdown of integrin α5 and β3 abrogates the anti-survival, pro-apoptotic, and reprogramming effects of ApoSQ-CAF CM on M2 macrophages. (**a**) Cell

viability assay of M2 macrophages (M2) treated with CM for 3 days. (**b**) *Left:* Flow cytometry analysis after Annexin V−FICT/PI dual staining was employed to evaluate the apoptosis of M2 macrophages) treated with CM for 3 days. *Right*: Apoptotic cells were quantified as the sum of the percentages of early and late stages of apoptosis. (**c**) qRT-PCR analysis of relative mRNA levels of M1 (*Nos2*, *MhcII*, and *Il12p40*) and M2 (*Tgfβ1*, *Il10*, and *Il4*) markers in M2 macrophages) treated with CM for 3 days. (**d**) ELISA of TNF-α, IL-1β, IL-4, and IL-13 in the culture supernatant of M2 macrophages treated with CM for 3 days. (**e**) Flow cytometry analysis of the population of $CD80^+$ and $CD206^+$ macrophages among M2 macrophages treated with CM for 2 or 3 days. Mean fluorescence intensity (MFI) values (*right*). (**a-e**) THP-1-derived M2 macrophages were transfected with control siRNA or siRNA targeting integrin αv, α5, β3, or β5 for 24 h prior to CM treatment. NS: not significant; **$P < 0.01$, ***$P < 0.001$, two-tailed Student's *t*-test. The data are from one experiment representative of three independent experiments with similar results (**b** and **e** ***left***) or from three independent experiments (mean ± standard error: **a**, **c**, **d**; **b** and **e** ***right***).

Supplementary Figure 11

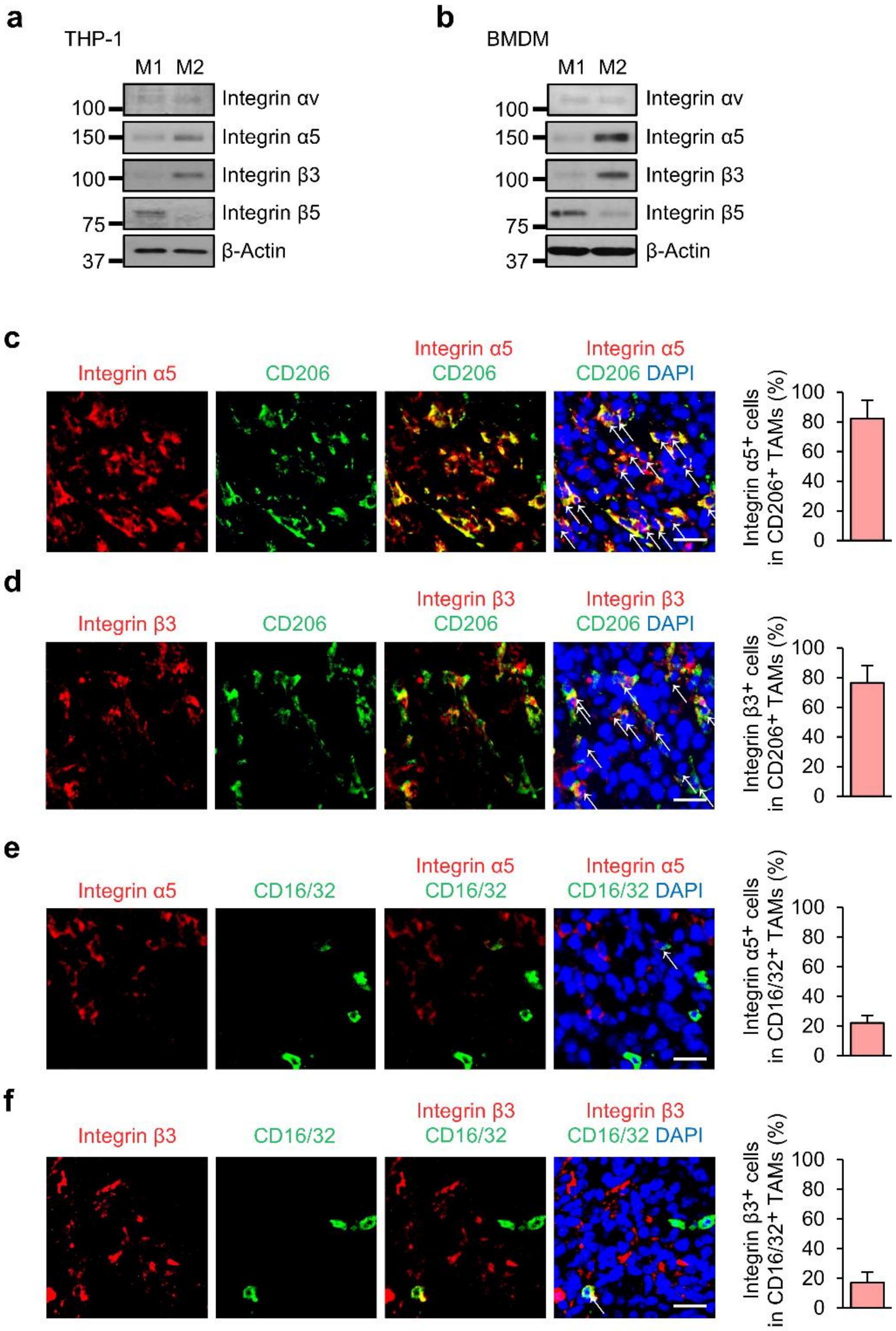

Supplementary Fig. S11. Integrin α5β3 is expressed on M2 macrophages and M2 TAMs in primary tumors. (**a, b**) Immunoblot analysis of Integrin αv, α5, β3, and β5 expression in M1 (M1) or M2 macrophages (M2) derived from THP-1 macrophages and mouse BMDMs. (**c**-**f**) Immunofluorescent staining of integrin α5 (red) and β3 (red), CD206 (green), and CD16/32 (green) in primary tumor sections. Right: Quantitation of integrin α5$^+$ or β3$^+$ cells in CD206$^+$ TAM (M2) and CD16/32$^+$ TAM (M1) in primary tumor. The imaging medium was VECTASHIELD fluorescent mounting medium containing DAPI. Original magnification: ×40. Scale bars = 100 μm. 344SQ cells

were subcutaneously implanted into syngeneic (129/Sν) mice (n=3 per group). Mice were necropsied 6 weeks after 344SQ cell injection. The data are from one experiment representative of three independent experiments with similar results (**a**, **b**; **c-f** ***left***) or from three independent experiments (mean ± standard error: **c-f** ***right***).

Supplementary Figure 12

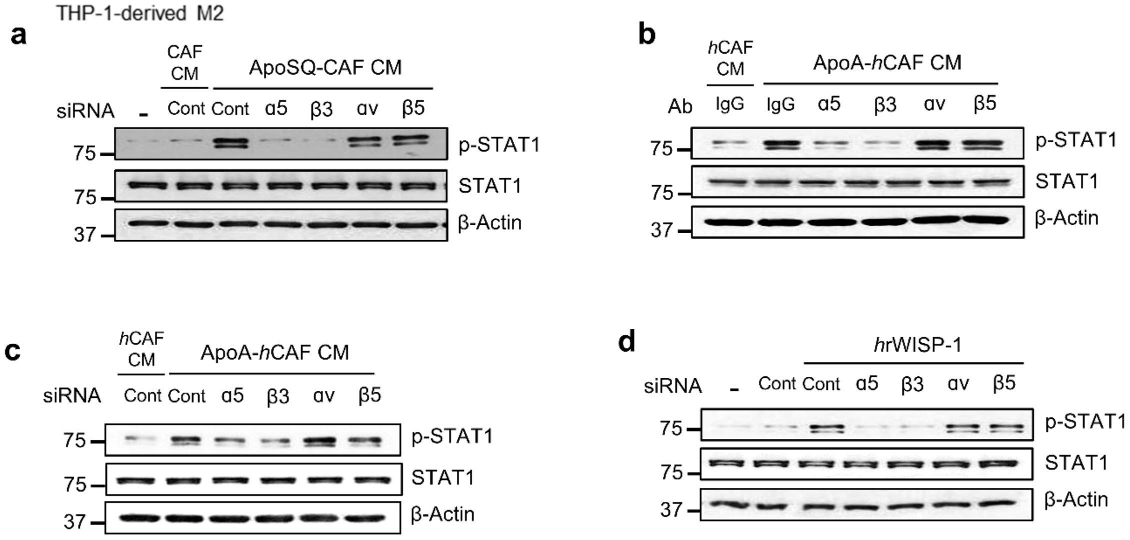

Supplementary Fig. S12. CM from CAFs exposed to apoptotic cancer cells and recombinant WISP-1 activates STAT1 through integrin α5β3. (**a-d**) Immunoblot analysis of phosphorylated STAT1 and total STAT1 in M2 macrophages treated with CM or *h*rWISP-1 for 30 min. (**a**, **c**, **d**) THP-1-derived M2 macrophages were transfected with control siRNA or siRNA targeting integrin αv, α5, β3, or β5 for 24 h prior to treatment with CM or *h*rWISP-1 (50 ng/ml) for 30 min. (**b**) THP-1-derived M2 macrophages were pretreated with an anti-integrin blocking antibody (3 μg/ml; anti-integrin αv, α5, β3, or β5) or corresponding IgG isotype control for 30 min prior to CM treatment. (**a-c**) CM was obtained from mouse CAFs (CAF) exposed to apoptotic 344SQ cells (ApoSQ) or human CAFs (*h*CAF) exposed to apoptotic A549 cells (ApoA). The data are from one experiment representative of three independent experiments with similar results.

Supplementary Figure 13

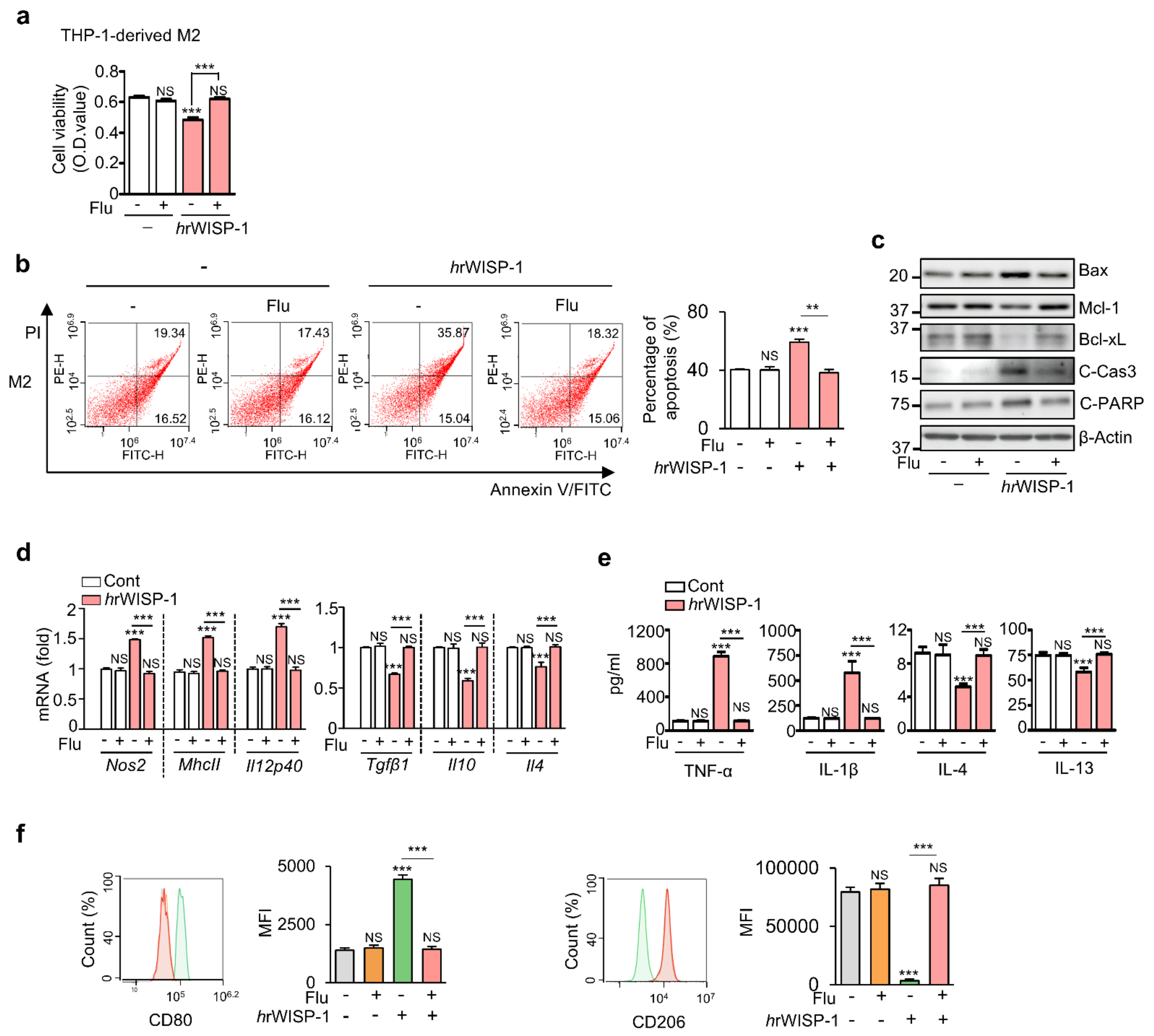

Supplementary Fig. S13. Pharmacological inhibition of STAT1 activation abrogates the anti-survival, pro-apoptotic, and reprogramming effects of WISP-1 on M2 macrophages. (**a**) Cell viability assay of M2 macrophages treated with *h*rWISP-1 (50 ng/ml) for 3 days. (**b**) *Left:* Flow cytometry analysis after Annexin V−FICT/PI dual staining was employed to evaluate the cell apoptosis of M2 macrophages treated with *h*rWISP-1 for 3 days. *Right*: Apoptotic cells were quantified as the sum of the percentages of early and late stages of apoptosis. (**c**) Immunoblot analysis of the indicated proteins in M2 macrophages treated with *h*rWISP-1 for 3 days. (**d**) qRT-PCR analysis of relative mRNA levels of M1 (*Nos2*, *MhcII*, and *Il12p40*) and M2 (*Tgfβ1*, *Il10*, and *Il4*) markers in THP-1-derived M2 macrophages treated with *h*rWISP-1 for 3 days. (e) ELISA of TNF-α, IL-1β, IL-4, and IL-13 in the culture supernatants of THP-1-derived M2 macrophages

treated with hrWISP-1 for 3 days. (**f**) Flow cytometry analysis of the population of $CD80^+$ and $CD206^+$ macrophages among M2 macrophages treated with *h*rWISP-1 for 2 or 3 days. Mean fluorescence intensity (MFI) values (*right*). (**a-f**) THP-1-derived M2 macrophages were pretreated with fludarabine (1 μM) l h prior to *h*rWISP-1 (50 ng/ml) treatment. NS: not significant; $^{**}P < 0.01$, $^{***}P < 0.001$, two-tailed Student's *t*-test. The data are from one experiment representative of three independent experiments with similar results (**b** and **f** ***left***; **c)** or from three independent experiments (mean ± standard error: **a**, **d**, **e**; **b** and **f** ***right***).

Supplementary Figure 14

a

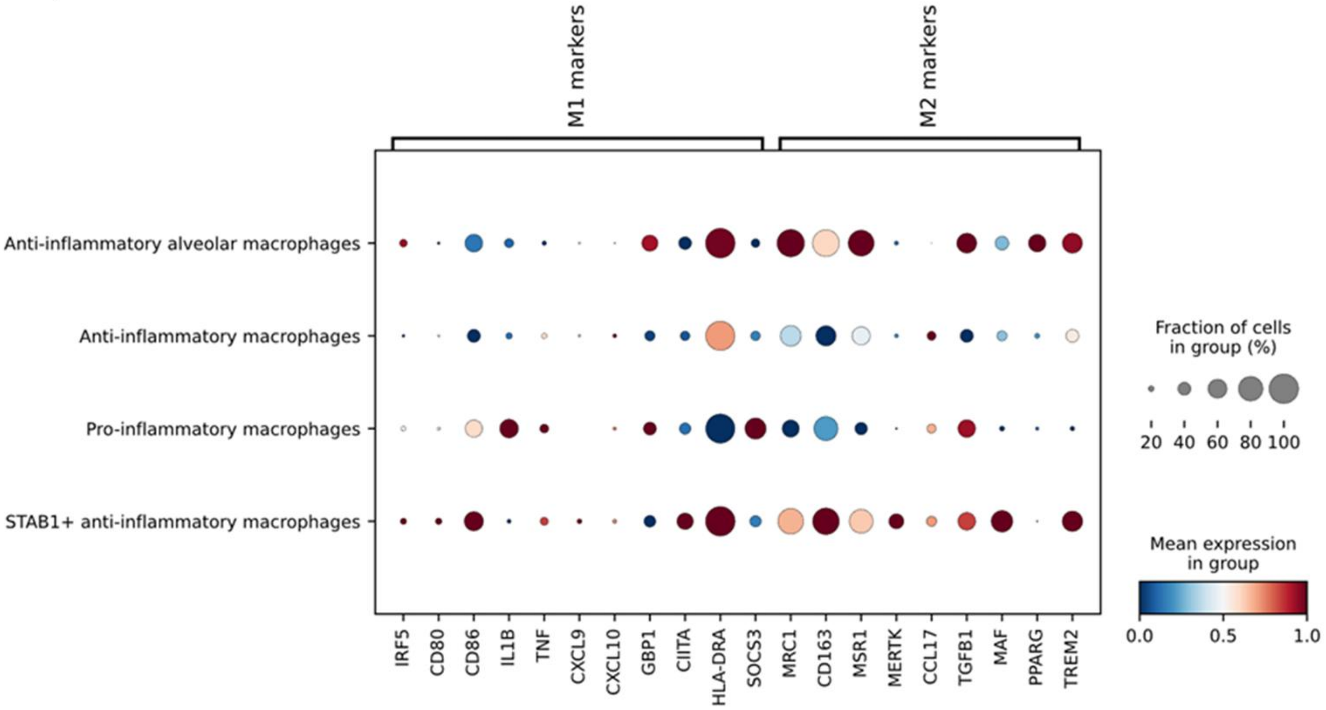

b

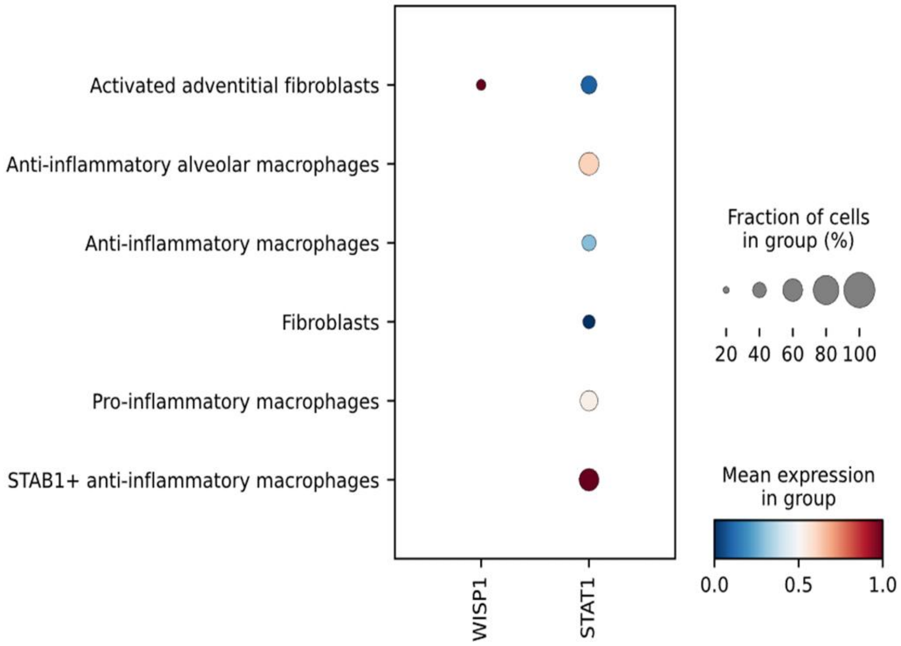

Supplementary Fig. S14. Expression of macrophage subtype markers and STAT1/CCN4 in non-small cell lung cancer (NSCLC) single-cell datasets.

Supplementary Figure 15

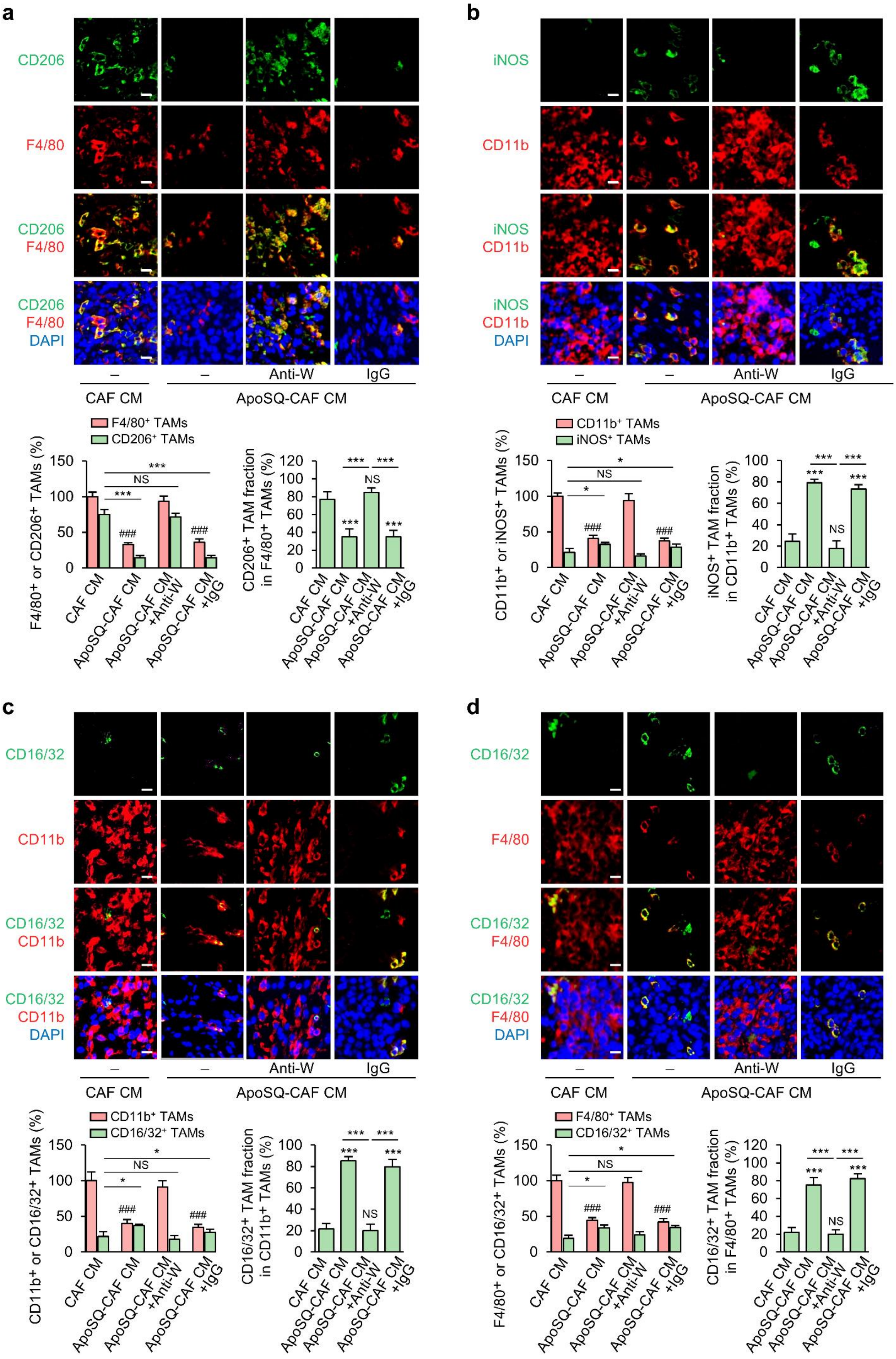

Supplementary Fig. S15. Administration of ApoSQ-CAF CM reduces M2 fraction and enhances M1 TAM fraction in primary tumors via WISP-1. The experimental design was described in Fig. 5a. (**a-d**) *Upper*: Immunofluorescent staining of primary tumor sections showing the M2 TAM Marker CD206 (green); M1 markers iNOS (green) and CD16/32 (green); and the pan-macrophage markers CD11b (red) and F4/80 (red). Original magnification: ×40. Scale Bar, 100 μm. (**a**) *Lower*: Quantitation of $CD206^+$ TAM (M2) density and the fraction of M2 TAMs. (**b-d**) *Lower*: Quantitation of $iNOS^+$ and $CD16/32^+$ TAM (M1) density and the fraction of M1 TAMs in primary tumors. The M1 and M2 TAM fraction was determined by the percentage of M1 and M2 TAMs within $CD11b^+$ or $F4/80^+$ TAMs. NS, not significant; $^*P < 0.05$, $^{***}P < 0.001$ compared to CAF CM or as indicated; $^{\#\#\#}P < 0.001$ compared to CAF CM, Analysis of variance with Tukey's post hoc test. The data are from one experiment representative of three independent experiments with similar results (**a-d *upper***). The data are represented as the means ± standard errors from three mice per group (**a-d *lower***).

Supplementary Figure 16

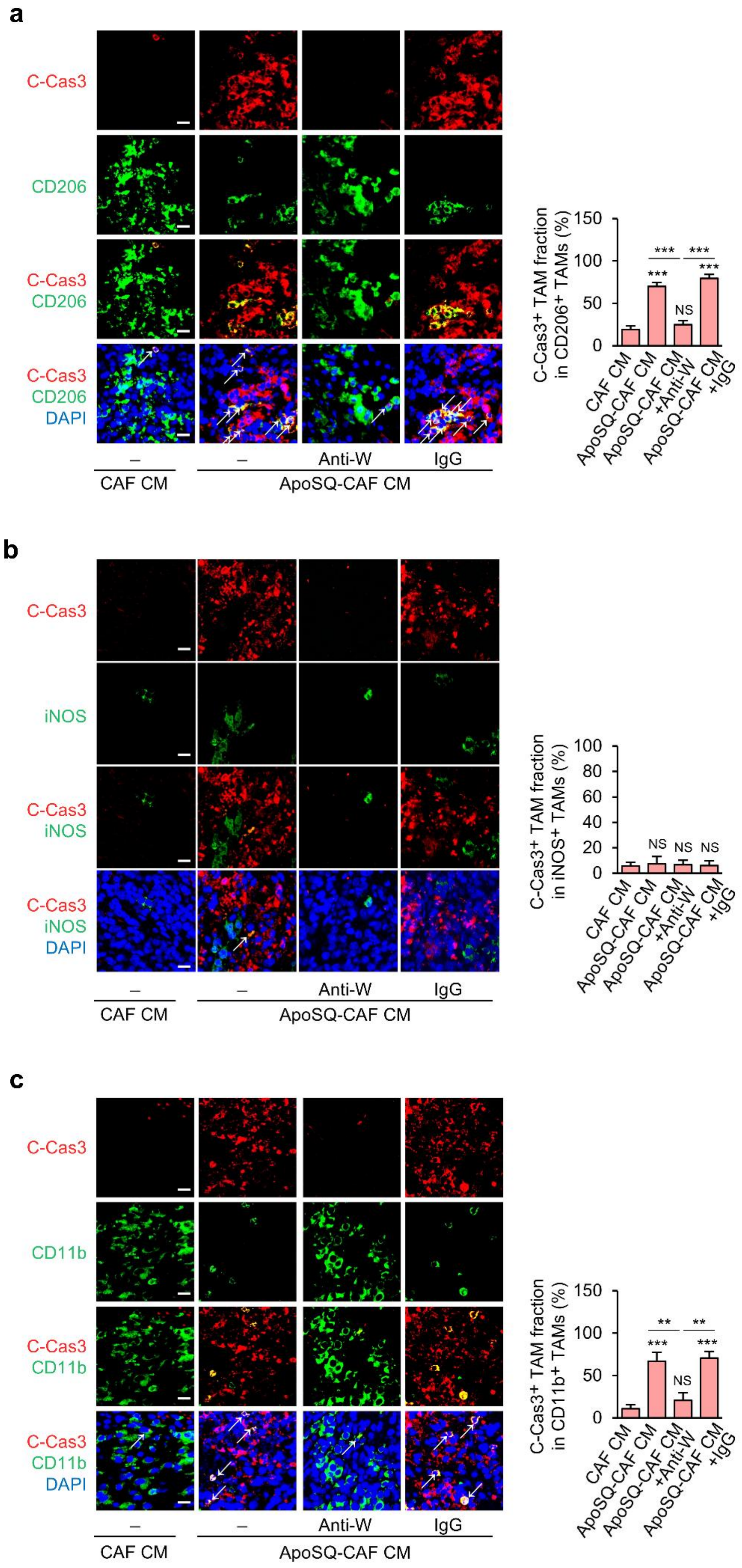

Supplementary Fig. S16. WISP-1 mediates the pro-apoptotic effect of ApoSQ-CAF CM in M2 TAMs *in vivo*. The experimental design was described in Fig. 5a. (**a-c**) Immunofluorescent staining of the apoptotic marker cleaved caspase-3 (red), the M2 TAM Marker CD206 (green), the M1 marker CD16/32 (green), the pan-macrophage marker CD11b (red), and DAPI (blue) in primary tumor sections. Original magnification: ×40. Scale Bar, 100 μm. Quantification of cleaved caspase-3^{+} cells among CD206^{+}, iNOS^{+}, or CD11b^{+} TAMs (*right*). NS, not significant; ***P* < 0.01, ****P* < 0.001, Analysis of variance with Tukey's post hoc test. The data are from one experiment representative of three independent experiments with similar results (**a-c *left***). The data are represented as the means ± standard errors from three mice per group (**a-c *right***).

Supplementary Figure 17

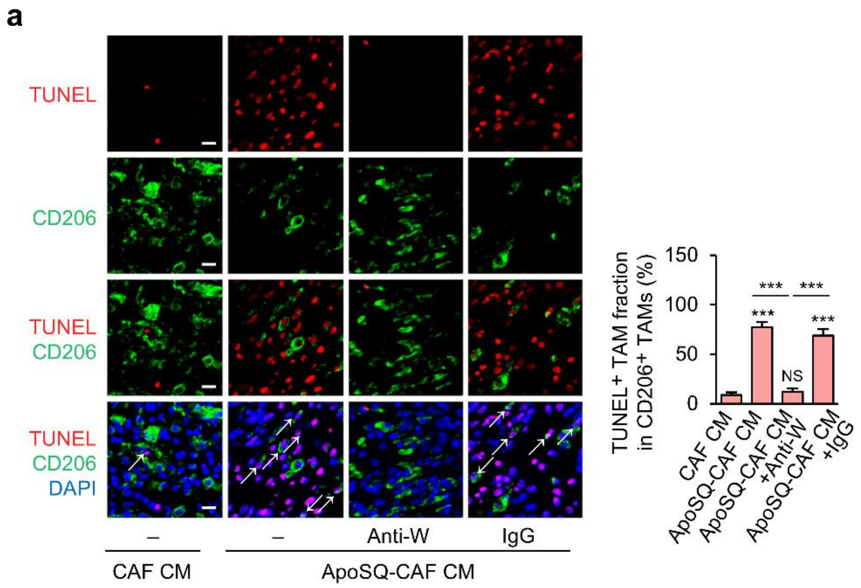

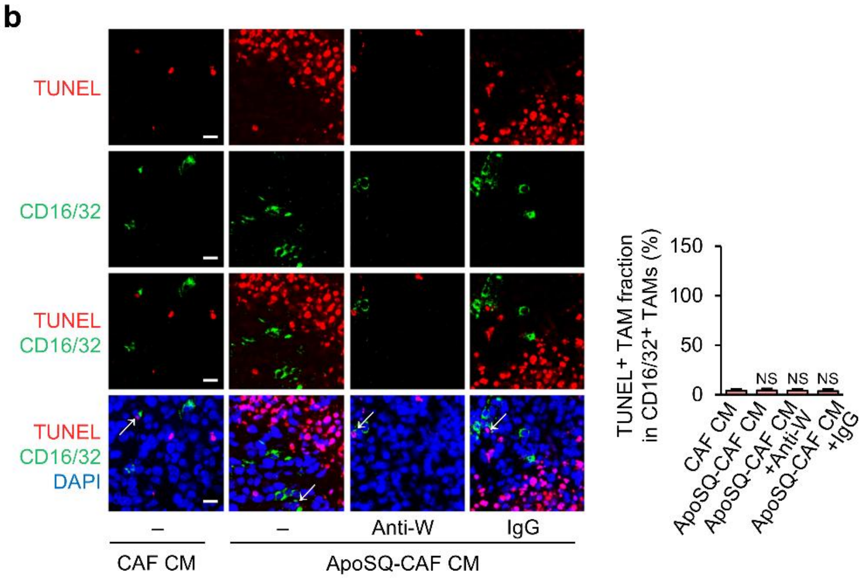

Supplementary Fig. S17. WISP-1 mediates increased TUNEL positive apoptosis in M2 TAMs by ApoSQ-CAF CM *in vivo*. The experimental design was described in Fig. 5a. (**a, b**) *Left:* Immunofluorescent staining of the TUNEL (red) the M2 TAM Marker CD206 (green), the M1 marker CD16/32^{+} (green), and DAPI (blue) in primary tumor sections. Original magnification: ×40. Scale Bar = 100 μm. *Right:* Quantification of TUNEL^{+} cells within CD206^{+} (**a**) or CD16/32^{+} TAMs (**b**). NS, not significant; ****P* < 0.01, Analysis of variance with Tukey's post hoc test. The data are from one experiment representative of three independent experiments with similar results (**a** and **b** ***left***). The data are represented as the means ± standard errors from three mice per group (**a** and **b** ***right***).

Supplementary Figure 18

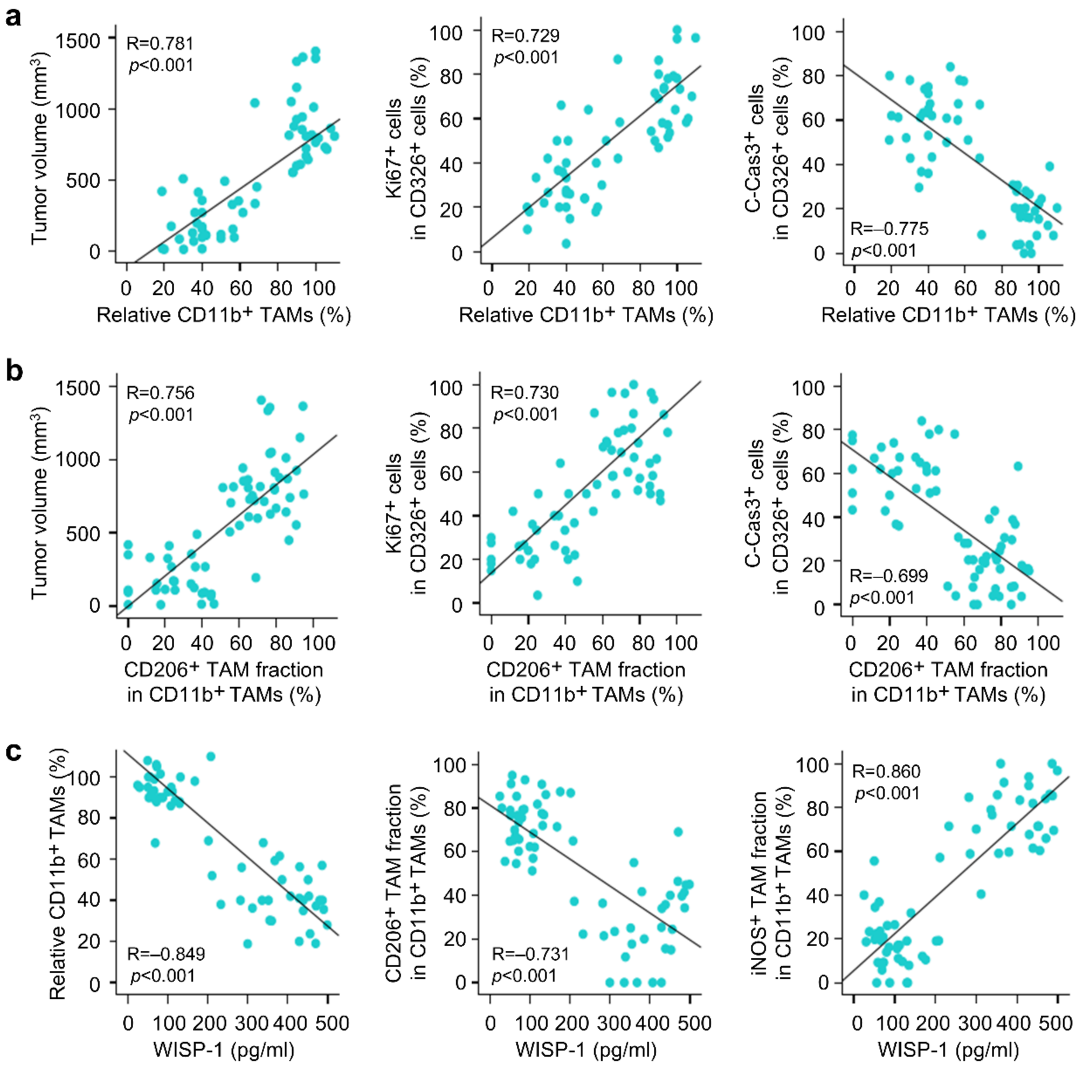

Supplementary Fig. S18. Pearson's correlation analyses of TAM subsets, tumor cell proliferation and apoptosis, and WISP-1 levels in CM. (**a**) Correlation between CD11b$^+$ TAM density in primary tumor tissue and tumor volume, Ki-67$^+$ or cleaved caspase3$^+$ cells among CD326$^+$ tumor cells. (**b**) Correlation between the proportion of CD206$^+$ M2 TAMs (CD206$^+$/CD11b$^+$) and tumor volume, Ki-67$^+$ or cleaved caspase-3$^+$ cells among CD326$^+$ tumor cells. (**c**) Correlations between WISP-1 levels in the CM evaluated by ELISA and CD11b$^+$ TAM density, the proportion of CD206$^+$ M2 TAMs, or iNOS$^+$ M1 TAMs (iNOS$^+$/ CD11b$^+$). p<0.001.

Supplementary Figure 19

a

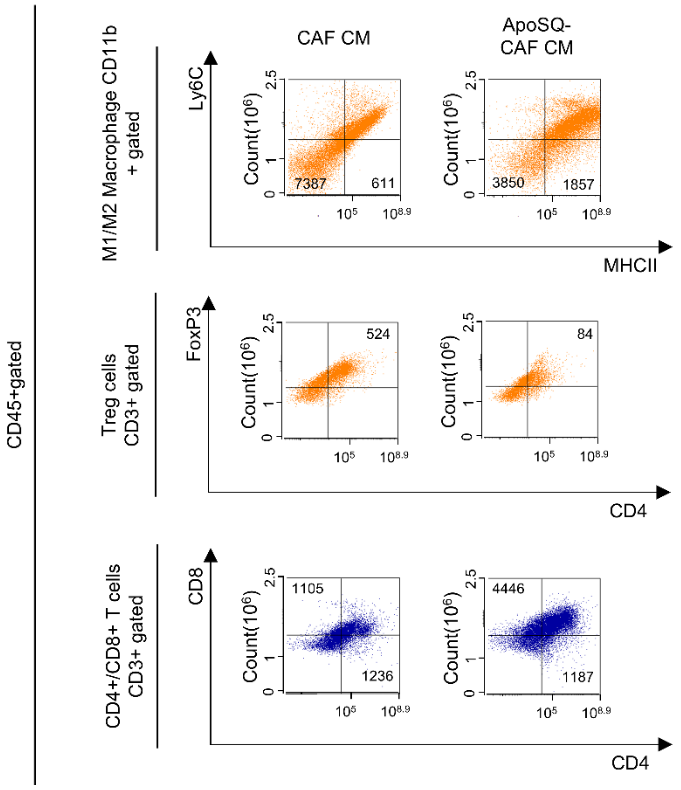

b

Immune population	Gating strategy
M2 TAMs	$CD45^+$ $CD11b^+$ $MHCII^-$ $Ly6C^-$
Treg cells	$CD45^+$ $CD3^+$ $FoxP3^+$ $CD4^+$
M1 TAMs	$CD45^+$ $CD11b^+$ $MHCII^+$ $Ly6C^-$
$CD8^+$ T cells	$CD45^+$ $CD3^+$ $CD8^+$ $CD4^-$
$CD4^+$ T cells	$CD45^+$ $CD3^+$ $CD4^+$ $CD8^-$

Supplementary Fig. S19. A representative histogram and gating strategy of immune cell analysis. (a) Representative histograms illustrating the gating strategy used to identify immune cell populations, including M1 and M2 macrophages, Tregs, $CD8^+$ T cells, and $CD4^+$ T cells, analyzed by flow cytometry. Histograms shown are representative of three independent experiments. Starting two days after subcutaneous injection of 344SQ cells into syngeneic (129/Sν) mice, intratumoral injections of CAF CM or ApoSQ-CAF CM were administered three times per week for six weeks (n = 6 mice per group). Mice were necropsied at the end of the treatment period. (b) Gating strategy for the immune cell population within primary tumors. Single-

cell suspensions were first gated on $CD45^+$ leukocytes, and specific immune cell subsets were subsequently identified based on marker expression.

Supplementary Figure 20

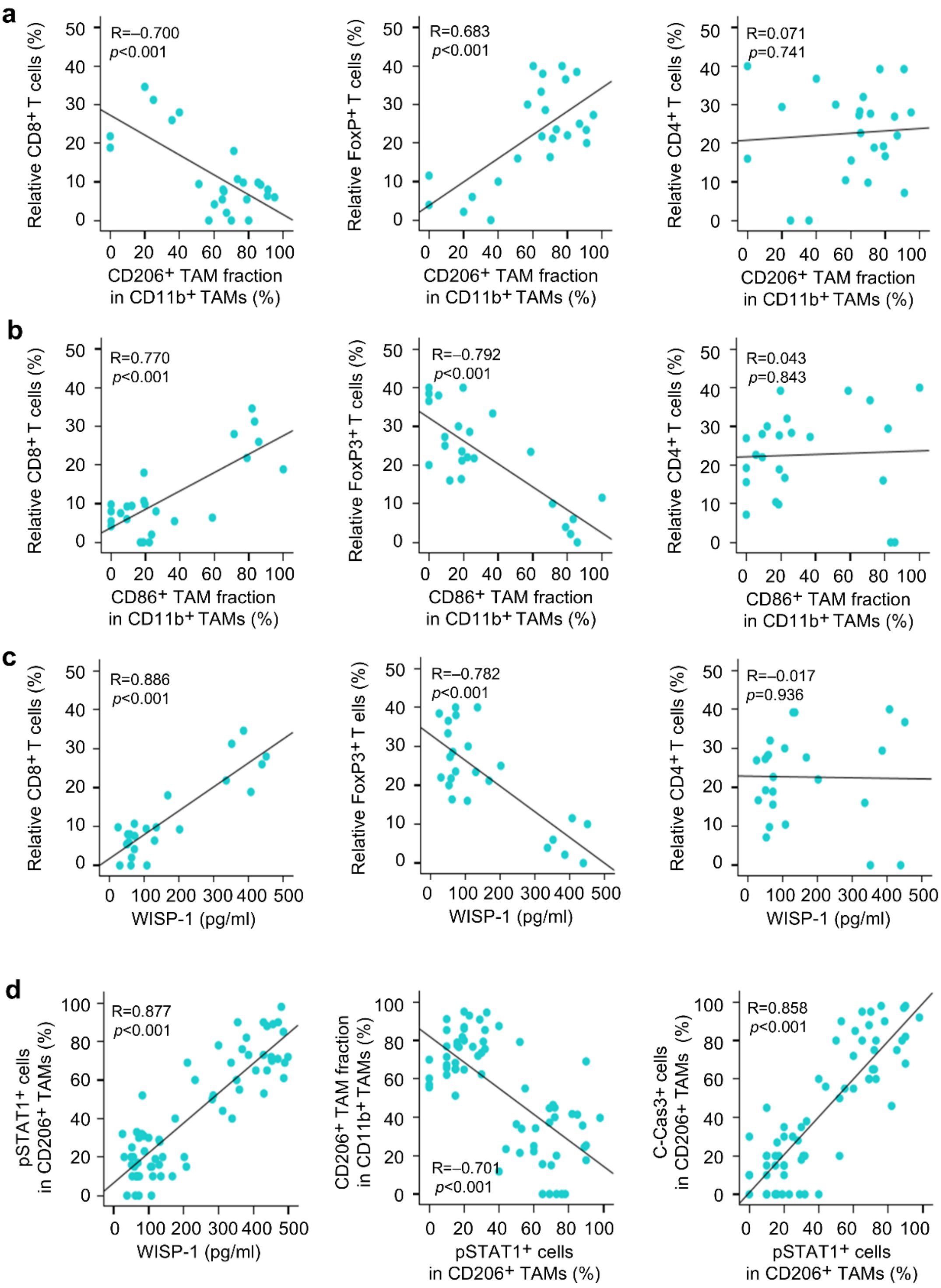

Supplementary Fig. S20. Pearson's correlation analyses of TAM subsets, T cell populations in primary tumor sections, and WISP-1 levels in CM. (**a**) Correlation between the proportion of $CD206^{+}$ M2 TAMs and the density of $CD8^{+}$, $FoxP3^{+}$, or $CD4^{+}$ T cells in primary tumor tissue. (**b**) Correlation between the proportion of $CD86^{+}$ M1 TAMs and the density of $CD8^{+}$, $FoxP3^{+}$, or $CD4^{+}$ T cells. (**c**) Correlation between WISP-1 levels in the CM (measured by ELISA) and the density of $CD8^{+}$, $FoxP3^{+}$, or $CD4^{+}$ T cells. (**d**) Correlations between phosphorylated $STAT1^{+}$ M2 TAMs

(phospho-STAT1^{+}/CD206^{+}) and WISP-1 the levels in the CM, the proportion of CD206^{+} M2 TAMs, or cleaved caspase-3^{+} cells among CD206^{+} M2 TAMs. $p<0.001$.

Supplementary Figure 21

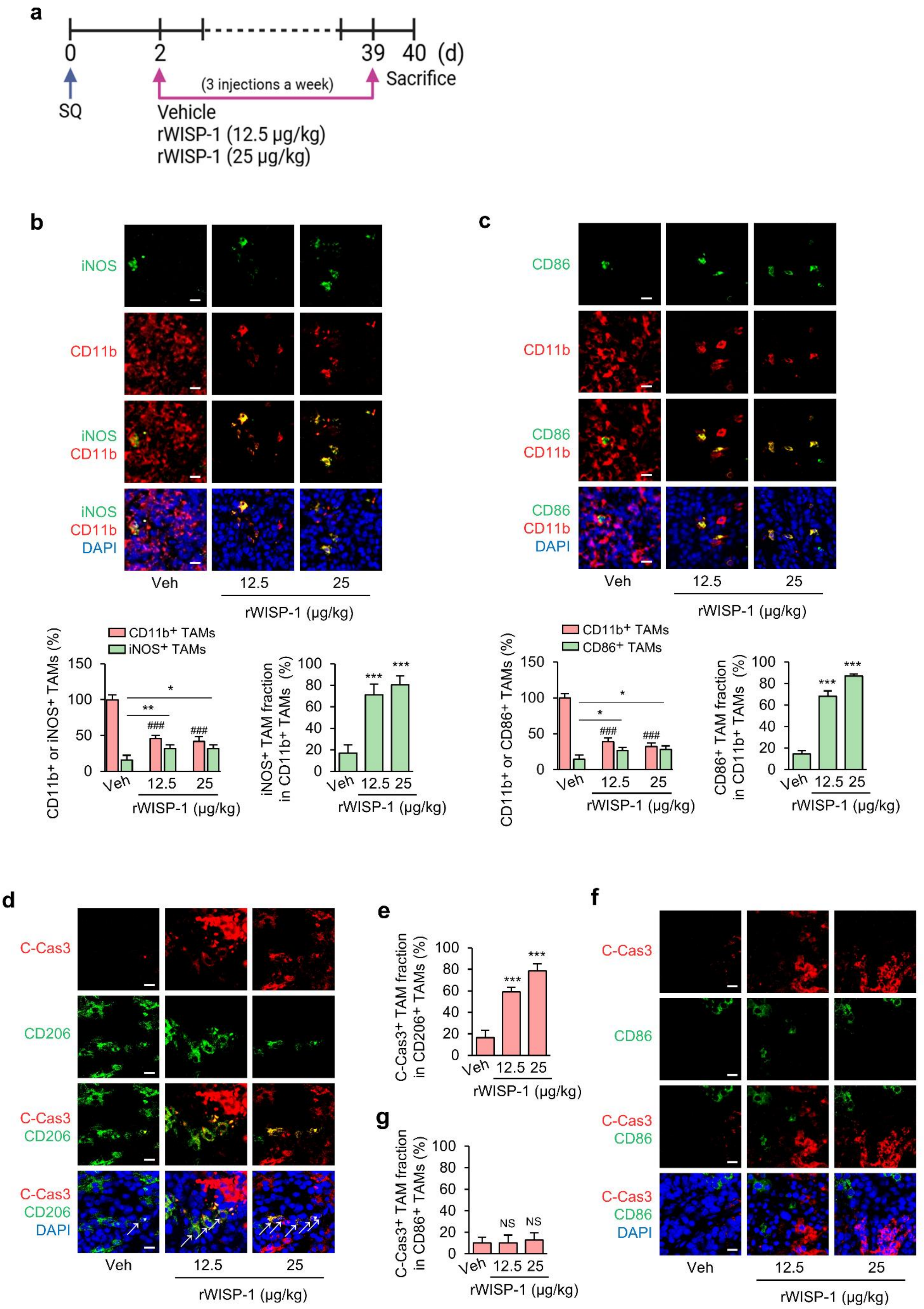

Supplementary Fig. S21. Administration of rWISP-1 reduces TAM density and M2 fraction, while promoting M2 apoptosis in primary tumors. (**a**) Schematic of experimental design and

treatment groups. Where indicated, rWISP-1 (12.5 and 25 μg/kg) was administered intratumorally three times a week for 6 weeks starting 2 days after subcutaneous injection of 344SQ cells into syngeneic (129/Sν) mice (n = 6 mice per group). Mice were necropsied 6 weeks later. (**b**, **c**) *Upper*: Immunofluorescent staining of M2 TAM Markers Arg1 (green) and CD206 (green) and the pan-macrophage marker CD11b (red) in primary tumor sections. Original magnification: ×40. Scale bars = 100 μm. *Lower*: Quantitation of $iNOS^+$ and $CD86^+$ TAM (M1) density (*left*) and the fraction of M1 TAMs (*right*) in primary tumor. The M1 TAM fraction was determined by the percentage of M1 TAMs within $CD11b^+$ TAMs. (**d**, **f**) Immunofluorescent staining of the apoptotic marker cleaved caspase-3 (red), the M2 TAM Marker CD206 (green), the M1 marker CD86 (green), and DAPI (blue) in primary tumor sections. Original magnification: ×40. Scale Bar, 100 μm. (**e**, **g**) Quantification of cleaved caspase-3^+ cells in $CD206^+$ or $CD86^+$ TAMs. NS, not significant; $^{*}P < 0.05$, $^{**}P < 0.01$, $^{***}P < 0.001$ compared to vehicle or as indicated; $^{\#\#\#}P < 0.001$ compared to vehicle, Analysis of variance with Tukey's post hoc test. The data are from one experiment representative of three independent experiments with similar results (**b** and **c** ***upper***; **d**, **f**). The data are represented as the means ± standard errors from three mice per group (**b** and **c** ***lower***; **e**, **g**).

Supplementary Figure 22

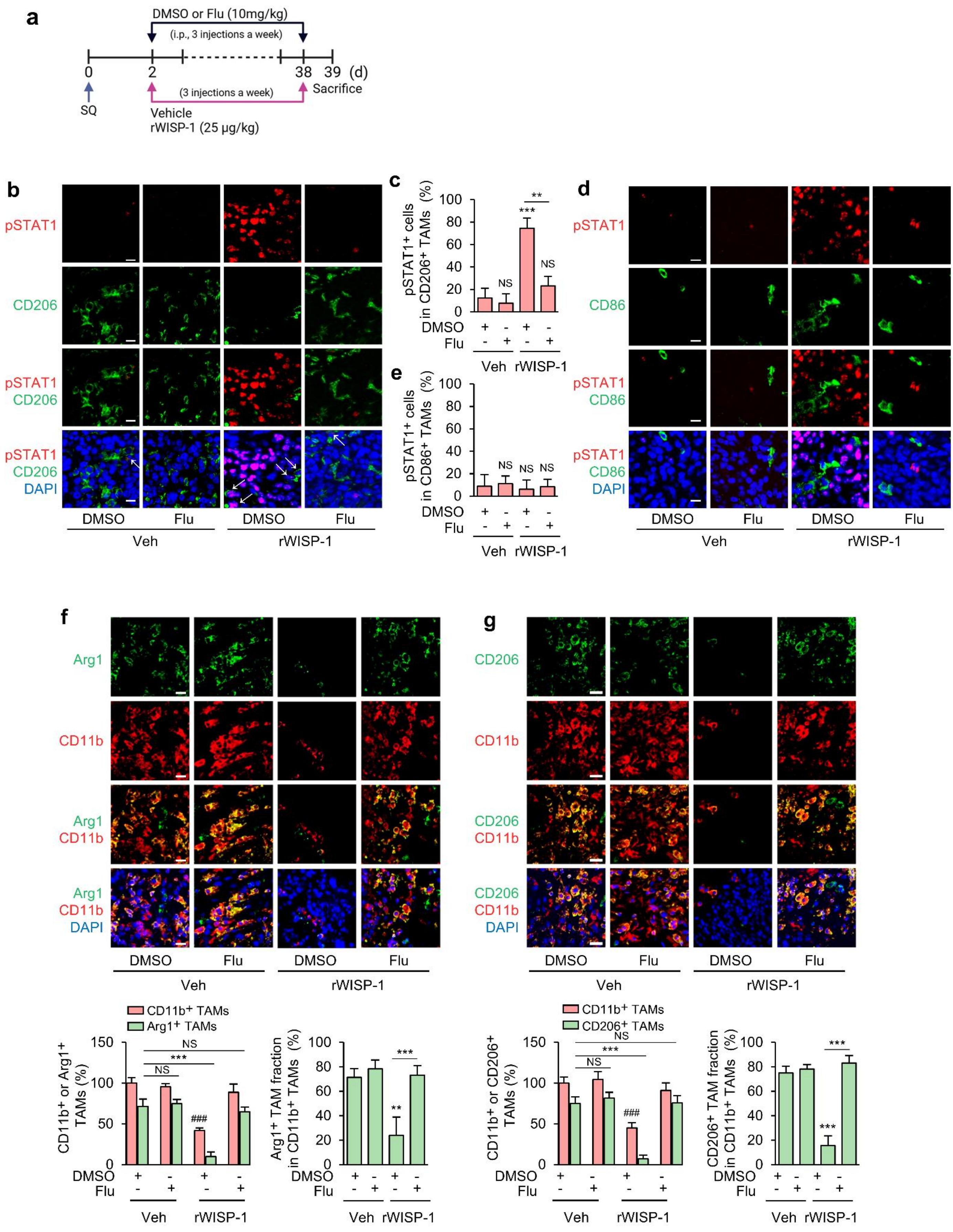

Supplementary Fig. S22. WISP-1-STAT1 signaling reduces TAM density and lowers the M2 fraction in primary tumors. (**a**) Schematic of experimental design and treatment groups. Where

indicated, fludarabine (10 mg/kg) or 5% DMSO was administrated intraperitoneally in conjunction with the intratumoral injection of rWISP-1 (25 μg/kg). rWISP-1 was intratumorally injected three times per week for 6 weeks, starting 2 days after the subcutaneous implantation of 344SQ cells into syngeneic (129/Sv) mice (n = 5 mice per group). Mice were necropsied at the end of the 6-week treatment period. (**b**, **d**) Representative confocal images of primary tumor sections stained with an anti-phosphorylated STAT1 (red), anti-CD206 antibody (green), anti-CD86 antibody (green), and DAPI (blue). Original magnification: ×40. Scale bars = 100 μm. (**c**, **e**) Quantification of phosphorylated $STAT1^{+}$ cells among $CD206^{+}$ cells or $CD86^{+}$ TAMs. (**f**, **g**) *Upper*: Immunofluorescent staining of primary tumor sections showing M2 TAM Markers Arg1 (green) and CD206 (green), along with the pan-macrophage marker CD11b (red). Original magnification: ×40. Scale bars = 100 μm. *Lower*: Quantitation of $Arg1^{+}$ and $CD206^{+}$ TAM (M2) density (*left*) and the fraction of M2 TAMs (*right*) in primary tumors. The fraction of M2 TAMs were determined by the percentage of M2 TAMs within $CD11b^{+}$ TAMs. NS, not significant; $^{**}P < 0.01$, $^{***}P < 0.001$ compared to DMSO or as indicated; $^{\#\#\#}P < 0.001$ compared to DMSO, Analysis of variance with Tukey's post hoc test. The data are from one experiment representative of three independent experiments with similar results (**b**, **d**; **f** and **g** ***upper***). The data are represented as the means ± standard errors from three mice per group (**c**, **e**; **f** and **g** ***lower***).

Supplementary Figure 23

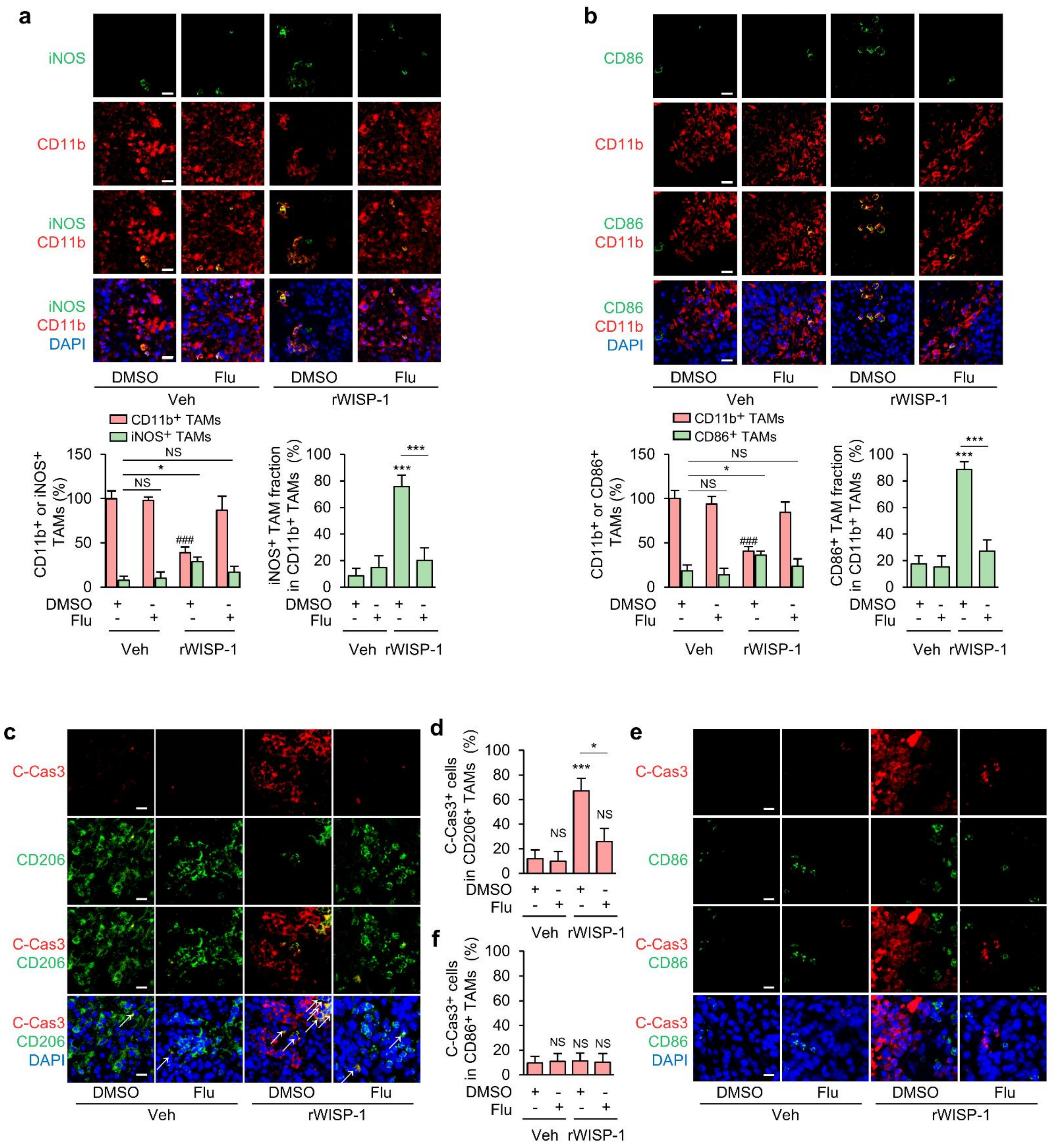

Supplementary Fig. S23. WISP-1-STAT1 signaling increases M1 TAM fraction and induces apoptosis of M2 TAMs in primary tumors. Where indicated, fludarabine (10 mg/kg) or 5% DMSO was administrated intraperitoneally in conjunction with intratumoral injection of rWISP-1 (25 μg/kg). rWISP-1 was injected three times per week for 6 weeks, starting 2 days after the subcutaneous implantation of 344SQ cells into syngeneic (129/Sv) mice (n = 5 mice per group). Mice were necropsied at the end of the 6-week treatment period. (**a**, **b**) *Upper*: Immunofluorescent

staining of primary tumor sections showing M1 TAM Markers iNOS (green) and CD86 (green), along with the pan-macrophage marker CD11b (red). Original magnification: ×40. Scale bars = 100 μm. *Lower*: Quantitation of $iNOS^+$ and $CD86^+$ TAM (M1) density (*left*) and the fraction of M1 TAMs (*right*) in primary tumors. The M1 TAM fraction was determined by the percentage of M1 TAMs within $CD11b^+$ TAMs. (**c**, **e**) Immunofluorescent staining of the apoptotic marker cleaved caspase-3 (red), the M2 TAM Marker CD206 (green), the M1 marker CD86 (green), and DAPI (blue) in primary tumor sections. Original magnification: ×40. Scale Bar, 100 μm. (**d**, **f**) Quantification of cleaved caspase-3^+ cells among $CD206^+$ or $CD86^+$ TAMs (*right*). NS: not significant; $^*P < 0.05$, $^{***}P < 0.001$, compared to DMSO or as indicated; $^{\#\#\#}P < 0.001$ compared to DMSO, Analysis of variance with Tukey's post hoc test. The data are from one experiment representative of three independent experiments with similar results (**a** and **b** ***upper***; **c**, **e**). The data are represented as the means ± standard errors from three mice per group (**a** and **b** ***lower***; **d**, **f**).

Supplementary Figure 24

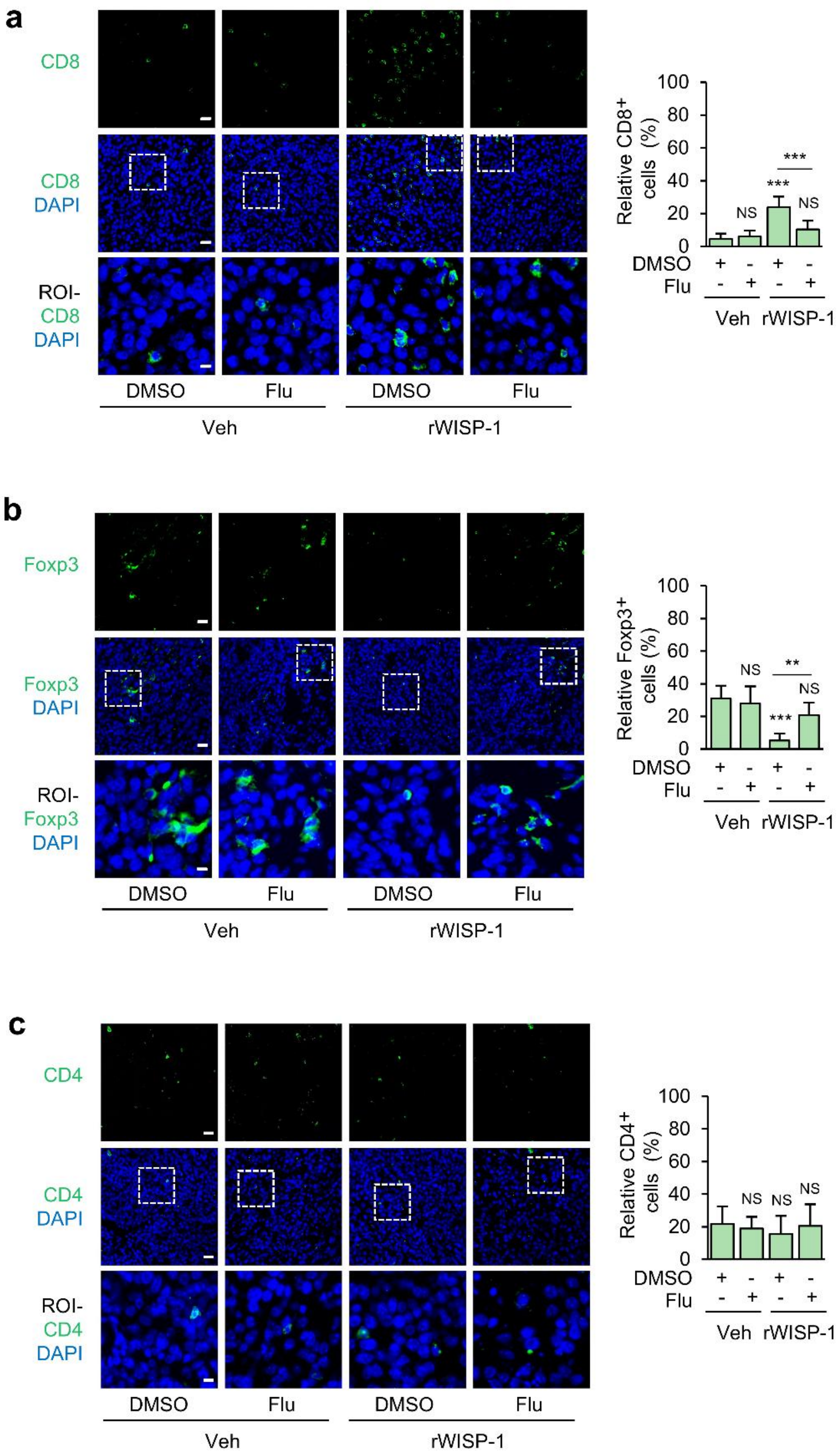

Figure 24. WISP-1-STAT1 signaling rebalances T cell populations in primary tumors. Where indicated, fludarabine (10 mg/kg) or 5% DMSO was administrated intraperitoneally in conjunction with the intratumoral injection of rWISP-1 (25 μg/kg). rWISP-1 was intratumorally injected three times per week for 6 weeks, starting 2 days after the subcutaneous implantation of 344SQ cells into syngeneic (129/Sv) mice (n = 5 mice per group). Mice were necropsied at the end of the 6-week treatment period. (**a-c**) *Left*: Immunofluorescent staining of CD8 (green), FoxP3 (green),

CD4 (green), and DAPI (blue) in primary tumor sections. Original magnification: ×40. Scale Bar, 100 μm. *Right*: Quantification of $CD8^+$, $Foxp3^+$, and $CD4^+$ T cell densities. NS, not significant; $^{**}P <$ 0.01, $^{***}P < 0.001$ compared to DMSO or as indicated, Analysis of variance with Tukey's post hoc test. The data are from one experiment representative of three independent experiments with similar results (**a-c *left***). The data are represented as the means ± standard errors from three mice per group (**a-c *right***).

Table S1. List of antibodies used for this study

Antigen	Vendor	Cat.No	Source	Species cross-reactivity	Application	Dilution
Arg1	Novus	NB100-59740	G	H, M, R	IHC	1:100
	Cell signaling	93668	Rb	H, M, R	IB	1:1000
Bax	Cell signaling	2772	Rb	H, M, R	IB	1:1000
Bcl-xL	Cell signaling	2764	Rb	H, M, R	IB	1:1000
Bcl-2	Cell signaling	3498	Rb	H, M	IB	1:1000
C-Cas3	Cell Signaling	9661	Rb	H, M, R, Mk	IB	1:1000
					IHC	1:100
CD11b	NB110-89474	Novus	Rb	H, M, R, B, C, Pm, RM	IHC	1:100
	MA1-80091	Invitrogen	R	H,M,R	IHC	1:200
CD16/32	Abcam	ab223200	Rb	M	IHC	1:200
	NBP1-27946	Novus	R	M	IHC	1:100
CD163	Abcam	ab182422	Rb	H, M, R	IB	1:1000
CD206	Abcam	ab64693	Rb	H, M, R	IHC, ICC	1:200
	Invitrogen	MA5-16871	R	M	IHC	1:200
	Cell signaling	24595	Rb	H, M, R	IB	1:1000
CD326	BD	552370	R	M	IHC	1:100
CD86	GeneTex	GTX-34569	M	H, M, R	ICC	1: 200
C-PARP	Cell signaling	5625	Rb	H, M	IB	1:1000
F4/80	Cell signaling	70076	Rb	M	IHC	1:100
	Abcam	ab16911	R	H, M	IHC	1:100
IL12P40	abcam	ab106270	Rb	H, M, R	IB	1:1000
iNOS	Invitrogen	PA1-036	Rb	H, M, R	IHC	1:200
	Invitrogen	PA1-036	Rb	H, M, R	IB	1:1000
Integrin αv	Cell signaling	60869	Rb	H, M, R	IB	1:1000
	Invitrogen	14-0512-85	Rb	H, M	Neutralizing	5µg/ml
Integrin α5	Abcam	ab150361	Rb	H, M, R	IHC	1:200
	Cell signaling	98204	Rb	H, M, R	IB	1:1000
	Santa cruz	sc-376199	M	H, M, R	IP	2µg/ml
	Invitrogen	PA5-79529	Rb	H, M	Neutralizing	5µg/ml
Integrin β3	Invitrogen	11-0611-82	Hm	M, R	IHC	1:100
	Cell signaling	13166	Rb	H, M, R	IB	1:1000
	Santa cruz	sc-365679	M	H, M, R	IP	2µg/ml
	Invitrogen	MA1-35264	M	H	Neutralizing	5µg/ml
Integrin β5	Cell signaling	3629	Rb	H, M, R	IB	1:1000
	Invitrogen	14-0497-82	M	H, M	Neutralizing	5µg/ml
Mcl-1	Cell signaling	5453	Rb	H, M	IB	1:1000
MHCII	Invitrogen	PA5-116876	Rb	H, M, R	IB	1:1000
p-STAT-1	Cell signaling	7649	Rb	H, M, R	IB	1:1000
	Santa cruz	sc-7988	G	H, M	ICC	1:200
					IHC	1:200
p21	Santa cruz	sc-6246	M	H, M, R	IB	1:1000
					ICC	1:200
p53	Cell signaling	2524	M	H, M, R, Hm, Mk	IB	1:1000
STAT-1	Cell signaling	9172	Rb	H, M, R	IB	1:1000
WISP-1	Abcam	ab260036	Rb	M, R	IB, IP	1:1000, 2µg/ml
β-actin	Santa cruz	sc-69879	M	Broad species	IB	1:1000
Goat IgG (Alexa 488)	Thermo fisher scientific	A11055	D	Not applicable	ICC	1:1000
IgG	R&D Systems	MAB005	R	H, M	Neutralizing	5µg/ml
Mouse IgG (Alexa 568)	Thermo fisher scientific	A11061	Rb	Not applicable	ICC	1:1000
Mouse IgG (HRP)	GeneTex	GTX213111	G	Not applicable	IB	1:5000
Rabbit IgG (Alexa 488)	Thermo fisher scientific	A11008	G	Not applicable	ICC	1:1000
Rabbit IgG (HRP)	GeneTex	GTX213110	G	Not applicable	IB	1:5000

Abbreviation: H-Human, M-Mouse, R-Rat, Rb-Rabbit, G-Goat, B-Bovine, C- C. Elegans, Pm-Primate, RM-Rhesus Macaque, Mk-Monkey, D-Donkey, Hm-Hamster IB-Immunoblotting, IHC-Immunoprecipitation,IP-Immunoprecipitation, IF-Immunofluorescence

Table S2. Sequences of qRT-PCR primer.

Target gene	Forward (5'→ 3')	Reverse (5'→ 3')
Human *Il4*	CCGTAACAGACATCTTTGCTGCC	GAGTGTCCTTCTCATGGTGGCT
Mouse *Il4*	ATCATCGGCATTTTGAACGAGGTC	ACCTTGGAAGCCCTACAGACGA
Mouse *Il4*	CGAGCTCACTCTCTGTGGTG	TGAACGAGGTCACAGGAGAA
Human *Tgfβ1*	TACCTGAACCCGTGTTGCTCTC	GTTGCTGAGGTATCGCCAGGAA
Mouse *Tgfβ1*	TGGAGCAACATGTGGAACTC	TGCCGTACAACTCCAGTGAC
Human *Il10*	TCTCCGAGATGCCTTCAGCAGA	TCAGACAAGGCTTGGCAACCCA
Mouse *Il10*	CGGGAAGACAATAACTGCACCC	CGGTTAGCAGTATGTTGTCCAGC
Mouse *Il10*	GCTCTTACTGACTGGCATGAG	CGCAGCTCTAGGAGCATGTG
Human *Il12p40*	GACATTCTGCGTTCAGGTCCAG	CATTTTTGCGGCAGATGACCGTG
Mouse *Il12p40*	TTGAACTGGCGTTGGAAGCACG	CCACCTGTGAGTTCTTCAAAGGC
Human *Nos2*	GCTCTACACCTCCAATGTGACC	CTGCCGAGATTTGAGCCTCATG
Mouse *Nos2*	GAGACAGGGAAGTCTGAAGCAC	CCAGCAGTAGTTGCTCCTCTTC
Mouse *Nos2*	GACATTACGACCCCTCCCAC	GCACATGCAAGGAAGGGAAC
Human *MhcII*	GAGCAAGATGCTGAGTGGAGTC	CTGTTGGCTGAAGTCCAGAGTG
Mouse *MhcII*	GTGTGCAGACACAACTACGAGG	CTGTCACTGAGCAGACCAGAGT
Mouse *Arg1*	GTGGGGAAAGCCAATGAAG	GCTTCCAACTGCCAGACTGT
Mouse *Cd206*	CTAACTGGGGTGCTGACGAG	GGCAGTTGAGGAGGTTCAGT
Mouse *Cd163*	GGCTAGACGAAGTCATCTGCAC	CTTCGTTGGTCAGCCTCAGAGA
Mouse *Tnfα*	CCCCAAAGGGATGAGAAGTT	CACTTGGTGGTTTGCTACGA
Mouse *Cd80*	CCTCAAGTTTCCATGTCCAAGGC	GAGGAGAGTTGTAACGGCAAGG
Mouse *Ifng*	CAGCAACAGCAAGGCGAAAAAGG	TTTCCGCTTCCTGAGGCTGGAT
Hprt	CAGACTGAAGAGCTACTGTAATG	CCAGTGTCAATTATATCTTCAAC

Table S3. List of siRNA

Target gene	Sense	Antisense
Human *Stat1*	5’-CUGGAUAUAUCAAGACUGA-3’	5’-UCAGUCUUGAUAUAUCCAG-3’
Mouse *Stat1*	5’-CACAGUUUUAUCCUGAG-3’	5’-UCUCAGGAUAAAACUGUG-3’
Mouse Wisp-1	5′-GGAAUCCUAACGAUAUCUU-3’	5′- AAGAUAUCGUUAGGAUUCC-3′
Human *integrin αv*	5′-CCGAAACAAUGAAGCCUUA-3’	5′-UAAGGCUUCAUUGUUUCGG-3’
Human *integrin α5*	5'-CUGUUGAAGGUACAUCGUU-3'	5'-AACGAUGUACCUUCAACAG-3'
Human *integrin β3*	5′-UUACUGCCGUGACGAGAUU-3’	5′-AAUCUCGUCACGGCAGUAA-3’
Human *integrin β5*	5'-CAGCUAAGAACCUGGAACU-3'	5'-AGUUCCAGGUUCUUAGCUG-3'

Table S4. List of antibodies employed for flow cytometry

Antigen	Vendor	Cat.No	Source	Clone	Dilution
CD16	BD Bioscience	335035	Mouse	NKP15(IVD)	1:500
CD206	eBioscience	12-2061-82	Mouse	MR6F3	1:100
CD163	eBioscience	12-1631-82	Mouse	TNKUPJ	1:100
MHCII	eBioscience	25-5321-82	Mouse	M5/114.15.2	1:100
CD45	BD Bioscience	553087	Rat	RA3-6B2(RUO)	1:250
CD11b	BD Bioscience	557397	Rat	M1/70(RUO)	1:250
Ly6C	BD Bioscience	552093	Rat	RB6-8C5(RUO)	1:250
FoxP3	BD Bioscience	563902	Rat	R16-715(RUO)	1:250
CD4	BD Bioscience	552775	Rat	RM4-5(RUO)	1:250
CD3	BD Bioscience	555275	Rat	17A2(RUO)	1:250
CD8	BD Bioscience	561092	Rat	53-6.7(RUO)	1:250

Table S5. Macrophage and fibroblast populations in the single-cell transcriptomic dataset

Cell types (authors annotation)	Number of cells
Anti-inflammatory macrophages	44,399
Pro-inflammatory macrophages	14,140
$STAB1^{+}$ anti-inflammatory macrophages	14,059
Anti-inflammatory alveolar macrophages	6,574
Activated adventitial fibroblasts	1,137
Fibroblasts	1,131
